# Supplementary material for: A systematic survey of natural language processing for the Greek language
Source: Patterns (N Y). 2025 Jul 21;6(11):101313. doi: 10.1016/j.patter.2025.101313 (PMC12715428; doi:10.1016/j.patter.2025.101313)
Supplement: Document S1. Table S1 and Notes S1–S4 [file mmc1.pdf]

Patterns, Volume *6*

Supplemental information

A systematic survey of natural language processing for the Greek language

Juli Bakagianni, Kanella Pouli, Maria Gavriilidou, and John Pavlopoulos

Supplemental Notes

Note S1. Quality Assurance Search Round

The search strategy for retrieving publications on Greek Natural Language Processing (NLP) research involved querying three databases using their APIs, with the query terms Greek (or Modern Greek) and “natural language processing” for the period January 2012 to December 2023 (Section Search Protocol).

To ascertain that we performed a comprehensive search, we conducted an additional round of searches on Google Scholar for the same time period. Google Scholar does not provide an API for automated data retrieval, which is why it was not included in the core search rounds. This supplementary search employed more specific query terms aimed at broadening our exploration, including specific NLP downstream tasks alongside “Greek” and either “Natural Language Processing” or “NLP”. Table S1 summarizes this supplementary search process.

We performed 23 queries, employing various alternatives, acronyms, and operators for selected NLP tasks from November 6th, 2023, to January 12th, 2024. Whenever the retrieved papers numbered less than 100, all were examined; otherwise, only the initial thirty were reviewed. The count of the top 30 papers seemed adequate, as beyond these, the subsequent papers didn’t appear to be relevant.

Table S1: The queries and the number of papers retrieved from Google Scholar during the quality assurance search round.

Query	Number of papers
“toxicity detection” “natural language processing” greek	64
“toxicity detection” greek nlp	70
“toxicity detection” + greek + nlp	40
“toxicity detection” and “greek” and “nlp”	32
“abusive language” “natural language processing” greek	420
“hateful language” “natural language processing” greek	48
“aggressive language” “natural language processing” greek	44
“authorship attribution” “natural language processing” greek	803
“authorship identification” “natural language processing” greek	330
“authorship analysis” “natural language processing” greek	324
“authorship detection” “natural language processing” greek	61
“sentiment analysis” greek nlp	7,490
“sentiment analysis” “natural language processing” greek	7,050
“sentiment analysis” “greek” “nlp”	2,430
“machine translation” greek nlp	9,160
“machine translation” “greek” “nlp”	3,750
“named entity recognition” “natural language processing” greek	4350
“named entity recognition” greek	5,550
“question answering” “natural language processing” greek	4,840
summarization “natural language processing” greek	16,600
semantics “natural language processing” greek	17,100
syntax “natural language processing” greek	14,400

The queries led us to review 698 publications. After removing duplicates both within the same round and across previous core search rounds, 357 publications were selected. Following the search process of the protocol, we kept those publications that referenced the term “Greek” in either the title or abstract. Additionally, for this particular round, we refined our approach to the term “Natural Language Processing” by including only those publications that explicitly mentioned the term within the publication’s content, excluding those where it appeared only in the references. In the subsequent filtering process (see Section Filtering Strategy), applying the exclusion criteria yielded five additional publications, which were included in the final list of surveyed papers.

Note S2. Natural Language Understanding and Generation

This Appendix first discusses Natural Language Understanding (NLU), which enables machines to understand natural language, and then Natural Language Generation (NLG), which is the process of generating meaningful text.[1]

Note S2.1. Natural Language Understanding

NLU enables machines to understand, interpret, and derive meaning from human language in a way that is both accurate and contextually relevant. It addresses a wide range of linguistic phenomena, from lexical semantics concerning aspects of word meaning to the high-level reasoning and application of world knowledge.[2] These linguistic phenomena are foundational for various NLP tasks, as they provide the essential linguistic comprehension needed to analyse the textual data.[3]

Traditional machine learning approaches represent texts by engineered features (e.g., based on POS tags or lexicons) or by character and word embeddings, encoding the linguistic knowledge into the input, before solving the task at hand. In the deep learning (DL) era, on the other hand, linguistic skills are encoded in neural network models via language modeling.[4] Therefore they are considered as an evaluation criterion, by assessing language models (LM) on datasets annotated for various linguistic phenomena. General-purpose NLU evaluation benchmark datasets exist primarily for English,[2,5? ? –7] but also for other languages.[8–13] Unfortunately, such monolingual NLU benchmarks are currently unavailable for Greek, based on our survey. There are multilingual NLU benchmarks, where the non-English language parts are translated either by humans, such as Belebele,[14] or automatically, such as ARC Greek.[?] However, even manual translations are distinct from naturally occurring data created by native speakers due to various factors, such as cultural nuances, idiomatic expressions, and context-specific references in native speakers' natural language usage.[15]

Note S2.2. Natural Language Generation

NLG involves the generation of human understandable natural language text from various data formats, such as text, structured data, video, and audio. NLG techniques are used in many downstream tasks, such as summarization, dialogue generation, Question Answering (QA), and Machine Translation (MT). Advancements in DL, particularly with Transformer-based LMs such as BART[16] (which follows the encoder-decoder neural architecture) and GPT-2[17] (which uses a decoder-only architecture), as well as the latest developments in large language models, such as the GPT, Llama, and Palm families, have fueled rapid progress in NLG (Section The DL Era). Alongside the advancement of NLG models, attention toward their limitations and potential risks has also increased.[18] An example is output degeneration,[19,20] which refers to generated output that is bland, incoherent, or gets stuck in repetitive loops. Another example is the generation of nonsensical text, or text that is unfaithful to the provided source input,[21,22] also known as hallucination.[23]

Note S3. Sentiment Analysis and Argument Mining

Sentiment, opinion, and emotion can be approached by various annotation schemas. Our work presents only the ones employed in the retrieved papers. Binary Sentiment Analysis (SA), where the text's polarity is either positive or negative, is the primary approach used in most papers cited in this study.[24–35] However, several studies[26,29,36–39] added a neutral class for texts carrying no opinion.

Five-class sentiment classification[40–43] increases the granularity, with scales from positive to negative. Subjectivity detection can precede SA, by addressing the binary task of whether the text comprises sentiment or not.[44] Instead of the coarse level of sentiment and subjectivity, however, one can also focus on fine-grained emotions,[37,44–46] using different emotion categories that have been suggested by theorists in psychology.[47? ? ?] Ekman has identified six emotions, i.e., anger, disgust, fear, joy, sadness, and surprise, as primary. A common approach, finally, concerns the selection of a single emotion or sentiment that is related to a specific condition, such as the anxiety during the COVID19 pandemic[27] or sarcasm/irony.[37,48,49]

A critical distinction between SA approaches is the level of granularity of the analysis. The coarser granularity concerns the document level, where the overall sentiment of an entire document is assessed. A finer granularity regards the sentence level, where the sentiment of short texts, such as tweets or the individual sentences within a document, is analyzed. SA can also be aspect-based, where the sentiment towards specific aspects or features of an entity is evaluated. These are described in detail below:

- **Document-level SA** involves the detection of the emotions expressed in an opinion document (e.g., a lengthy product review) or its classification as positive or negative (i.e., the sentiment polarity). Analysis at this level focuses on the sentiment of the entire document, as a whole (e.g., a product review), without considering entities or aspects within the document.

- **Sentence-level SA** concerns short texts, such as tweets or the sentences of a document. Although analysis at the sentence level is similar to that at the document level, the former can be more challenging due to the limited context contained. Also, classification at the sentence level cannot easily ignore the neutral class, because sentences with no opinion are more likely to appear. That is, even opinionated documents comprise sentences that bear no sentiment. Furthermore, a common (although implicit) assumption at research tasks

focusing on the sentence level is that each sentence expresses a single sentiment,[50] which, however, is not always the case.

- **Aspect-based SA**, also known as topic-based, entity-based, or target-based SA, as there is not a single term that sounds natural in every application domain,[51] is the analysis that focuses on the sentiment expressed in a document or sentence with respect to a specific aspect of an entity. For example, in the sentence "iPhone's battery lasts long" the entity is iPhone and the aspect is battery. Closely related to aspect-based SA is the **stance detection** task,[52] which aims to identify the stance (as in favor of or against) of the text author towards a target (person, organization, movement, policy, etc.) either explicitly mentioned or implied within the text.[53,54] Only a few published studies concern SA on Greek at this granularity.

- **Argument Mining** is the task of extracting natural language arguments and their relationships from text. The final objective is to provide machine-processable structured data for computational models of argument, to facilitate the automatic identification of reasoning capabilities upon the retrieved arguments and relations.[55]

Note S4. Toxicity Detection

Our study uses the term toxicity as a broader umbrella term of unwanted user-generated content including hateful and violent speech.[56] Although no standard definition exists today and some researchers are against oversimplifying solutions,[57] our choice is driven simply by the practical need to cover all the subtypes in a single name. However, the definition of toxicity itself is controversial: different cultures, languages, dialects, social groups, minority groups do not necessarily agree on what constitutes toxicity; furthermore, aggressive language use is not always toxic, e.g., inside a group, swear words can function as solidarity markers.

Supplemental References

1. Khurana, D., Koli, A., Khatter, K., and Singh, S. (2023). Natural language processing: State of the art, current trends and challenges. Multimedia tools and applications *82*, 3713–3744. doi: `https://doi.org/10.1007/s11042-022-13428-4`.

2. Wang, A., Singh, A., Michael, J., Hill, F., Levy, O., and Bowman, S. (2018). GLUE: A multi-task benchmark and analysis platform for natural language understanding. In T. Linzen, G. Chrupała, and A. Alishahi, eds. Proceedings of the 2018 EMNLP Workshop BlackboxNLP: Analyzing and Interpreting Neural Networks for NLP. Brussels, Belgium: Association for Computational Linguistics pp. 353–355. doi: `10.18653/v1/W18-5446`.

3. Bowman, S.R., and Dahl, G. (2021). What will it take to fix benchmarking in natural language understanding? In K. Toutanova, A. Rumshisky, L. Zettlemoyer, D. Hakkani-Tur, I. Beltagy, S. Bethard, R. Cotterell, T. Chakraborty, and Y. Zhou, eds. Proceedings of the 2021 Conference of the North American Chapter of the Association for Computational Linguistics: Human Language Technologies. Online: Association for Computational Linguistics pp. 4843–4855. doi: `10.18653/v1/2021.naacl-main.385`.

4. Belinkov, Y., Gehrmann, S., and Pavlick, E. (2020). Interpretability and analysis in neural nlp. In Proceedings of the 58th annual meeting of the association for computational linguistics: tutorial abstracts. pp. 1–5.

5. Bommasani, R., Liang, P., and Lee, T. (2023). Holistic evaluation of language models. Annals of the New York Academy of Sciences *1525*, 140–146.

6. Chen, Z., and Gao, Q. (2022). Curriculum: A broad-coverage benchmark for linguistic phenomena in natural language understanding. In M. Carpuat, de M.C. Marneffe, and I.V. Meza Ruiz, eds. Proceedings of the 2022 Conference of the North American Chapter of the Association for Computational Linguistics: Human Language Technologies. Seattle, United States: Association for Computational Linguistics pp. 3204–3219. doi: `10.18653/v1/2022.naacl-main.234`.

7. Nie, Y., Williams, A., Dinan, E., Bansal, M., Weston, J., and Kiela, D. (2019). Adversarial nli: A new benchmark for natural language understanding. Preprint at arXiv `https://doi.org/10.48550/arXiv.1910.14599`.

8. Urbizu, G., San Vicente, I., Saralegi, X., Agerri, R., and Soroa, A. (2022). Basqueglue: A natural language understanding benchmark for basque. In Proceedings of the Thirteenth Language Resources and Evaluation Conference. pp. 1603–1612.

9. Shavrina, T., Fenogenova, A., Anton, E., Shevelev, D., Artemova, E., Malykh, V., Mikhailov, V., Tikhonova, M., Chertok, A., and Evlampiev, A. (2020). RussianSuperGLUE: A Russian language understanding evaluation benchmark. In B. Webber, T. Cohn, Y. He, and Y. Liu, eds. Proceedings of the 2020 Conference on Empirical Methods in Natural Language Processing (EMNLP). Online: Association for Computational Linguistics pp. 4717–4726. doi: 10.18653/v1/2020.emnlp-main.381.

10. Wilie, B., Vincentio, K., Winata, G.I., Cahyawijaya, S., Li, X., Lim, Z.Y., Soleman, S., Mahendra, R., Fung, P., Bahar, S., and Purwarianti, A. (2020). IndoNLU: Benchmark and resources for evaluating Indonesian natural language understanding. In K.F. Wong, K. Knight, and H. Wu, eds. Proceedings of the 1st Conference of the Asia-Pacific Chapter of the Association for Computational Linguistics and the 10th International Joint Conference on Natural Language Processing. Suzhou, China: Association for Computational Linguistics pp. 843–857.

11. Xu, L., Hu, H., Zhang, X., Li, L., Cao, C., Li, Y., Xu, Y., Sun, K., Yu, D., Yu, C. et al. (2020). Clue: A chinese language understanding evaluation benchmark. In Proceedings of the 28th International Conference on Computational Linguistics. pp. 4762–4772.

12. Rybak, P., Mroczkowski, R., Tracz, J., and Gawlik, I. (2020). KLEJ: Comprehensive benchmark for Polish language understanding. In D. Jurafsky, J. Chai, N. Schluter, and J. Tetreault, eds. Proceedings of the 58th Annual Meeting of the Association for Computational Linguistics. Online: Association for Computational Linguistics pp. 1191–1201. doi: 10.18653/v1/2020.acl-main.111.

13. Ham, J., Choe, Y.J., Park, K., Choi, I., and Soh, H. (2020). Kornli and korsts: New benchmark datasets for korean natural language understanding. In Findings of the Association for Computational Linguistics: EMNLP 2020. pp. 422–430.

14. Bandarkar, L., Liang, D., Muller, B., Artetxe, M., Shukla, S.N., Husa, D., Goyal, N., Krishnan, A., Zettlemoyer, L., and Khabsa, M. (2023). The belebele benchmark: a parallel reading comprehension dataset in 122 language variants. Preprint at arXiv https://doi.org/10.48550/arXiv.2308.16884.

15. Rogers, A., Gardner, M., and Augenstein, I. (2023). Qa dataset explosion: A taxonomy of nlp resources for question answering and reading comprehension. ACM Comput. Surv. *55*, 1–45. doi: 10.1145/3560260.

16. Lewis, M., Liu, Y., Goyal, N., Ghazvininejad, M., Mohamed, A., Levy, O., Stoyanov, V., and Zettlemoyer, L. (2020). BART: Denoising sequence-to-sequence pre-training for natural language generation, translation, and comprehension. In Proceedings of the 58th Annual Meeting of the Association for Computational Linguistics. Online: Association for Computational Linguistics pp. 7871–7880.

17. Radford, A., Wu, J., Child, R., Luan, D., Amodei, D., Sutskever, I. et al. (2019). Language models are unsupervised multitask learners. OpenAI. https://openai.com/blog/language-unsupervised.

18. Ji, Z., Lee, N., Frieske, R., Yu, T., Su, D., Xu, Y., Ishii, E., Bang, Y.J., Madotto, A., and Fung, P. (2023). Survey of hallucination in natural language generation. ACM Computing Surveys *55*, 1–38. doi: https://doi.org/10.1145/3571730.

19. Holtzman, A., Buys, J., Du, L., Forbes, M., and Choi, Y. (2019). The curious case of neural text degeneration. Preprint at arXiv https://doi.org/10.48550/arXiv.1904.09751.

20. Welleck, S., Kulikov, I., Roller, S., Dinan, E., Cho, K., and Weston, J. (2019). Neural text generation with unlikelihood training. Preprint at arXiv https://doi.org/10.48550/arXiv.1908.04319.

21. Raunak, V., Menezes, A., and Junczys-Dowmunt, M. (2021). The curious case of hallucinations in neural machine translation. Preprint at arXiv https://doi.org/10.48550/arXiv.2104.06683.

22. Rohrbach, A., Hendricks, L.A., Burns, K., Darrell, T., and Saenko, K. (2018). Object hallucination in image captioning. Preprint at arXiv https://doi.org/10.48550/arXiv.1809.02156.

23. Maynez, J., Narayan, S., Bohnet, B., and McDonald, R. (2020). On faithfulness and factuality in abstractive summarization. Preprint at arXiv https://doi.org/10.48550/arXiv.2005.00661.

24. Bilianos, D. (2022). Experiments in text classification: Analyzing the sentiment of electronic product reviews in greek. Journal of Quantitative Linguistics *29*, 374–386. doi: 10.1080/09296174.2021.1885872.

25. Kapoteli, E., Koukaras, P., and Tjortjis, C. (2022). Social media sentiment analysis related to covid-19 vaccines: Case studies in english and greek language. In I. Maglogiannis, L. Iliadis, J. Macintyre, and P. Cortez, eds. Artificial Intelligence Applications and Innovations. Cham: Springer International Publishing. ISBN 978-3-031-08337-2 pp. 360–372. doi: https://doi.org/10.1007/978-3-031-08337-2_30.

26. Alexandridis, G., Varlamis, I., Korovesis, K., Caridakis, G., and Tsantilas, P. (2021). A survey on sentiment analysis and opinion mining in greek social media. Information *12*. doi: 10.3390/info12080331.

27. Kydros, D., Argyropoulou, M., and Vrana, V. (2021). A content and sentiment analysis of greek tweets during the pandemic. Sustainability *13*, 6150. doi: 10.3390/su13116150.

28. Braoudaki, A., Kanellou, E., Kozanitis, C., and Fatourou, P. (2020). Hybrid data driven and rule based sentiment analysis on greek text. Procedia Computer Science *178*, 234–243. doi: https://doi.org/10.1016/j.procs.2020.11.025. 9th International Young Scientists Conference in Computational Science, YSC2020, 05-12 September 2020.

29. Beleveslis, D., Tjortjis, C., Psaradelis, D., and Nikoglou, D. (2019). A hybrid method for sentiment analysis of election related tweets. In 2019 4th South-East Europe Design Automation, Computer Engineering, Computer Networks and Social Media Conference (SEEDA-CECNSM). pp. 1–6. doi: 10.1109/SEEDA-CECNSM.2019.8908289.

30. Medrouk, L., and Pappa, A. (2018). Do deep networks really need complex modules for multilingual sentiment polarity detection and domain classification? In 2018 International Joint Conference on Neural Networks (IJCNN). pp. 1–6. doi: 10.1109/IJCNN.2018.8489613.

31. Medrouk, L., and Pappa, A. (2017). Deep learning model for sentiment analysis in multi-lingual corpus. In D. Liu, S. Xie, Y. Li, D. Zhao, and E.S.M. El-Alfy, eds. Neural Information Processing. Cham: Springer International Publishing. ISBN 978-3-319-70087-8 pp. 205–212. doi: https://doi.org/10.1007/978-3-319-70087-8_22.

32. Athanasiou, V., and Maragoudakis, M. (2017). A novel, gradient boosting framework for sentiment analysis in languages where nlp resources are not plentiful: A case study for modern greek. Algorithms *10*. doi: 10.3390/a10010034.

33. Giatsoglou, M., Vozalis, M.G., Diamantaras, K., Vakali, A., Sarigiannidis, G., and Chatzisavvas, K.C. (2017). Sentiment analysis leveraging emotions and word embeddings. Expert Systems with Applications *69*, 214–224. doi: https://doi.org/10.1016/j.eswa.2016.10.043.

34. Charalampakis, B., Spathis, D., Kouslis, E., and Kermanidis, K. (2016). A comparison between semi-supervised and supervised text mining techniques on detecting irony in greek political tweets. Engineering Applications of Artificial Intelligence *51*, 50–57. doi: https://doi.org/10.1016/j.engappai.2016.01.007. Mining the Humanities: Technologies and Applications.

35. Markopoulos, G., Mikros, G., Iliadi, A., and Liontos, M. (2015). Sentiment analysis of hotel reviews in greek: A comparison of unigram features. In V. Katsoni, ed. Cultural Tourism in a Digital Era. Cham: Springer International Publishing. ISBN 978-3-319-15859-4 pp. 373–383.

36. Drakopoulos, G., Giannoukou, I., Mylonas, P., and Sioutas, S. (2020). A graph neural network for assessing the affective coherence of twitter graphs. In 2020 IEEE International Conference on Big Data (Big Data). IEEE pp. 3618–3627. doi: 10.1109/BigData50022.2020.9378492.

37. Tsakalidis, A., Papadopoulos, S., Voskaki, R., Ioannidou, K., Boididou, C., Cristea, A.I., Liakata, M., and Kompatsiaris, Y. (2018). Building and evaluating resources for sentiment analysis in the greek language. Language resources and evaluation *52*, 1021–1044. doi: https://doi.org/10.1007/s10579-018-9420-4.

38. Makrynioti, N., and Vassalos, V. (2015). Sentiment extraction from tweets: Multilingual challenges. In S. Madria, and T. Hara, eds. Big Data Analytics and Knowledge Discovery. Cham: Springer International Publishing. ISBN 978-3-319-22729-0 pp. 136–148. doi: https://doi.org/10.1007/978-3-319-22729-0_11.

39. Petasis, G., Spiliotopoulos, D., Tsirakis, N., and Tsantilas, P. (2014). Sentiment analysis for reputation management: Mining the greek web. In Artificial Intelligence: Methods and Applications: 8th Hellenic Conference on AI, SETN 2014, Ioannina, Greece, May 15-17, 2014. Proceedings 8. Springer pp. 327–340. doi: https://doi.org/10.1007/978-3-319-07064-3_26.

40. Spatiotis, N., Perikos, I., Mporas, I., and Paraskevas, M. (2020). Sentiment analysis of teachers using social information in educational platform environments. International Journal on Artificial Intelligence Tools *29*, 1–29. doi: https://doi.org/10.1142/S0218213020400047.

41. Spatiotis, N., Perikos, I., Mporas, I., and Paraskevas, M. (2019). Examining the impact of discretization technique on sentiment analysis for the greek language. In 2019 10th International Conference on Information, Intelligence, Systems and Applications (IISA). pp. 1–6. doi: 10.1109/IISA.2019.8900699.

42. Spatiotis, N., Paraskevas, M., Perikos, I., and Mporas, I. (2017). Examining the impact of feature selection on sentiment analysis for the greek language. In Speech and Computer: 19th International Conference, SPECOM 2017, Hatfield, UK, September 12-16, 2017, Proceedings 19. Springer pp. 353–361. doi: https://doi.org/10.1007/978-3-319-66429-3_34.

43. Spatiotis, N., Mporas, I., Paraskevas, M., and Perikos, I. (2016). Sentiment analysis for the greek language. In Proceedings of the 20th Pan-Hellenic Conference on Informatics. PCI '16. New York, NY, USA: Association for Computing Machinery. ISBN 9781450347891 pp. 1–4.

44. Solakidis, G.S., Vavliakis, K.N., and Mitkas, P.A. (2014). Multilingual sentiment analysis using emoticons and keywords. In 2014 IEEE/WIC/ACM International Joint Conferences on Web Intelligence (WI) and Intelligent Agent Technologies (IAT) vol. 2. IEEE pp. 102–109. doi: 10.1109/WI-IAT.2014.86.

45. Alexandridis, G., Korovesis, K., Varlamis, I., Tsantilas, P., and Caridakis, G. (2022). Emotion detection on greek social media using bidirectional encoder representations from transformers. In 25th Pan-Hellenic Conference on Informatics. PCI 2021. New York, NY, USA: Association for Computing Machinery. ISBN 9781450395557 pp. 28–32. doi: 10.1145/3503823.3503829.

46. Chatzakou, D., Vakali, A., and Kafetsios, K. (2017). Detecting variation of emotions in online activities. Expert Systems with Applications *89*, 318–332. doi: https://doi.org/10.1016/j.eswa.2017.07.044.

47. Kafetsios, K., and Nezlek, J.B. (2012). Emotion and support perceptions in everyday social interaction: Testing the "less is more" hypothesis in two cultures. Journal of Social and Personal Relationships *29*, 165–184. doi: https://doi.org/10.1177/0265407511420194.

48. Antonakaki, D., Spiliotopoulos, D., V. Samaras, C., Pratikakis, P., Ioannidis, S., and Fragopoulou, P. (2017). Social media analysis during political turbulence. PloS one *12*, e0186836. doi: https://doi.org/10.1371/journal.pone.0186836.

49. Antonakaki, D., Spiliotopoulos, D., Samaras, C.V., Ioannidis, S., and Fragopoulou, P. (2016). Investigating the complete corpus of referendum and elections tweets. In 2016 IEEE/ACM International Conference on Advances in Social Networks Analysis and Mining (ASONAM). IEEE pp. 100–105. doi: 10.1109/ASONAM.2016.7752220.

50. Liu, B. (2020). Sentiment analysis: Mining opinions, sentiments, and emotions. Cambridge university press.

51. Schouten, K., and Frasincar, F. (2015). Survey on aspect-level sentiment analysis. IEEE Transactions on Knowledge and Data Engineering *28*, 813–830. doi: 10.1109/TKDE.2015.2485209.

52. Küçük, D., and Can, F. (2020). Stance detection: A survey. ACM Computing Surveys (CSUR) *53*, 1–37. doi: https://doi.org/10.1145/3369026.

53. Mohammad, S., Kiritchenko, S., Sobhani, P., Zhu, X., and Cherry, C. (2016). Semeval-2016 task 6: Detecting stance in tweets. In Proceedings of the 10th international workshop on semantic evaluation (SemEval-2016). pp. 31–41. doi: 10.18653/v1/S16-1003.

54. Sobhani, P. Stance detection and analysis in social media. Ph.D. thesis Universite d'Ottawa/University of Ottawa (2017).

55. Cabrio, E., and Villata, S. (2018). Five years of argument mining: A data-driven analysis. In IJCAI vol. 18. pp. 5427–5433. doi: https://doi.org/10.24963/ijcai.2018/766.

56. Waseem, Z., Davidson, T., Warmsley, D., and Weber, I. (2017). Understanding abuse: A typology of abusive language detection subtasks. Preprint at arXiv https://doi.org/10.48550/arXiv.1705.09899.

57. Díaz, M., Amironesei, R., Weidinger, L., and Gabriel, I. (2022). Accounting for offensive speech as a practice of resistance. In Proceedings of the Sixth Workshop on Online Abuse and Harms (WOAH). pp. 192–202.
