# Supplementary material for: A systematic survey of natural language processing for the Greek language
Source: Patterns (N Y). 2025 Jul 21;6(11):101313. doi: 10.1016/j.patter.2025.101313 (PMC12715428; doi:10.1016/j.patter.2025.101313)
Supplement: Document S2. Article plus supplemental information [file mmc2.pdf]

Patterns

Article

A systematic survey of natural language processing for the Greek language

Graphical abstract

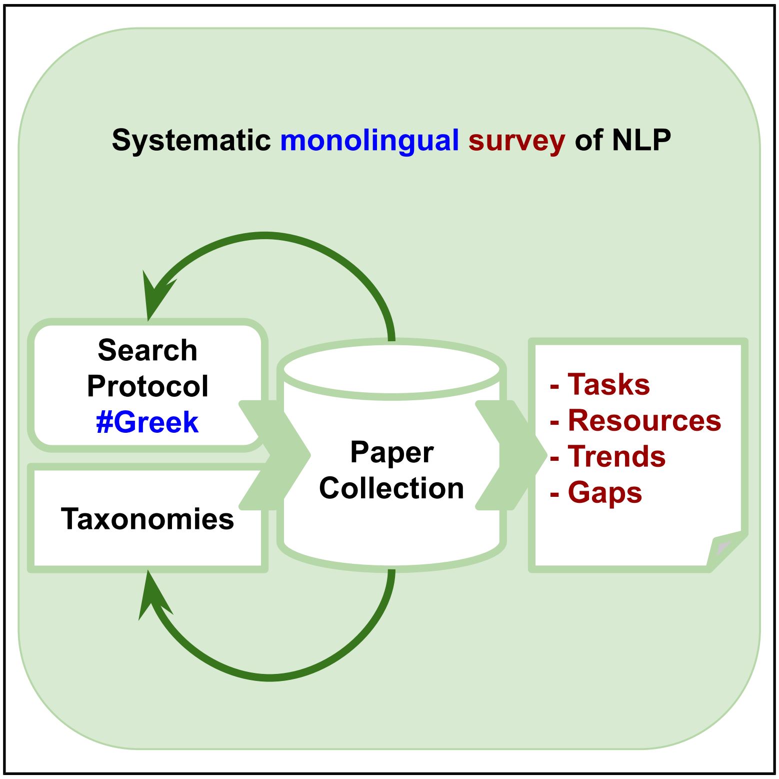

Authors

Juli Bakagianni, Kanella Pouli, Maria Gavriilidou, John Pavlopoulos

Correspondence

annis@aueb.gr

In brief

Many languages remain under-represented in AI research, limiting the global reach of language technologies. This study introduces a clear, repeatable method for surveying natural language processing (NLP) work in individual languages. When the method is applied to Greek (2012–2023), the survey reveals key trends, gaps, and resource needs—insights that are essential for fairer and more effective language technology. The approach offers a blueprint for mapping NLP progress in other under-resourced languages, helping to build a more inclusive multilingual AI landscape.

Highlights

- Greek is moderately supported in NLP
- Monolingual language models (LMs) are preferred over multilingual ones
- Task-specific trends differ notably from global trends

Bakagianni et al., 2025, Patterns *6*, 101313
November 14, 2025 © 2025 The Authors. Published by Elsevier Inc.
https://doi.org/10.1016/j.patter.2025.101313

CellPress

CellPress
OPEN ACCESS

Article

A systematic survey of natural language processing for the Greek language

Juli Bakagianni,[1] Kanella Pouli,[2] Maria Gavriilidou,[2] and John Pavlopoulos[1,3,4,5,*]
[1]Department of Informatics, Athens University of Economics and Business, 10434 Athens, Greece
[2]Institute for Language and Speech Processing, Athena Research Center, 15125 Athens, Greece
[3]Archimedes, Athena Research Center, 15125 Athens, Greece
[4]Department of Computer and Systems Sciences, Stockholm University, 16455 Kista, Sweden
[5]Lead contact
*Correspondence: annis@aueb.gr
https://doi.org/10.1016/j.patter.2025.101313

THE BIGGER PICTURE Natural language processing (NLP) methods have advanced rapidly, yet research remains centered on English, followed by a handful of well-supported languages, while many others receive only moderate, limited, or no support. This imbalance affects the adaptability of language technologies across diverse linguistic and cultural contexts. Comprehensive monolingual NLP surveys—those covering the full spectrum of NLP tasks—offer a structured way to assess language-specific challenges, resource availability, and methodological trends, yet no standardized framework exists for conducting these surveys. We introduce a generalizable methodology for systematic monolingual NLP surveys, which ensures transparency and reproducibility. The method can be extended to other languages with similar challenges, ultimately contributing to a more inclusive and equitable NLP landscape.

SUMMARY

Comprehensive monolingual natural language processing (NLP) surveys are essential for assessing language-specific challenges, resource availability, and research gaps. However, existing surveys often lack standardized methodologies, leading to selection bias and fragmented coverage of NLP tasks and resources. This study introduces a generalizable framework for systematic monolingual NLP surveys. Our approach integrates a structured search protocol to minimize bias, an NLP task taxonomy for classification, and language resource taxonomies to identify potential benchmarks and highlight opportunities for improving resource availability. We apply this framework to Greek NLP (2012–2023), providing an in-depth analysis of its current state, task-specific progress, and resource gaps. The survey results are publicly available and are regularly updated to provide an evergreen resource. This systematic survey of Greek NLP serves as a case study, demonstrating the effectiveness of our framework and its potential for broader application to other not-so-well-resourced languages as regards NLP.

INTRODUCTION

Natural language processing (NLP) focuses on the computational processing of human languages, enabling machines to understand and generate natural language. Recently, several NLP tasks have advanced significantly with the help of deep learning (DL)[1] and more recently with large language models (LLMs).[2] Multilingual NLP has benefited from these advances[3,4]; however, by focusing on progress per language, we observe that well-supported languages benefit considerably more compared to the rest.[5] As a result, NLP for the myriad of languages worldwide relies heavily on research conducted for well-supported languages, often inheriting their assumptions, biases, and other characteristics that may not align with their unique linguistic features,[6] thereby limiting equitable technological access.

Monolingual NLP surveys offer a pathway to address these disparities by synthesizing language-specific challenges (e.g., scarce annotated data and morphological complexity), auditing resources and methodological adaptations, and identifying research gaps that hinder equitable progress. However, their utility depends on systematic rigor: reproducible search protocols and transparent filtering criteria minimize selection bias and ensure replicable results, while organizing surveyed material into coherent NLP thematic tracks, such as syntax and information extraction (IE), enables structured analysis of task-specific challenges, gaps, and trends. This structured presentation also

This is an open access article under the CC BY license (http://creativecommons.org/licenses/by/4.0/).

supports cross-task comparisons, revealing overarching insights, such as state-of-the-art models across tasks. Furthermore, systematically documenting language resources (LRs)—including their availability, annotation status (e.g., raw, human-annotated), and annotation type (e.g., automatically labeled)—identifies potential benchmarks that can be used for pre-training, fine-tuning, and assessing NLP models, without inheriting the assumptions and biases of well-supported languages. This process also highlights critical shortages, such as annotated datasets for understudied tasks. Although monolingual NLP surveys exist,[7–18] and their contributions are valuable, they do not share the surveying methods they followed, such as the search protocol, risking selection bias, and fragmented coverage of tasks and resources. To our knowledge, no generalized framework exists to standardize monolingual survey design, hindering actionable progress for less-supported languages.

In this work, we bridge this gap by (1) proposing a generalizable methodology for systematic monolingual NLP surveys and (2) applying it to Greek, a language characterized as a low-resource language for several NLP tasks.[19–22] We demonstrate how our framework—tested through a comprehensive review of Greek NLP—enables researchers to identify language-specific challenges, evaluate resource availability, and prioritize future work efficiently. Our survey of Greek NLP research is focused on studies published between 2012 and 2023. This time frame marks transformative advancements in NLP (e.g., the shift from machine learning [ML] to DL and LLMs) and societal shifts driven by GenA's digital-native upbringing. Our analysis captured how Greek NLP evolved alongside these technological and generational trends. Using our systematic search protocol, we retrieved over a thousand research studies on Greek NLP, of which 142 met the specific criteria outlined in our search protocol. This survey offers both task-specific insights and an overview of overarching trends in Greek NLP.

Our findings highlight the following aspects:

(1) Greek is moderately supported in NLP. We identified nine publicly available, human-annotated datasets related to nine distinct NLP tasks, including Summarization, Named Entity Recognition (NER), Intent Classification, Topic Classification, Grammatical Error Correction (GEC), Toxicity Detection, Syntactical and Morphological Analysis, Machine Translation (MT), and Text Classification. These resources hold significant potential as benchmarks for advancing Greek NLP research. This observation positions Greek as a moderately supported language in NLP and is also aligned with a language support classification system we developed that classifies languages based on their coverage in Association for Computational Linguistics (ACL) publications, which also classifies Greek as a moderately supported language.
(2) Resource gaps exist despite cross-lingual innovations. Despite progress, benchmarks for certain NLP tasks, such as sentiment analysis (SA), are missing. However, our systematic cataloging identified 17 datasets that—with added licenses or improved maintenance—could serve as benchmarks. Cross-lingual techniques, such as translation strategies outperforming multilingual encoders,[22] offer practical pathways to mitigate data scarcity, and therefore we summarize and highlight these efforts.
(3) Methodological shifts reveal lingering gaps. The research landscape in Greek NLP has shifted from traditional ML methods, which dominated until 2018, to the increasing adoption of DL approaches since 2019. Despite this shift, ML methods continue to dominate certain tasks, such as authorship analysis, question answering (QA), and semantics, indicating that these areas require further DL innovation. Conversely, newer trends for Greek, such as IE, ethics and NLP, and summarization, are increasingly dominated by DL approaches, with Greek included also in shared tasks for the latter two fields.
(4) Monolingual language models (LMs) are preferred over multilingual ones. Despite the global emphasis on multilingual systems, such as XLM-RoBERTa (XLM-R) and multilingual BERT (mBERT), few studies in Greek NLP are found to use them.[23–27] Greek NLP favors monolingual LMs, such as GreekBERT,[25] which achieves state-of-the-art results in several studies addressing different tasks.
(5) Task-specific trends differ notably from global trends. While Greek research aligns with global NLP trends in tasks such as SA, where NLP research declines,[28] this is not true in areas such as syntax, where Greek NLP retains interest despite a global decline in syntax-related research.

In what follows, we first provide the background of the present work (background). In this section, we discuss the support level of human languages within the NLP community and the characteristics of the Greek language (the language). Also, we discuss the examined time frame along with an exploration of the methodological shifts occurring during this time period (the time period of the survey), and we present the related work (NLP surveys). Next, we present our approach (methods), consisting of the search protocol (search protocol) and the taxonomies adopted for tasks, LRs availability, and annotation type (the taxonomies). Subsequently, we present the main outcomes of our study, organized by NLP thematic areas: ML for NLP (track: machine learning for NLP), Syntax and Grammar (track: syntax and grammar), Semantics (track: semantics), IE (track: information extraction), SA (track: sentiment analysis and argument mining), Authorship Analysis (track: authorship analysis), Ethics and NLP (track: ethics and NLP), summarization (track: summarization), QA (track: question answering), MT (track: machine translation, multilingualism, and cross-lingual NLP), and NLP applications that are not classifiable in any of the previous tracks (track: NLP applications). Lastly, we discuss the outcomes of this study with remarks on the limitations, and our final observations (discussion), followed by our conclusions. Each of the sections presenting the main outcomes of this survey (from track: machine learning for NLP to track: NLP applications) is structured as follows: first, we describe the track within its global context; we then discuss the methods identified by our study and the LRs produced; finally, each section concludes with a summary of the track and relevant observations.

CellPress
OPEN ACCESS

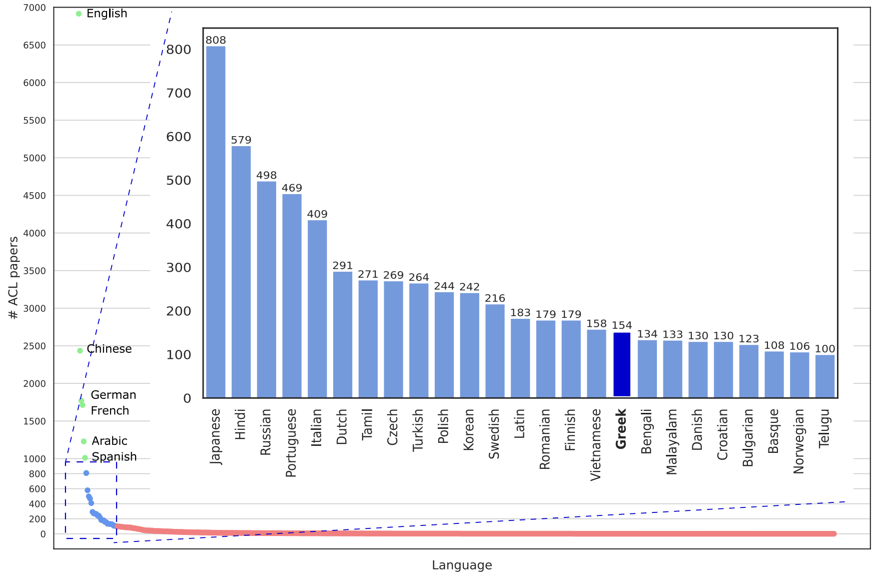

Figure 1. Number of publications in the ACL Anthology per language (shown vertically), with languages referenced in the title or abstract (horizontally)

We use the collection of languages outlined in the IETF BCP 47 language tag (RFC 5646).[30] The vast majority of languages appear in none (7,312 languages) or fewer than 100 publications (574 languages), depicted by a long red tail on the lowermost part of the figure. We refer to this group of languages as the third tier, which consists of less-supported languages. The second tier, shown in blue in the same distribution, is presented with additional detail in the upper-right part of the figure. This tier comprises moderately supported languages, which appear in between 100 and 1,000 publications, with Greek specifically represented in 154 publications. The first tier comprises well-supported languages, each referenced in more than 1,000 publications: English (referenced in 6,915 papers), Chinese (in almost 2,500), German and French (around 1,750), Arabic (1,229), and Spanish (1,011).

BACKGROUND

The language

Human languages

Human languages encompass a rich tapestry, totaling 7,916, as cataloged by ISO 639-3, an international standard that assigns unique codes to represent languages, including living, extinct, ancient, historic, and constructed ones. Despite this linguistic diversity, NLP research exhibits significant imbalances, with English dominating the field. To assess the level of support for different languages in the NLP field, we conducted an analysis of the ACL Anthology, an authoritative hub of computational linguistics and NLP research. Specifically, we counted papers published between January 2012 and January 2024 that reference each language listed in the Internet Engineering Task Force (IETF) Best Current Practice (BCP) 47 standard in their titles or abstracts. Languages were classified into three tiers based on the number of publications: well supported, moderately supported, and lowly supported.

As shown in Figure 1, English is the most-studied language, with 6,915 publications. This figure likely underestimates the true volume, as it is common practice in the NLP community not to explicitly mention English when it is the language of study.[29] Chinese, German, French, Arabic, and Spanish are also well supported, each with thousands of publications. Moderately supported languages, including Greek, constitute the second tier, with publication counts ranging from 100 to 1,000 per language. In contrast, 574 languages fall into the third tier, with 1–100 publications, while 7,312 languages are entirely unsupported.

This study focuses on Greek, a second-tier language among 25 others with moderate NLP research interest (100–1,000 references). Within this group, Greek ranks 17th by publication count (154 papers). However, when adjusting for total speaker populations (including native and second-language speakers), Greek rises to the 10th place. Speaker population data were sourced from SIL International,[31] and support per speaker population was calculated by dividing publication counts by speaker populations. Latin was excluded as an extinct language. This adjustment provides a more nuanced perspective by incorporating both research output and the size of the speaker base.

The Greek language

Understanding the linguistic characteristics of a language can help NLP researchers understand the specific challenges and opportunities for developing and applying NLP technologies in this language context. Greek, or Modern Greek, to differentiate it from earlier historical stages, is the official language of Greece and one of the two official languages of Cyprus. It is the mother tongue of approximately 95% of the 10.5 million inhabitants of Greece and of the approximately 500,000 Greek Cypriots. It is also used by approximately five million people of Greek origin worldwide as heritage language.[32]

The Greek alphabet has been the main script for writing Greek for most of the language's recorded history.[33] The use of the standard variety in education and mass media has led to the prevalence of Standard Modern Greek over various dialects. Henceforth, the term "Greek" is used to refer to Standard Modern Greek, which is a highly inflected language. It has four cases for the nominal system, two numbers, and three genders. The verb conjugation system is even more complex, with multiple tenses, moods, voices, different suffixes per person, and many irregularities. Word length is an additional factor differentiating Greek from other languages, most notably English. The majority of the Greek words, typically, have two or three syllables, but words with more syllables (e.g., eight or nine) are also not rare.[34] Moreover, Greek, unlike English, exhibits significant flexibility in word order. Its system of rich nominal inflection allows syntactic relations among clausal elements to be identified without requiring fixed positions. For instance, a simple declarative clause containing a verb, its nominal subject, and object can be constructed in all six logically possible combinations.[35]

The time period of the survey

The selected time span for our survey (2012–2023) aimed to capture the evolution of research methodologies in NLP in response

to global technological advancements and shifts in the field. The period under investigation witnessed a transition from traditional ML to DL. As Manning[36] stated: "DL waves have lapped at the shores of computational linguistics for several years now, but 2015 seems like the year when the full force of the tsunami hit the major NLP conferences." We aim to explore how this methodological shift influenced research on Greek. In the following sections we present a brief historical overview of the scientific field itself (i.e., by disregarding the target language) and its evolution over the years under study. The methods applied to the Greek language are outlined in track: machine learning for NLP.

The ML era

The predominant approach to NLP research in 2012, marking the beginning of our study period, primarily relied on traditional ML algorithms. Traditional ML focuses on developing algorithms and models that learn statistical patterns from data to make predictions or decisions. Unlike DL, which automates the feature extraction process through layered neural architectures, traditional ML is highly dependent on manual feature engineering. In traditional ML, relevant features are extracted, selected, or created from raw data to improve model performance. Commonly employed features include character or word tokens (unigrams or n-grams) and their frequency, often using methods such as frequency counts or the term frequency-inverse document frequency (TF-IDF) weighting scheme. Lexicon-based features, such as lists of words with specific meanings (e.g., sentiment lexicons), are also common.

The DL era

The surge of DL in NLP can be attributed to its ability to automatically learn hierarchical representations of data, eliminating the need for extensive feature engineering.[37] Coupled with the availability of vast amounts of data and increased computational power, DL has enabled more effective handling of complex linguistic structures. As a result, DL has demonstrated superior performance across various NLP tasks.[38] These advancements have led to the development of pre-trained language models (PLMs), which are neural-network-based statistical LMs.[39]

PLMs are task agnostic and follow a pre-training and fine-tuning paradigm, whereby LMs are pre-trained on web-scale unlabeled text corpora for general tasks such as word prediction and then fine-tuned to specific tasks using small amounts of (labeled) task-specific data.[39] Initially, models such as recurrent neural networks (RNNs)[40] were used for these purposes. RNNs, proposed in the 1980s for modeling time series,[41–43] are designed to explore temporal correlations between distant elements in the text.

The introduction of the Transformer architecture was a major milestone in NLP. Transformers[1] use self-attention mechanisms to compute attention scores for each word in a sentence, allowing for greater parallelization compared to RNN.[39] Transformer-based PLMs are categorized into three main types based on their neural architectures: encoder-only, decoder-only, and encoder-decoder models. Encoder-only models, such as BERT[44] and its variants (RoBERTa,[45] ALBERT,[46] DeBERTa,[47] XLM,[48] XLM-R,[49] and XLNet)[50] are primarily used for language-understanding tasks such as text classification. A detailed discussion on the distinction between natural language understanding (NLU) and natural language generation (NLG) can be found in Note S2. The fascination with the inner workings of these Transformer-based models has led to the emergence of a trend known as BERTology.[51] Decoder-only models, including GPT-1[52] and GPT-2[53] from OpenAI, focus on language-generation tasks. Encoder-decoder models, such as T5,[54] mT5,[55] and BART,[56] are versatile and can perform both understanding and generation tasks by framing them as sequence-to-sequence problems.

Finally, LLMs refer to transformer-based PLMs with tens to hundreds of billions of parameters. These models are not only larger in size but also exhibit stronger language understanding and generation capabilities compared to smaller models mentioned earlier.[39] Notable LLM families include OpenAI's GPT, Meta's open-source Llama, and Google's PaLM and Gemini. Other representative LLMs include FLAN,[57] Gopher,[58] T0,[59] and GLaM,[60] among others.

NLP surveys

Greek NLP surveys

Through our search protocol (search protocol), we identified other Greek NLP surveys—both comprehensive and domain-specific—which we discuss here. First, Papantoniou and Tzitzikas[11] provided a brief survey of NLP for the Greek language covering Ancient Greek, Modern Greek, and various dialects. This survey included the work of 99 papers published from 1990 to 2020. The authors addressed text, video, and image modalities. For text modality, they presented papers on tasks such as phonology, syntax, semantics, IE, SA, argument mining, QA, MT, and NLP applications. For image modality they outlined optical character recognition (OCR), and for video modality they discussed lip reading and keyword spotting. Regarding LRs, they presented a limited number of LRs, specifically three online lexica, five online corpora, two downloadable datasets, five tools, and one service. Giarelis et al.[61] provided an overview of state-of-the-art research in Greek NLP and chatbot applications published since 2018, establishing the search protocol they used. They reported on three DL LMs, two embedding-based techniques, and nine DL NLP applications, detailing the relevant datasets. For chatbot applications, they identified and reviewed five papers. Additionally, they offered insights into NLP models and chatbot implementation methodologies.

The remaining three surveys are purely domain specific. Nikiforos et al.[62] provided an extensive review of 49 papers published from 2012 to 2020 related to the Social Web in Modern Greek, Greek dialects, and Greeklish script. The NLP tasks covered include argument mining, authorship attribution, gender identification, offensive language detection, and SA. The authors systematically addressed the scientific contributions and unresolved issues of the reviewed papers. They also presented two tools and 21 datasets extracted from the surveyed papers, providing detailed information and links where available. Alexandridis et al.[63] reviewed 14 papers published from 2014 to 2020 that focus specifically on SA and opinion mining in Greek social media. The authors discussed the methods, tools, datasets, lexical resources, and models used for SA and opinion mining in Greek texts. Finally, Krasadakis et al.[64] surveyed 43 papers related to legal NLP published from 2012 to 2021. The survey covered tasks such as NER, entity linking, text segmentation, summarization, MT, rationale extraction, judgment prediction, and QA.

CellPress
OPEN ACCESS

Monolingual NLP surveys in other languages

Beyond Greek, we found that comprehensive monolingual NLP surveys are relatively rare. We searched the literature for surveys or overviews that cover a broad range of NLP tasks—similar in scope to our research—for well- and moderately supported languages, as classified in our tier system (human languages). Our search process involved querying Google Scholar for publications published between January 2012 and September 2023, using a specific query pattern. We searched for the name of the language of interest along with the keyword "Natural Language Processing," and either "survey" or "overview."

Notably, we found that only 19% of well- and moderately supported languages have peer-reviewed comprehensive monolingual NLP surveys. Among the six well-referenced languages, only Arabic, a macro-language that encompasses various individual varieties, has dedicated NLP surveys.[8–10,15,16] Of the 25 moderately referenced languages, five have peer-reviewed surveys, i.e., Tamil,[17] Turkish,[7] Finnish,[13] Greek,[11] and Basque.[18] Additionally, two languages, Hindi[14] and Bengali,[12] have preprints available.

Limitations in existing NLP surveys

The surveys mentioned above provide valuable insights into the languages they study; however, none disclose their search protocol, except for the domain-specific work of Giarelis et al.[61] This lack of transparency makes it difficult to assess the reproducibility of the surveys and understand the criteria and rationale behind the inclusion of specific papers. Additionally, it is unclear whether the NLP tasks presented fully encompass the research conducted in the language or if the papers were manually selected to fit the chosen tasks. Similarly, while some surveys provide information about the LRs available for the examined language, it is often unclear why certain LRs were selected and whether they are accessible and properly licensed.

METHODS

This section outlines the methodology proposed for constructing monolingual NLP surveys. It includes the search protocol (search protocol) applied to Greek NLP research as well as the taxonomies of tasks and LRs (the taxonomies).

Search protocol

We developed a comprehensive search protocol to identify peer-reviewed research papers related to NLP in the Greek language. Our goal was to create a process that is adaptable to any language and any publication time period. The protocol includes a search strategy for automatically locating relevant papers (search strategy) and a filtering process based on well-defined criteria (filtering strategy). It uses both bibliographic metadata and additional metadata collected to support the surveying process (metadata extraction).

Search strategy

Scientific databases. We used three reputable scientific databases to identify research papers related to NLP for Greek, published between January 2012 and December 2023. The selected databases are ACL Anthology,[65] a hub for computational linguistics and NLP research; Semantic Scholar,[66] an AI-powered search engine prioritizing computer science and related fields; and Scopus,[67] a globally recognized database. These databases were chosen not only for their reputability but also for their automated publication retrieval capabilities: Semantic Scholar and Scopus offer APIs, while ACL Anthology provides publication metadata in XML format through its GitHub repository.[68]

Querying process. The search was conducted using tailored query terms across ACL Anthology, Scopus, and Semantic Scholar, adapting to the search capabilities of each database. Scopus allows searching in the title, abstract, and full text (including references); Semantic Scholar searches across the entire paper content; and ACL Anthology limits the search to the title and abstract. Therefore, we focused our search on the language name, i.e., "Greek" or "Modern Greek," in the title or abstract of the papers and the term "Natural Language Processing" in the entire paper (where feasible). This approach was chosen because papers focused on a specific language are likely to mention the language name in these sections, thereby reducing the retrieval of false-positive papers (see challenges for monolingual NLP surveys). Specifically, Scopus employs Lucene queries, allowing us to search for the language name in titles and abstracts, and the term "Natural Language Processing" across the entire paper. Semantic Scholar does not offer specific search area options, so we used combined keywords with the + operator (AND), initially searching broadly and subsequently filtering results where the language name appeared in the title or abstract. For the ACL Anthology, which is dedicated to NLP, we limited our search to the language name in the title or abstract.

Core search rounds. The search process comprised four rounds, with the first three being core rounds, as detailed in Table 1. The first two core rounds focused on papers published between 2012 and 2022 and differed in the language query terms used. In the first core round, we searched using "Modern Greek," but due to its limited usage, we shifted to "Greek" in the second core round to capture a wider range of relevant papers. The language-specific filtering was then applied during the filtering process stage. The third core round focused on papers published in 2023 to incorporate more recent relevant work. Unlike the earlier rounds—which were exploratory and iterative, helping to shape the survey design—this round was conducted several months later, after the finalization of our survey methodology. As such, it served as a test case for our methodology, assessing the time and the effort needed to integrate new papers into the survey. Incorporating papers from this round was one-third faster, highlighting how a well-defined monolingual survey methodology, such as the one we propose, can significantly improve efficiency and scalability for future surveys.

Quality assurance round. The fourth round served as a supplementary phase for quality assurance of our search strategy and to validate the comprehensiveness of the selected query terms during the previous core search rounds. The objectives were 2-fold: first, to verify that the selected queries terms retrieved all relevant publications related to NLP research in the Greek language; and second, to address any potential gaps from excluding Google Scholar[69] in the core rounds. Despite its widespread usage, Google Scholar was not included in the core rounds due to its lack of an API for automated publication retrieval. In this phase, we cherry-picked specific NLP downstream tasks, such as toxicity detection, authorship analysis, SA, MT, QA, summarization, syntax, and semantics, and integrated them as additional query terms alongside the language

Table 1. Core rounds of the search process, including the databases searched in each round, the queries used, the publication date ranges, and the dates the searches took place

Round	Database	Query	Publication date	Search date
1st	ACL Anthology	"Modern Greek" in title or abstract	2012–2022	11/1/2022
	Scopus	TITLE-ABS({Modern Greek}) AND ALL({Natural Language Processing})	2012–2022	10/31/2022
	Semantic Scholar	Modern + Greek + Natural + Language + Processing and then "Modern Greek" in title or abstract	2012–2022	11/1/2022
2nd	ACL Anthology	"Greek" in title or abstract	2012–2022	10/24/2023
	Scopus	TITLE-ABS({Greek}) AND ALL({Natural Language Processing})	2012–2022	10/24/2023
	Semantic Scholar	Greek + Natural + Language + Processing and then "Greek" in title or abstract	2012–2022	10/24/2023
3rd	ACL Anthology	"Greek" in title or abstract	2023	7/15/2024
	Scopus	TITLE-ABS({Greek}) AND ALL({Natural Language Processing})	2023	7/15/2024
	Semantic Scholar	Greek + Natural + Language + Processing and then "Greek" in title or abstract	2023	7/15/2024

name and the overarching term "Natural Language Processing" in Google Scholar. This effort identified only five additional papers, suggesting that the original search protocol effectively captured Greek NLP publications. Therefore, we consider our approach comprehensive. Further details about this quality assurance step can be found in Note S1.

Filtering strategy

We retrieved a total of 1,717 bibliographic records, which were reduced to 1,135 after removing duplicates. Each record included metadata such as the title, author names, abstract, publication date, and citations. Publication types were manually added when missing (e.g., conference papers and journal articles). Papers not relevant to our study were discarded based on the following qualitative and quantitative exclusion criteria,

(1) Publication language: all major NLP conferences and journals publish in English; hence, studies written in other languages (including Greek) were disregarded.
(2) Language of study: with Modern Greek being the language of interest, both papers dedicated to monolingual (Greek specific) and multilingual (Greek inter alia) research were accepted; studies referring to older stages of the language (i.e., katharevousa), geographical dialects, or Greek Sign Language (GSL) were not considered.
(3) Subject area: papers irrelevant to NLP were excluded.
(4) Modality: papers not studying textual data were not considered.
(5) Publication venue: pnly conference papers and journal articles were included, leaving out book chapters, theses, and preprints.
(6) Number of citations: We applied an arithmetic progression based on both the number of citations and the year of publication, beginning with zero for papers published in 2023 and increasing with step 1 for each preceding year. In this sense, the demand for citations was higher for older publications than for more recent ones. Consequently, any paper falling below the defined citation threshold was excluded from our selection. We used Google Scholar to manually extract citation counts, due to its high coverage. This criterion ensures the inclusion of impactful and relevant papers by balancing the recency and significance of contributions, thereby streamlining the selection process.

This process resulted in a final selection of 142 papers, all published within the selected time frame. We have identified 23 additional papers that are submissions to task-specific events, such as shared tasks or workshops. Only the top-ranked submissions for each task are cited in our survey, so not all retrieved submissions are featured in the survey and are consequently excluded from the statistics.

Metadata extraction

In addition to the metadata retrieved from the databases, we gathered supplementary information to facilitate the surveying process. To ensure traceability of the retrieved papers, we recorded details about the search process, including the search date, the queried database, and the search query used. Furthermore, to aid in the filtering process, we collected information about the publication venue, as well as Google Scholar citations. After filtering and selecting the papers for review, we documented the tasks and tracks addressed by the authors, any keywords used, and the languages covered by each paper. For LRs created for each paper, we gathered information on their availability, including the URL, license, and format (for datasets). Specifically for datasets, we recorded details about their annotation type, size, linguality type (monolingual or multilingual), translation process (if applicable), domain, and time coverage.

The taxonomies

The task taxonomy

Our survey adopts a paper-driven approach to structuring the taxonomy of NLP tasks and research themes, which we propose

Table 2. Taxonomy of NLP tasks for the Greek language, organized according to the tracks of ACL 2023

Track	Task
Authorship analysis	authorship verification (3), author profiling (3), authorship attribution (2), author identification (2), author clustering (1)
Ethics and NLP	hate speech detection (6), offensive language detection (5), user content moderation (2), bullying detection (1), verbal aggression detection (1)
IE	NER (7), event extraction (3), entity linking (3), term extraction (2), open information extraction (1), web content extraction (1)
Interpretability and analysis of models for NLP	grammatical structure bias (1), word-level translation analysis in multilingual LMs, polysemy knowledge in PLMs (1), bias detection in PLMs (1)
ML for NLP	language modeling (2)
MT	MT evaluation (6), statistical machine translation (SMT) (2), rule-based MT (1)
Multilingualism and cross-lingual NLP	multilingual language learning (1), term translations detection (1), language distance detection (1), language identification (1), cross-lingual data augmentation (1), cross-lingual knowledge transfer (1)
NLP applications	legal NLP (3), business NLP (2), clinical NLP (2), educational NLP (1), media NLP (1)
QA	QA (4)
Semantics	distributional semantic modeling (4), natural language inference (2), frame semantics (2), distributional semantic models evaluation (1), lexical ambiguity (1), semantic annotation (1), semantic shift detection (1), word sense induction (1), metaphor detection (1), paraphrase detection (1), contextual interpretation (1)
SA and argument mining	document-level SA (14), sentence-level SA (13), aspect-based SA (3), argument mining (2), stance detection (1), paragraph-level SA (1)
Summarization	summarization (5), summarization evaluation (1)
Syntax and GEC	GEC (3), dependency parsing (3), POS tagging (3), sentence boundary detection (2), MWE parsing (2), tokenization (1), lemmatization (1)

Numbers in parentheses represent the count of surveyed papers that contribute to each task.

as a systematic framework for conducting monolingual NLP surveys to comprehensively capture the NLP research landscape for a specific language. This approach ensures that the selection of NLP tasks and their presentation are guided directly by the surveyed papers, allowing for a taxonomy that reflects the actual scope of research. Instead of starting with a pre-defined set of tasks, we adopt a bottom-up methodology, assigning surveyed papers to the specific NLP tasks they addressed. These tasks are then grouped into broader research themes using the comprehensive taxonomy proposed by Bommasani et al.,[70] which maps NLP tasks to thematic tracks presented at ACL 2023 edition.[71] This framework ensures that the survey aligns with contemporary research trends while systematically organizing the surveyed papers.

Canonical NLP tasks were determined based on their established tradition in NLP research, such as NER. Although we acknowledge the subjectivity in defining "canonical," we determined which tasks could be considered canonical, drawing from our expertise in the field, thereby enabling consistent organization of tasks into manageable categories. Studies addressing non-canonical tasks were categorized based on their specific focus. Subsequently, each identified task was mapped to its corresponding thematic area, as outlined by ACL 2023, enabling systematic alignment of the surveyed papers with broader NLP research themes. Table 2 illustrates the resulting taxonomy of NLP tasks for the Greek language.

In some cases, our taxonomy diverged from the ACL classification. Specifically, we present authorship analysis separately from SA and argument mining, although there is a single ACL track for "sentiment analysis, stylistic analysis, and argument mining." This decision was dictated by the fact that authorship analysis has attracted increased attention in the NLP community for Greek. Additionally, studies addressing tasks outside the scope of canonical NLP domains, such as the consolidation of historical revisions, were classified under the NLP applications track. By combining a flexible categorization strategy with a structured taxonomy, this survey comprehensively captures Greek NLP research while offering a replicable methodology for other monolingual NLP surveys.

The language resource taxonomies

One of our survey objectives was to compile a comprehensive list of the LRs developed in the reviewed studies, including detailed metadata. This metadata includes the availability of each LR ensuring it aligns with the FAIR data principles—findable, accessible, interoperable, and reusable.[72] Our search focused on the availability of URLs for each resource rather than identifying whether they were assigned persistent identifiers, such as DOIs, which may limit full compliance with the "findable" criterion. Additionally, we addressed the annotation types used for the datasets. These types, which refer to the methods employed in annotating resources, significantly affect data quality, task suitability, reproducibility, and research transparency.

Availability taxonomy. The LR availability classification scheme is based on three parameters: the presence of a functional URL, valid license information, and a machine-actionable format. We identified the resources' URLs from the papers in which they were created, without extending our search to other web sources. The scheme presented in Table 3 classifies LR availability into four distinct categories. The value "yes" signifies resources with a valid, functional URL and a defined license, such as Creative Commons. We do not evaluate license restrictions, as even restrictive licenses provide more legal clarity and alignment with FAIR principles than the absence of a license, which creates significant legal uncertainty. These datasets and lexica are also in a machine-actionable format (e.g., txt, csv, pkl). The designation "Lmt" is used for LRs with limited availability, referring to resources with valid URLs but no license terms, resources

Table 3. LR availability categories: Each category corresponds to specific criteria applied to the resource's URL, license, and data format

Availability	Description	Provided URL	License	Data format
Yes	publicly available	valid	yes (open license)	machine-actionable
Lmt	limited public availability	valid	no license or available upon request or pay	machine-actionable[a]
Err	publicly unavailable	invalid	N/A	N/A
No	no information provided	no URL	N/A	N/A

[a]For Lmt, when the LR is available upon request, the data format is unknown unless specified in the paper.

provided upon request, or accessible for a fee (e.g., tweets). Their data format is machine actionable, except for those available upon request, for which their format readiness could not be verified. The value "Err" signifies resources for which the authors provided URLs that were found to be inaccessible due to broken links or other HTTP errors. Lastly, the value "no" is assigned to resources for which the creators did not provide URLs.

Annotation type taxonomy. The classification scheme for annotation types includes six categories as outlined in Table 4. Manual annotations are performed by human annotators, offering high accuracy and often serving as the gold standard. In contrast, automatic annotations are generated using algorithms or pre-defined rules, ensuring consistency and scalability. Hybrid annotations combine both manual and automatic methods, such as performing automatic annotation followed by manual correction and validation. User-generated annotations come from real-world interactions, such as hotel review ratings from users. Curated datasets feature metadata sourced from distributors, enriching datasets with structured information such as topics from news articles or author details from publishers. Finally, "no annotation" refers to datasets that contain unprocessed text with no annotations.

TRACK: MACHINE LEARNING FOR NLP

This section marks the beginning of the discussion on track-specific research in NLP. It focuses on ML for NLP and the interpretability and analysis of models for NLP. The ML for NLP track explores how ML techniques are integrated to improve the ability of computers to understand, interpret, and generate human language. Interpretability and analysis of models for NLP is rooted in the rise of DL, which has radically changed NLP. The use of neural networks (NNs) became the dominant approach. However, their opaque nature poses challenges in understanding their inner workings, prompting a surge in research on analyzing and interpreting NN models in NLP.[73]

Table 4. LR annotation types reflecting varying levels of curation and automation

Annotation type	Description
Manual	human annotation
Automatic	automatic annotation
Hybrid	manual and automatic annotation
User-generated	annotation from user edits, not curated
Curated	metadata provided by distributor
No annotation	no annotation

Machine learning for NLP in Greek: Language models and methods

ML vs. DL approaches

ML approaches. The predominant approach to Greek NLP research in 2012 relied primarily on ML algorithms. Given the morphological richness of the Greek language, feature engineering was a key step in traditional ML. Typically, a structured pipeline was followed for extracting additional features, such as part of speech (POS) tags, lemmas, or word stems. Additionally, features such as named entities, dependency trees, and, more recently, word embeddings were often extracted. Most of the surveyed studies using an ML approach derived features from frequency-based methods, such as n-grams and lexicons (used in 41 studies), or extracted information such as POS tags, lemmas, stems, named entities, or dependency trees (used in 28 studies). Furthermore, most methods that employed word embeddings also used additional features (11 out of 15).

Regarding word embeddings, Prokopidis and Piperidis[74] trained fastText[75] on newspaper articles and the Greek part of the w2c corpus (see Greek NLP datasets: raw and multitask). Similarly, Tsakalidis et al.[76] trained Word2Vec[77] on political Greek tweets (see track: sentiment analysis and argument mining). Both sets of trained word embeddings are publicly available for research use. For the other features used in ML approaches, the corresponding tools developed by the surveyed papers are presented in various NLP track sections, according to the NLP task they address. For example, tools related to syntax are presented in track: syntax and grammar, and tools for IE, such as NER, are discussed in track: information extraction.

Early DL approaches. The adoption of RNN-based methods in Greek NLP began in 2017 with the introduction of RNN-based methods[19,78,79] and convolutional neural network (CNN)-based methods.[78,80] RNN-based methods became prevalent in Greek NLP, and when ML-based approaches were compared to RNN-based ones, the latter consistently outperformed the former.[81,82]

PLMs. PLMs following the Transformer architecture have been pivotal in recent advancements in Greek NLP. Table 5 lists the publicly available Greek PLMs developed for the studies surveyed. These models address tasks in both NLU and NLG (see Note S2). Among the monolingual PLMs designed for NLU tasks such as SA, GreekBERT[25] has emerged as a standard in Greek NLP research. It is recognized as state of the art in several studies.[23,25,83–87] GreekBERT uses the BERT-BASE-UNCASED architecture[44] and was pre-trained on 29 GB of Greek text from the Greek Wikipedia,[88] the Greek part of the European Parliament Proceedings Parallel Corpus (Europarl),[89] and the Greek part of OSCAR,[90] a clean version of Common Crawl.[91]

CellPress
OPEN ACCESS

Table 5. Monolingual Greek PLMs, including their availability and the backbone model on which they are based

Authors	Availability	Backbone
Giarelis et al.[95]	yes[97]	mT5
	yes[98]	umT5
	yes[99]	umT5
Evdaimon et al.[23]	yes[100]	BART
Koutsikakis et al.[25]	yes[101]	BERT
Zaikis et al.[92]	Lmt[102]	BERT
Alexandridis et al.[63]	Lmt[103]	BERT
Alexandridis et al.[63]	Lmt[104]	RoBERTa
Perifanos and Goutsos[83]	Lmt[105]	RoBERTa

Yes, publicly available; Lmt, limited availability (see Table 3 for details). The references cited include URLs.

There are two fine-tuned variants of GreekBERT: Greek Media BERT,[92] which is fine-tuned on media domain data, and GreekSocialBERT,[63] which is fine-tuned on Greek social media data. Additionally, PaloBERT,[63] trained on social media data, and BERTaTweetGR,[83] trained on tweets, are two monolingual models based on the RoBERTa architecture, and they also address NLU tasks. On the other hand, there are two monolingual PLMs based on the encoder-decoder architecture (see the time period of the survey), which are capable of performing all NLU and NLG tasks. GreekBART,[23] based on the BART architecture,[93] was pre-trained on the same datasets as GreekBERT plus the Greek Web Corpus,[94] incorporating diverse Greek text types, as well as formal and informal text, to enhance robustness. The GreekT5 series of models[95] was fine-tuned on the GreekSUM training dataset,[23] using the multilingual T5 LMs, which comprise google/mt5-small,[55] google/umt5-small,[96] and google/umt5-base.[96]

Interpretability and analysis of models for NLP

Research concerning interpretability and analysis of NN models for Greek NLP spans various languages and is quite diverse. Papadimitriou et al.[106] investigated grammatical structure bias in multilingual LMs, examining how higher-resource languages influence lower-resource ones. They compared Greek and Spanish monolingual BERT models with mBERT,[44] which is trained predominantly on English. The study found that mBERT tends to adopt English-like sentence structures in Spanish and Greek. They tested this phenomenon on the subject-verb order in Greek, which exhibits free word order (see the Greek language). Ahn and Oh[107] examined ethnic bias in BERT models across eight languages, including Greek, examining how these models reflect historical and social contexts. They proposed mitigation methods and highlighted the language-specific nature of ethnic bias. Garí Soler and Apidianaki[108] proposed a method to assess whether PLMs for multiple languages (including Greek) have knowledge of lexical polysemy, demonstrating their capabilities through empirical evaluation. The source code is available.[109] Gonen et al.[110] revealed the inherent understanding of mBERT for word-level translations and its capacity of cross-lingual knowledge transfer, despite the fact that it is not explicitly trained on parallel data. The source code is available.[111]

Summary of machine learning for NLP in Greek

Recently, NLP research has increasingly been based on LLMs, with some of the most popular ones being either fully or partially closed-source.[112] Notable examples for Greek include OpenAI's GPT-3.5 and GPT-4.0,[113] which are trained on multilingual data and can therefore process and generate texts in multiple languages, including Greek. Additionally, there are other multilingual PLMs available in open-source environments, such as XLM-R[49] used by Evdaimon et al.,[23] Ranasinghe and Zampieri,[24] and Koutsikakis et al.[25]; mBERT[44] used by Ahn et al.[26] and Koutsikakis et al.[25]; Flan-T5-large[114] used by Zampieri et al.[27]; and the recent GR-NLP-Toolkit.[115] New PLMs emerge regularly in multilingual and monolingual settings, such as GreekBART,[23] the GreekT5 series of models,[95] the Mistral-based Meltemi-7B,[116] and Llama-Krikri.[117] Although covering all PLMs for Greek is beyond the scope of our study, we highlight the significance of GreekBERT, which has significantly impacted Greek NLP research since its introduction in 2020, leading to a shift from traditional ML to DL approaches.

Historical evolution

Figure 2 shows the trends of Greek NLP approaches, categorized into traditional ML methods, DL methods, and other non-ML methods, such as rule-based systems. Traditional ML methods remained the dominant approach until 2019, with the exception of 2013 when other methods were favored. From 2017 onward, researchers began to use and compare both ML and DL approaches. As mentioned in machine learning for NLP in Greek: language models and methods, in 2017 the first publications employing DL techniques emerged, primarily focusing on RNN-based and CNN-based models, which accounted for approximately 30% of the total papers published that year. Since the release of GreekBERT,[25] DL methodologies have surpassed traditional ML approaches in usage. While ML methods still find applications, a significant portion of the studies employing ML techniques integrate both ML and DL techniques in their research experiments.

What we also observe in Figure 2 is a decline in research output between 2020 and 2022, particularly in studies adopting DL approaches, followed by an increase thereafter. Several factors might explain this temporary drop. First, the COVID-19 pandemic led to disruptions in research. Labs, conferences, and collaborative projects slowed down or paused during 2020–2021. Also, many researchers pivoted to pandemic-related applications of AI or public health instead of language-specific NLP. At the same period, the explosion of large-scale pre-training (BERT, GPT, and T5) heavily favored English and multilingual benchmarks like XGLUE[118] or XTREME,[119] which often provide only shallow Greek coverage. Therefore, researchers might have preferred to contribute to multilingual efforts instead of monolingual Greek projects, effectively lowering the visibility of Greek-focused work. Collectively, these elements may explain the observed short-term dip without necessarily implying long-term stagnation.

NLP approaches per track

Figure 3 illustrates the number of the surveyed papers (published from 2017 onward) across NLP tracks, categorized by their NLP approach. The starting point of 2017 reflects the emergence of DL approaches in Greek NLP, allowing for a clearer view of their integration across different tracks. We observe that ethics and

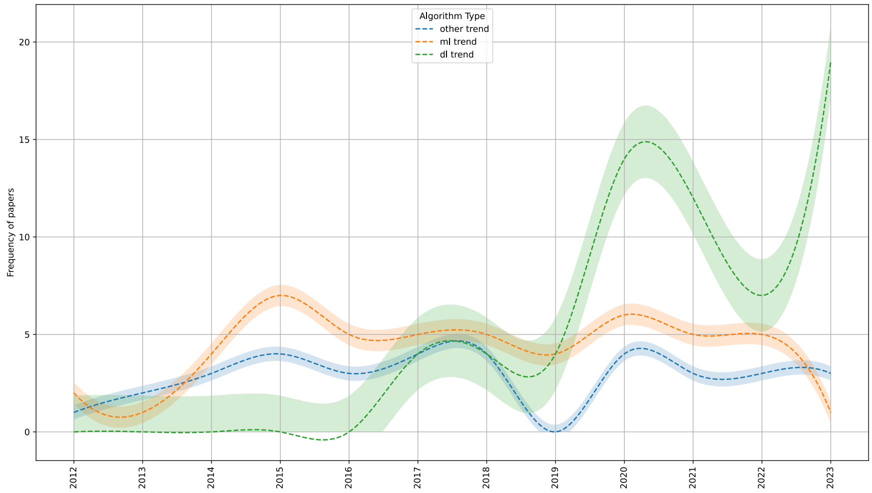

Figure 2. Frequency of NLP approaches, shown as the number of papers using each approach over the years while the shaded error band represents the standard error of the mean

The approaches include DL, traditional ML, and other methods such as rule-based systems.

NLP, IE, syntax, and summarization are predominantly addressed using DL techniques. On the other hand, QA, SA, MT, semantics, and NLP applications incorporate both traditional ML and DL approaches, either within the same study or across different studies focusing on the same task. Notably, authorship analysis is the only track where DL techniques are not employed. Additionally, ML for NLP is a recently introduced track, consisting solely of papers that adopt DL approaches.

TRACK: SYNTAX AND GRAMMAR

Syntactic processing encompasses various subtasks in NLP focused on phrase and sentence structure, as well as the relation of words and constituents to each other within a phrase or sentence.[120] It involves recognition of sentence constituents, identification of their syntactic roles, and, potentially, establishment of the underlying semantic structure. These features are valuable for NLU,[121] a topic further discussed in Note S2. Additionally, syntactic processing serves as a pre-processing step for more complex NLP tasks, such as SA and error correction among others.[122] GEC is a user-oriented task that aims for automatically correcting diverse types of errors present in a given text, encompassing violations of rules pertaining to morphology, lexicon, syntax, and semantics.[123] GEC can be used to enhance fluency, render sentences in a more natural manner, and align with the speech patterns of native speakers.[123]

Syntax and grammar in Greek: Language models and methods

Syntactic processing in Greek

This task is related to sentence splitting, tokenization, and morphosyntactic processing, including POS tagging, lemmatization, and dependency parsing. Prokopidis and Piperidis[74] addressed several syntax tasks, using the pre-trained Punkt model[124] for sentence splitting and a bidirectional long short-term memory (Bi-LSTM) tagger using the StanfordNLP library[125] for POS tagging. Lemmatization involved a lexicon-based approach with a Bi-LSTM lemmatization model as a fallback for out-of-lexicon words. For dependency parsing, the authors trained a neural attention-based parser[126] on the Greek Universal Dependencies (UD) treebank.[127] On the same dataset, Koutsikakis et al.[25] performed POS tagging using Transformer-based models, namely their GreekBERT, XLM-R, and two variants of mBERT, concluding that all four have comparable performance in terms of accuracy. Partalidou et al.[128] conducted POS tagging and NER tasks, with the details of their NER system summarized in track: information extraction. For POS tagging they used spaCy,[129] adhering to the UD annotation schema. Additionally, they assessed the model's tolerance toward out-of-vocabulary (OOV) words and found that it lacked flexibility in handling such instances. Widely used NLP pipelines in the surveyed papers are an ILSP suite of NLP tools,[130] the Natural Language Toolkit (NLTK),[131] polyglot,[132] spaCy for Greek,[133,134] Stanza,[135] and UDPipe.[136] Additional research in the field of syntax explored hybrid embeddings proposed by Zuhra and Saleem[137] to enhance dependency parsing for morphologically rich, free-word-order languages, including Greek, using UD treebanks. These hybrid embeddings were based on POS tags and morphological features, significantly improving parsing accuracy. Wong et al.[138] developed a multilingual sentence boundary detection method based on an incremental decision tree learning algorithm. Furthermore, while Fotopoulou and Giouli[139] and Samaridi and Markantonatou[140] dealt with verbal multiword expressions (MWEs), the former study aimed at defining formal criteria for classifying verbal MWEs as either idiomatic expressions or support verb constructions (consisting of a support verb and a predicative noun). In contrast, the latter focused on parsing MWEs using the Lexical-Functional Grammar/Xerox Linguistic Environments (LFG/XLEs) framework, extending their analysis beyond traditional syntactic boundaries by incorporating lexical knowledge from lexicons.

GEC in Greek

Korre et al.[141] focused on the correction of grammatical errors that vary from grammatical mistakes to punctuation, spelling, and morphology of words. The authors listed 18 main categories of grammatical errors that systems can correct, also developing a rule-based annotation tool for Greek. The tool takes an original erroneous sentence along with its correction as input. It then automatically produces an annotation that mainly consists of the error location and type as well as its correction. Gakis et al.[142] created a rule-based grammar checker tool,[143] which analyzes and corrects syntactic, grammatical, and stylistic (i.e., the formal, informal, or oral style of language used) errors in sentences, providing users with error notifications and correction hints. Kavros and Tzitzikas[144] focused on spelling errors, addressing the issue of misspelled and mispronounced words in Greek. They employed phonetic algorithms to assign the same code to different word variations based on phonetic rules. For

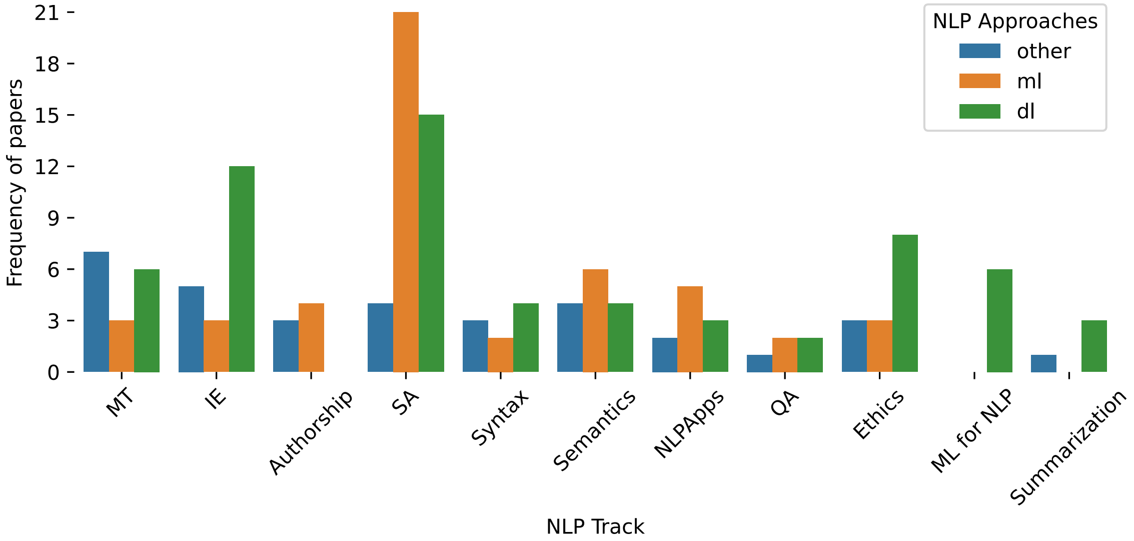

Figure 3. Number of papers per NLP track per approach (ML, DL, and other) since 2017, the year when a study could follow multiple approaches (e.g., both ML and DL)

example, they successfully grouped "μήνυμα" (correct spelling) with "μύνημα" (both sounding as /mínima/). They reported better results compared to stemming and edit-distance approaches. The source code is available.[145]

Syntax and grammar in Greek: Language resources

Table 6 displays the pertinent monolingual LRs for this track. It shows three publicly available resources for GEC and two resources for syntax, of which one is publicly available. For GEC, Kavros and Tzitzikas[144] created word lists, containing words and their misspellings. These misspellings were generated through the addition, deletion, or substitution of a letter, as well as by incorporating words with similar sounds. Korre et al.[141] developed two datasets, namely the Greek Native Corpus (GNC) and the Greek Wiki Edits (GWE). GNC comprises essays written by students who are native speakers of Greek, totaling 227 sentences. Each sentence within this dataset may contain zero, one, or multiple grammatical errors, all annotated with the corresponding grammatical error types as defined in the provided annotation schema. On the other hand, GWE consists of sentences extracted from WikiConv.[146] Each sentence in this dataset includes the original sentence, the edited sentence, the original string that underwent editing, and the specific grammatical error type.

Regarding syntax, Prokopidis and Papageorgiou[127] provided the Greek UD treebank as part of the UD project,[150] a project that offers standardized treebanks with consistent annotations across languages. The dataset includes syntactic dependencies, POS tags, morphological features, and lemmas. Derived from the Greek Dependency Treebank,[151] it contains 2,521 sentences split into training (1,622), development (403), and test (456) sets, and was manually validated and corrected. Gakis et al.[149] collected a corpus consisting of 2.05M tokens derived from student essays, literary works, and newspaper articles. They extracted morphosyntactic information automatically for this corpus with the help of a lexicon.[152]

Summary of syntax and grammar in Greek

Traditionally, syntactic processing served as a pre-processing step for higher-level NLP tasks (track: machine learning for NLP). However, in the era of DL-based NLP, syntactic processing is often neglected. Instead, NNs are leveraged to implicitly capture syntactic information, surpassing the performance of symbolic methods that rely on manually hand-crafted features. This is also reflected by the number of ACL submissions related to syntax (i.e., tagging, chunking, and parsing), which is significantly shrinking.[28] Our study partially reflects this trend, showing a slight decline in focus on syntactic tasks since 2020, though they remain active. Notably, the syntax and Grammar track, alongside the IE track (see track: information extraction), has the highest number of publicly available LRs for Greek and the largest proportion of publicly available LRs among all task-related LRs.

TRACK: SEMANTICS

The meaning in language is the focus of semantics. In the context of NLP, semantic analysis aims to extract, represent, and interpret meaning from textual data, bridging the gap between natural language and machine understanding.[153] Semantic analysis can operate at three different levels, each focusing on different units of examination: lexical semantics, sentence-level semantics, and discourse analysis. Lexical semantics pertains to the understanding of word meanings, including their various senses, relationships with other words, and roles in different linguistic contexts.[154] Sentence-level semantics considers the meaning of individual sentences or phrases in terms of their internal structure and relationships. Discourse analysis, on the other hand, deals with understanding the meaning in a broader textual context, beyond individual sentences.[155] It involves analyzing how sentences connect and influence each other within the context of a text or a conversation.

At the core of lexical semantics lies the task of distributional semantics, which is the leading approach to lexical meaning

Table 6. LRs related to GEC and syntax, with information on availability, annotation type, size, size unit, and text type

Authors	Availability	Ann. type	Size	Size unit	Text type
Kavros and Tzitzikas[144]	yes[145]	automatic	1,086	word	word list
Korre et al.[141]	yes[147]	manual	227	sentence	student essay
	yes[147]	user-generated	100	sentence	Wikipedia talk page
Prokopidis and Papageorgiou[127]	yes[148]	hybrid	2,521	sentence	Wikinews, European parliament sessions
Gakis et al.[149]	no	automatic	2.05M	token	essay, literature, news

Yes, publicly available; no, no information provided (see Table 3 for details). For annotation type, see Table 4 for details. The references cited include URLs.

representation in NLP.[156] Founded upon the distributional hypothesis,[157,158] which suggests that words sharing similar linguistic contexts also share similar meanings, distributional semantics employs real-valued vectors, commonly known as embeddings, to encode the linguistic distribution of lexical items within textual corpora. As Lenci et al.[156] explain, this field has progressed through three key generations of models: (1) count-based distributional semantic models (DSMs), which form distributional vectors based on co-occurrence frequencies that adhere to the bag of words (BoW) assumption; (2) prediction-based DSMs, employing shallow NNs to learn vectors by predicting adjacent words, yielding dense, static word embeddings (or simply word embeddings); and (3) contextual DSMs, harnessing deep neural language models to generate inherently contextualized vectors for each word token (e.g., word embeddings extracted from BERT-based models). The evolution from earlier static DSMs, which learn a single vector per word type, to contextual DSMs is further examined in the time period of the survey.

Semantics in Greek: Language models and methods

Lexical semantics

Studies focusing on lexical semantics in Greek address the following tasks: building DSMs, DSM evaluation, diachronic semantic shifts of words, word sense induction, lexical ambiguity, metaphor detection, and semantic annotation.

Zervanou et al.[159] used BoW representations to study the impact of morphology on unstructured count-based DSMs. They proposed a selective stemming process, by using a metric to determine which words to stem, demonstrating improved performance in morphologically rich languages such as Greek. Palogiannidi et al.[160,161] used semantic similarity and BoW representations of seed words to estimate the ratings of unknown words, applying their method on affective lexica of five different languages, including Greek. Iosif et al.[162] proposed word embeddings inspired by cognitive processes in human memory,[163] showing that they outperform BoW representations. Lioudakis et al.[164] introduced the continuous bag of skip-gram (CBOS) method for generating word representations, combining continuous skip-gram with continuous bag of words (CBOW), and assessing its performance across various tasks (e.g., word analogies and word similarity). The source code is available.[165]

Outsios et al.[166] performed evaluation of various word embeddings trained on diverse data sources. The evaluation framework considered tasks involving word analogies and similarity. Dritsa et al.[167] and Barzokas et al.[168] investigated the diachronic semantic shifts of words with the use of distributional semantics. Dritsa et al.[167] constructed a dataset from Greek Parliament proceedings (further discussed in Greek NLP datasets: raw and multitask). They also applied four state-of-the-art semantic shift detection algorithms, namely Orthogonal Procrustes,[169] Compass,[170] NN,[171] and Second-Order Similarity,[172] to identify word-usage change across time and among political parties. Barzokas et al.[168] compiled a corpus of e-books (presented in Greek NLP datasets: raw and multitask), trained word embeddings, and used k-nearest neighbors along with cosine distances to trace semantic shifts aiming to capture both linguistic and cultural evolution.

Garí Soler and Apidianaki[108] introduced an approach to analyze lexical polysemy knowledge in PLMs across various languages, including Greek. They found that contextual LM representations, like BERT, encode information about lexical polysemy, and they performed word sense induction by enabling interpretable clustering of polysemous words based on their senses. On the other hand, Gakis et al.[149] analyzed lexical ambiguity using morphosyntactic features from a lexicon.[152] They categorized ambiguous words based on their spelling and etymology. Florou et al.[173] focused on metaphor detection using the discriminative model of Steen,[174] identifying the literal and metaphorical functions of phrases through the optimal separation of hyperplanes in vector representations of word combinations.

Chowdhury et al.[175] addressed the challenge of transferring semantic annotations from a source language corpus (Italian) to a target language (Greek) using crowdsourcing. They introduced a methodology to evaluate the quality of crowd-annotated corpora by considering inter-annotator agreement for evaluation of annotations within the target language, whereas cross-language transfer quality is evaluated by comparison against source-language annotations.

Sentence-level semantics

We identified two tasks in Greek NLP that fall under sentence-level semantics: semantic parsing and natural language inference (NLI). Semantic parsing involves converting natural language utterances into logical forms that can be executed on a knowledge base.[176] Li et al.[177] tackled this task using synchronous context-free grammars (SCFGs), which model language relationships by deriving coherent logical forms. They enhanced the SCFG framework by extending the translation rules with informative symbols, achieving state-of-the-art performance in English, Greek, and German on a benchmark dataset. In contrast, NLI focuses on assessing the logical relationship between sentence pairs, determining whether one sentence entails, contradicts, or is neutral with respect to another.

Table 7. LRs related to semantics, with information on availability, annotation type, linguality type, size, and size unit (with size denoting the portion in Greek for multilingual datasets)

Authors	Availability	Ann. type	Size	Size unit	Linguality
Ganitkevitch and Callison-Burch[181]	yes[182]	hybrid	22.3M	paraphrase	multilingual
Garí Soler and Apidianaki[108]	Lmt[109]	automatic	418	word	multilingual
Outsios et al.[166]	Err[183]	manual	353	word-pair	monolingual
	Err[184]	automatic	39,174	word analogy question	monolingual
Pilitsidou and Giouli[185]	no	manual	73,069	token	bilingual
Giouli et al.[186]	no	manual	3,012	token	monolingual
Florou et al.[173]	no	manual	914	sentence	monolingual

Yes, publicly available; Lmt, limited availability; Err, unavailable; no, no information provided (see Table 3 for details). For annotation type, see Table 4 for details. The references cited include URLs.

Koutsikakis et al.[25] evaluated this task using the Greek part of the XNLI corpus,[178] comparing their model GreekBERT with XLM-R, two variants of mBERT, and the decomposable attention model (DAM).[179] They found that GreekBERT outperformed the other models. Three years later, Evdaimon et al.[23] fine-tuned their model, GreekBART, on the XNLI training split and compared it with GreekBERT and XLM-R on the test split, concluding that GreekBART achieved results comparable to GreekBERT.

Discourse analysis

The only study identified that performs discourse analysis is by Giachos et al.[180] This study focused on how the robot processes and understands sentences in context, teaching the robot to handle incomplete information and enabling a word-learning procedure, beginning with 200 Greek words as a seed dictionary.

Semantics in Greek: Language resources

Table 7 presents the LRs for semantics-related tasks, along with their availability (classified according to Table 3), annotation type (classified as per Table 4), linguality type, size, and size unit. By contrast to syntax and grammar (track: syntax and grammar), only one LR regarding semantics is publicly available. The remaining six are either of limited availability (Lmt), could not be accessed (Err), or were not publicly available (no).

The only publicly available resource is that of Ganitkevitch and Callison-Burch,[181] who expanded the Paraphrase Database (PPDB)[187] with paraphrases in 23 languages, including Greek. The original database contains human-annotated paraphrases in English. For the additional languages, Ganitkevitch and Callison-Burch[181] extracted paraphrases using parallel corpora. This study was not mentioned earlier in this section because there were no other contributions except for the introduction of this LR. Garí Soler and Apidianaki[108] offered a multilingual dataset comprising words, their corresponding senses, and sentences featuring the word in its specific sense. In the case of the Greek part of the corpus, sentences were extracted from the Eurosense corpus,[188] which contains texts from Europarl, automatically annotated with BabelNet word senses.[189] Outsios et al.[166] translated to Greek the benchmark dataset WordSim353,[190] which contains word pairs along with human-assigned similarity judgments. Additionally, they assembled 39,174 analogy questions to conduct word analogy tests, measuring word similarity in a low-dimensional embedding space.[191] LRs of the three studies that were not publicly available were about the Greek counterpart of the Global FrameNet project,[186] a bilingual frame-semantic lexicon for the financial domain,[185] and a corpus that consists of sentences using the same transitive verbs in both metaphorical and literal contexts.[173]

Summary of semantics in Greek

Studies pertaining to semantics can be found throughout the period under investigation (2012–2023), but most were published in early years (2013–2016; see Figure 4). Most of the studies focus on lexical semantics. While various semantics-related tasks are addressed, typically only one study per task is observed. An exception regards DSM, where significant attention has been directed toward prediction-based methods, with notable studies being those of Iosif et al.[162] and Lioudakis et al.,[164] who proposed new approaches to generate word embeddings, and of Outsios et al.[166] who undertook a word-embedding benchmark. We also acknowledge that contextual embeddings, which comprise rich information,[192,193] are heavily understudied in Greek, despite the existence of publicly available models.[23,25] An exception is the work of Garí Soler and Apidianaki,[108] who investigated the potential of contextual embeddings to capture lexical polysemy.

TRACK: INFORMATION EXTRACTION

IE concerns the automated identification and extraction of structured data, including entities, relationships, events, or other factual information from unstructured text. The primary objective of IE is to make the information machine readable, facilitating analysis, search, and practical use of textual information.[194]

Information extraction in Greek: Language models and methods

Below, we present studies addressing IE (in descending order of recency) and NER.

IE

Mouratidis et al.[195] conducted a study on extracting maritime terms from legal texts in the Official Government Gazette of the Hellenic Republic. They identified these terms by counting token lengths, setting a threshold, and using lexicon-based stemmed tokens from maritime dictionaries introduced in their previous

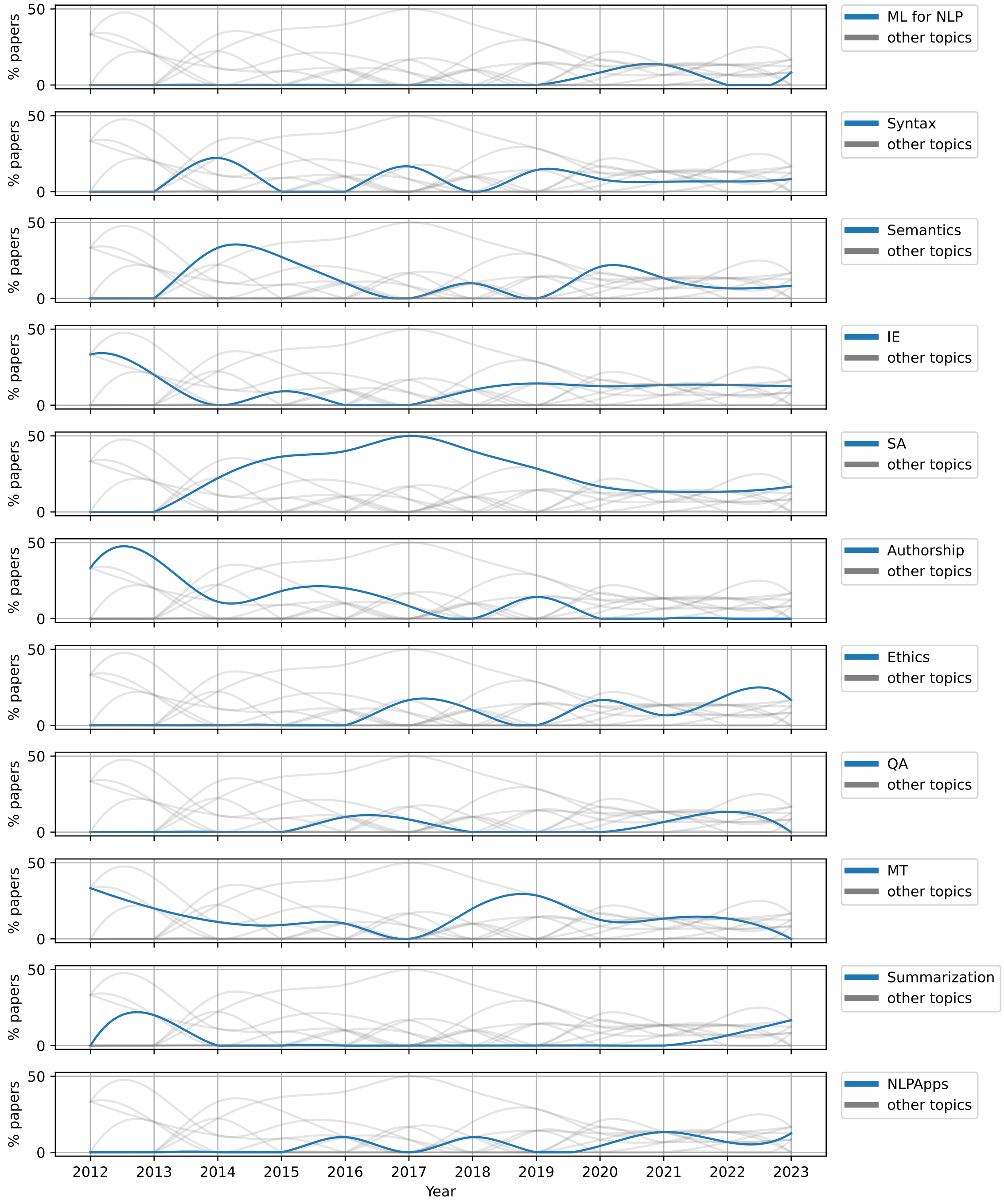

Figure 4. Relative popularity of tracks over time

Each time series illustrates the relative percentage of studies per year for a specific track (bold blue line) with time series of other tracks appearing shadowed in the background.

study.[196] Additionally, they derived word embeddings and used them to train RNN-based models, incorporating the maritime term extraction features into the training process. In a separate study, Papadopoulos et al.[20] tackled Open Information Extraction (Open IE), a process that involves converting unstructured text into <SUBJECT; RELATION; OBJECT> tuples. Addressing the challenge of Open IE in languages that are resource-lean for this task, such as Greek, Papadopoulos et al.[20] used neural machine translation (NMT) between English and Greek to generate English translations of Greek text (track: machine translation, multilingualism, and cross-lingual NLP). These were then processed through an NLP pipeline,[197] enabling co-reference resolution, summarization, and triple extraction using existing English LMs and tools, then back-translating the extracted triplets to Greek. Barbaresi and Lejeune[198] evaluated web content extraction tools on HTML 4 standard pages in five different languages

(Greek, Chinese, English, Polish, and Russian), concluding that the three best tools for Greek perform comparably to the three top tools for English; for the rest of the languages the results are much lower than in English and Greek. Finally, Lejeune et al.[199] developed a multilingual (Chinese, English, Greek, Polish, and Russian) rule- and character-based event extraction system, where an event is defined minimally as a pair consisting of a disease and its corresponding location. This system was also referenced in prior studies of the authors.[200,201]

NER

This task involves the identification and categorization of specific entities, such as names of people, organizations, locations, dates, and more, within unstructured text. Papantoniou et al.[202] conducted NER and entity linking on a dataset derived from Greek Wikipedia event pages. They assessed five established methods for NER and four methods for entity linking, including three designed for English, which required translating Greek text into English. Rizou et al.[203] carried out NER and intent classification tasks on queries from a university helpdesk dataset with Greek and English submissions. They employed joint-task methods using Transformer-based models. In their earlier work, Rizou et al.[85] applied the same tasks with the same methods to the widely used English benchmark dataset, the Airline Travel Information System (ATIS),[204] which they also translated into Greek. Bartziokas et al.[205] curated NER datasets and evaluated five deep neural network (DNN) models on them, selected for their high performance on the English CoNLL-2003[206] and OntoNotes 5[207] datasets, showing performance comparable to that in English. Koutsikakis et al.[25] performed NER using their model, GreekBERT, as well as XLM-R and two variants of mBERT, finding that GreekBERT outperformed the other three LMs in terms of micro-F1. Partalidou et al.[128] employed spaCy[129] for POS tagging and NER, discovering limited impact of POS tags on NER. Angelidis et al.[208] performed NER and entity linking in legal texts. For NER they used long short-term memory (LSTM) models; for entity linking, Levenshtein and substring distance were evaluated; for entity representation and linking, a resource description framework (RDF) specification was chosen. In entity linking, Papantoniou et al.[209] performed NER on the text, generating candidates for the extracted entities from several wiki-based knowledge bases before conducting disambiguation.

Information extraction in Greek: Language resources

Table 8 presents the LRs developed for IE tasks in Greek. Four out of the nine LRs were publicly available, three of which were about news.

By focusing on LRs related to named entities, Papantoniou et al.[202] created a dataset from the Greek Wikipedia Events pages by automatically annotating eight entity tags. The annotation was performed by identifying terms that appeared in Wikidata, which also facilitated entity linking. Rizou et al.[203] created a dataset of graduate student questions to two Greek universities, requesting the students to provide their questions in both Greek and English. The dataset is manually annotated with three entity tags and six intents. Bartziokas et al.[205] provided two annotated datasets, one with four label tags akin to the CONLL-2003 dataset[206] and the other incorporating 18 tags for entities, as in the OntoNotes 5 English dataset.[207] These datasets were developed during the GSOC2018 project (discussed in track: syntax and grammar), where the initial automatic annotation was followed by manual curation. Lioudakis et al.[164] converted the GSOC2018 named entity annotated dataset to the CONLL-2003 format. The source dataset was annotated using Prodigy,[217] whereby the initial annotations were done manually; subsequently, model predictions were used to accelerate the annotation process. Rizou et al.[85] undertook the task of translating to Greek the ATIS corpus dataset,[204] eliminating duplicate entries. The dataset consists of audio recordings and manual transcripts of inquiries related to flight information in automated airline travel systems. It is complemented by annotations for named entities within the airline travel domain and intent categories. Angelidis et al.[208] curated a dataset containing 254 daily issues of the Greek Government Gazette spanning the period 2000–2017, manually annotated for six entity types. Lejeune et al.[201] offered 1,681 documents in five languages, annotating them regarding diseases and locations where applicable. Mouratidis et al.[196] conducted stemming on legal texts related to maritime topics from the Official Government Gazette of the Hellenic Republic, annotating tokens as either maritime terms or not.

Summary of information extraction in Greek

IE studies in Greek primarily focus on NER, often accompanied by datasets. Of the nine reported LRs, four are publicly available, while another four could become available in the future, as their links are provided but currently result in HTTP errors. Figure 4 shows that there was relative interest in IE early on (2012–2014), which was discontinued (up to 2017) and then kept an upward trend. This can explain the tendency toward DL approaches in this track, as highlighted in Figure 3, which is probably related to efforts to create benchmark datasets.[85,205,208] Such benchmark datasets create the resources needed to train and assess DL models. Another notable study in light of the data scarcity in certain IE tasks is the work of Papadopoulos et al.,[20] who leveraged cross-lingual transfer-learning techniques.

TRACK: SENTIMENT ANALYSIS AND ARGUMENT MINING

The SA task concerns the detection of opinions expressed in opinionated texts, while argument mining concerns the detection of the reasons why people hold their opinions.[218]

As its name suggests, SA involves the analysis of human sentiments toward specific entities. In addition to the analysis of sentiment, the task also concerns opinions, appraisals, attitudes, or emotions,[219] while the entities discussed can be products, services, organizations, individuals, events, issues, or topics, among others. It is particularly active in domains such as finance, tourism, health, and social media, SA involves applications in recommendation-based systems,[220] business intelligence,[221] and predictive or trend analyses.[222,223] The field of SA has evolved significantly since it was popularized by the pioneering work of Turney[224] and Pang et al.,[225] who classified texts as positive or negative. Subsequent studies have expanded and enriched the field, moving beyond binary classification and introducing slightly different tasks and alternative terms such as opinion mining, opinion analysis, opinion extraction, sentiment mining, subjectivity analysis, affect analysis, emotion analysis,

Table 8. Datasets related to IE with information on availability, annotation type, size, size unit, and domain

Authors	Availability	Ann. type	Size	Size unit	Domain
Papantoniou et al.[202]	yes[210]	automatic	474,361	token	news
Rizou et al.[203]	yes[211]	manual	4,302	sentence	university
Bartziokas et al.[205]	yes[212]	hybrid	623,700	token	news
	yes[212]	hybrid	623,700	token	news
Rizou et al.[85]	Err[213]	manual	5,473	sentence	airline travel
Lioudakis et al.[164]	Err[214]	hybrid	N/A	N/A	N/A
Angelidis et al.[208]	Err[215]	manual	254	piece	legal
Lejeune et al.[201]	Err[216]	manual	390	document	epidemics
Mouratidis et al.[196]	no	manual	80,000	word	maritime law

Yes, publicly available; Err, unavailable; no, no information provided (see Table 3 for details). For annotation type, see Table 4 for details. The references cited include URLs.

and review mining, all of which now fall under the umbrella of SA.[219] Further information on this track and background knowledge are included in Note S3.

Sentiment analysis and argument mining in Greek: Language models and methods

Document-level SA

Evdaimon et al.[23] evaluated their GreekBART model, along with GreekBERT[25] and XLM-R[49] LMs, on a user-annotated movie reviews dataset—the Athinorama_movies_dataset[226]—for binary SA, and found that GreekBERT outperformed the other models. Additionally, Bilianos[86] used GreekBERT[25] to classify the polarity of product reviews as positive or negative, while Braoudaki et al.[227] conducted binary polarity classification of hotel reviews, experimenting with LSTM architectures and lexicon-based input features. Medrouk and Pappa[80,228] applied binary polarity classification as well but experimented with monolingual and multilingual input. Multilingual SA was addressed also by Manias et al.,[229] who investigated the impact of NMT on SA. The authors translated part of the English IMDb reviews dataset[230] to Greek and German and trained the same NN architecture on SA using either the source or the target language as input. Translation was also used by Athanasiou and Maragoudakis[19] for data-augmentation purposes.

Early document-level SA approaches were mainly based on ML and feature engineering, employing information such as term frequency,[231] POS,[232–236] and sentiment lexicons (see Table 10). Features crafted from sentiment lexicons have been found beneficial compared to dense word embeddings because the latter do not carry sentiment information,[237] while Markopoulos et al.[231] noted that the TF-IDF representation outperformed lexicon-based features. Feature-engineering-based SA is not optimal compared to DL counterparts.[63,86,87,234,236] Spatiotis et al.[234,236] applied feature engineering for SA in hybrid educational systems, using features such as school level, region, and gender.

Sentence-level SA

Zaikis et al.[92] created a unified media analysis framework that classifies sentiment, emotion, irony, and hate speech in sentence- and paragraph-level texts by using a joint learning approach. This method leveraged the similarities between these tasks to enhance overall performance. Patsiouras et al.[238] classified political tweets across four dimensions: sentiment polarity (three-class), figurativeness (ironic, sarcastic, figurative, or literal), aggressiveness (offensive, abusive, racist, or neutral language), and bias (strongly opinionated or not). They employed a CNN and a Transformer-based architecture for classification, using data augmentation techniques to handle imbalanced categories. Both Katika et al.[239] and Kapoteli et al.[87] fine-tuned GreekBERT for binary sentiment classification of COVID-19-related tweets, with the former focusing on long-COVID effects and the latter on COVID-19 vaccination. Alexandridis et al.[63] performed a benchmark of SA methods by either including the neutral class or not. In the binary setting, the GPT2-Greek[240] LM outperformed ML methods that used GreekBERT and FastText word embeddings. In the three-class setting, only DL methods were used. The authors created and shared two PLMs, PaloBERT[104] and GreekSocialBERT,[103] with the latter outperforming the former, which in turn outperformed GreekBERT. In a subsequent study, Alexandridis et al.[84] compared their LMs in emotion detection and concluded that GreekBERT consistently exhibited better performance than PaloBERT. Drakopoulos et al.[241] used graph neural networks (GNNs) on tweets, which were found to provide more accurate estimations of intentions by aggregating information about the Twitter account.

In earlier work on sentence-level SA, Tsakalidis et al.[76] highlighted the importance of considering the domain in SA, noting that n-gram representations are more effective for intra-domain SA, while word embeddings and lexicon-based methods are more suitable for cross-domain SA. Charalampakis et al.[242,243] ranked the features they used in descending order of significance, based on information gain. Solakidis et al.[244] conducted semi-supervised SA and emotion detection using lexicon-based n-gram features of emoticons and keywords and found that emoticons can intensify and indicate the presence and polarity of specific sentiments within a document. Chatzakou et al.[245] categorized social media input into 12 emotions using lexicon-based features of sentiment words and emoticons, whereby they translated from Greek the words of the input texts to English for the usage of English sentiment lexicons. Besides ML-based SA, there are also studies exploring sentiment in real-world situations, such as COVID-19[246] and pre-election events.[235]

Aspect-based SA

Antonakaki et al.[247,248] analyzed political discourse on Twitter by conducting entity-based SA and sarcasm detection. They manually identified entities and performed lexicon-based SA at the entity level. For sarcasm detection, they trained a support vector machine (SVM) algorithm using lexicon-based sentiment features and topics extracted through topic modeling, based on the hypothesis that certain topics are more closely associated with sarcasm. The source code of is available.[249] Petasis et al.[250] performed entity-based SA to support a real-world reputation management application, monitoring whether entities are perceived positively or negatively on the web.

Stance detection

Tsakalidis et al.[251] aimed to nowcast on a daily basis the voting stance of Twitter users during the pre-electoral period of the 2015 Greek bailout referendum. They performed semi-supervised, time-sensitive classification of tweets, leveraging text and network information.

Argument mining

Sliwa et al.[252] tackled argument mining for non-English languages using parallel data. They used parallel data pairs with English as the source language and either Arabic or a Balkan language (including Greek) as the target language. They automatically annotated English sentences for argumentation using eight classifiers and extended the labels to the target languages using majority voting. Sardianos et al.[253] identified segments representing argument elements (i.e., claims and premises) in online texts (e.g., news) by using conditional random fields (CRFs)[254] and features based on POS tags, cue word lists, and word embeddings.

Sentiment analysis and argument mining in Greek: Language resources

Table 9 presents the datasets related to SA and argument mining, along with information on their availability (see Table 3), annotation type (see Table 4), size, size unit, and classes of annotation. Besides datasets, LRs in Greek comprise sentiment lexicons, which are summarized in Table 10 and have been used, or could have been used, to extract features for ML algorithms.[255]

Document-level SA

Document-level datasets in Greek mainly regard product reviews. Bilianos[86] presented 240 negative and 240 positive electronic product reviews. These reviews consist of user-generated content with ratings adjusted by the researchers to generate binary polarity. The remaining studies focusing on document-level SA created non-publicly available datasets annotated either for emotion[87] or sentiment.[19,80,227–229,231–234,236,250]

Sentence-level SA

Datasets annotated for sentiment at the sentence-level in Greek primarily consist of tweets. Patsiouras et al.[238] created, and provide upon request, a dataset of 2,578 unique tweets manually annotated across four different dimensions: sentiment polarity (three-class), figurativeness (ironic, sarcastic, figurative, or literal), aggressiveness (offensive, abusive, racist, or neutral language), and bias (strongly opinionated or not). The tweets span the period from March 2014 to March 2021. Chatzakou et al.[245] presented a dataset of randomly selected tweets annotated for 12 emotions through crowdsourcing and majority voting. Kydros et al.[246] created, and provide upon request, a dataset of tweets related to the COVID-19 pandemic. These tweets were automatically annotated using lexicons to determine their sentiment as either positive or negative, with an additional annotation for anxiety. Antonakaki et al.[247,248] presented three datasets of tweets focused on politics. Two datasets feature automatic sentiment annotation on a scale from −5 to 5, while the third is manually annotated for sarcasm using crowdsourcing, with a binary classification. Although all datasets include the full tweet texts and are released under the Apache 2.0 license,[265] this conflicts with X's terms of service and copyright law.[266] As a result, they are classified as having limited availability according to the availability schema (see availability taxonomy). Tsakalidis et al.[76] offered a tweet dataset related to the January 2015 General Elections in Greece annotated for positive or negative sentiment as well as a second dataset of election-related tweets annotated for sarcasm. Both datasets were filtered to include only instances where annotators agreed. Makrynioti and Vassalos[260] randomly sampled 8,888 tweets from August 2012 to January 2015 and annotated them as positive, negative, or neutral. Charalampakis et al.[242,243] shared two tweet datasets annotated for irony. Each dataset includes 162 manually annotated tweets, with the rest of the tweets having been automatically annotated. These tweets were collected in the weeks before and after the May 2012 parliamentary elections in Greece and are characterized by the political parties and their leaders. The remaining studies focusing on sentence-level SA are not publicly available and primarily use tweets[87,235,239,241,244,251] or social media content from other sources.[63] Exceptions include Giatsoglou et al.,[237] who segmented user reviews on mobile phones into sentences for annotation, and Zaikis et al.,[92] who collected data from the internet, social media, and press, annotating it at both the sentence and paragraph levels. Additionally, all datasets are annotated for sentiment, except in the cases of Solakidis et al.,[244] who annotated both sentiment and four emotions, and Zaikis et al.,[92] who annotated for sentiment, irony, hate speech, and emotions.

Argument mining

Sliwa et al.[252] provided a collection of bilingual datasets containing sentences labeled as argumentative or non-argumentative, available upon request. The sentences were automatically annotated by eight different argument mining models, and the final label was determined based on the majority vote of these models. These datasets were derived from parallel corpora where the source language is English and the target language is either a Balkan language or Arabic. Additionally, Sardianos et al.[253] made a dataset available upon request. This dataset consists of 300 news articles from the Greek newspaper *Avgi*,[267] annotated by two human annotators (150 articles each) for argument components, i.e., premises and claims.

SA lexicons

Tsakalidis et al.[76] developed three lexicons with data collected between August 1, 2015 and November 1, 2015. The first lexicon, the Greek Affect and Sentiment lexicon (GrAFS), was derived from the digital version of the Dictionary of Standard Modern Greek,[268] which was web-crawled to gather words used in an ironic, derogatory, abusive, mocking, or vulgar manner. This process yielded 2,324 words (later reduced to 2,260 after editing) along with their definitions. These words

Table 9. Datasets for SA and argument mining, indicating their availability status, annotation type, size, size unit, and sentiment annotation classes

Authors	Availability	Ann. type	Size	Size unit	Class
Patsiouras et al.[238]	Lmt[256]	manual	2,578	tweet	(positive, negative, neutral), (figurative, normal), (aggressive, normal), (partisan, neutral)
Bilianos[86]	Lmt[257]	user-generated	480	review	positive, negative
Kydros et al.[246]	Lmt	automatic	44,639	tweet	positive, negative, anxiety
Sliwa et al.[252]	Lmt	automatic	166,430	sentence	argumentative, non-argumentative
Tsakalidis et al.[76]	Lmt	manual	1,640	tweet	positive, negative, neutral
	Lmt	manual	2,506	tweet	sarcastic, non-sarcastic
Chatzakou et al.[245]	Lmt[258]	manual	2,246	tweet	Ekman's six basic emotions and enthusiasm, rejection, shame, anxiety, calm, interest
Antonakaki et al.[247,248]	Lmt[259]	automatic	301,000	tweet	−5 to −1 (negative), 1 to 5 (positive)
	Lmt[259]	automatic	182,000	tweet	−5 to −1 (negative), 1 to 5 (positive)
	Lmt[259]	manual	4,644	tweet	sarcastic, non-sarcastic
Makrynioti and Vassalos[260]	Lmt	manual	8,888	tweet	positive, negative, neutral
Sardianos et al.[253]	Lmt	manual	300	document	argument
Charalampakis et al.[242]	Err[261]	hybrid	44,438	tweet	ironic, non-ironic
Charalampakis et al.[243]	Err[261]	hybrid	61,427	tweet	ironic, non-ironic
Katika et al.[239]	no	hybrid	937	tweet	positive, negative, neutral
Zaikis et al.[92]	no	manual	14,579	sentence, paragraph	(positive, negative, neutral), (ironic, not ironic), (hate, not hate), (happiness, contempt, anger, disgust, surprise, sadness, none)
Alexandridis et al.[84]	no	manual	3,875	tweet	Ekman's six basic emotions, anticipation, trust, none
	no	manual	54,916	document	positive, negative, neutral
Alexandridis et al.[63]	no	manual	59,810	social media text	positive, negative, neutral
Kapoteli et al.[87]	no	manual	1,424	tweet	positive, negative, neutral
Braoudaki et al.[227]	no	user-generated	156,700	review	positive, negative
Drakopoulos et al.[241]	no	automatic	17.465M	tweet	positive, negative
Spatiotis et al.[232,234,236]	no	manual	11,156	review	very positive, positive, neutral, negative, very negative
Manias et al.[229]	no	user-generated	4,251	review	positive, negative, unsupported
Beleveslis et al.[235]	no	automatic	46,705	tweet	positive, negative, neutral
Medrouk and Pappa[228]	no	user-generated	91,816 (EL, EN, FR)	review	positive, negative
Tsakalidis et al.[251]	no	hybrid	1.64M	tweet	favor, against
Medrouk and Pappa[80]	no	user-generated	2,600	review	positive, negative
	no	user-generated	7,200 (EL, EN, FR)	review	positive, negative
Athanasiou and Maragoudakis[19]	no	manual	740	comment	positive, negative
Giatsoglou et al.[237]	no	manual	2,800	sentence	positive, negative
Spatiotis et al.[233]	no	manual	11,156	review	very positive, positive, neutral, negative, very negative
Markopoulos et al.[231]	no	manual	1,800	review	positive, negative
Petasis et al.[250]	no	manual	2,300	text	positive, negative
Solakidis et al.[244]	no	hybrid	25,700	tweet	positive, negative, neutral, joy, love, anger, sadness

Lmt, limited availability; Err, unavailable; no, no information provided (see Table 3 for details). For annotation type, see Table 4 for details. The references cited include URLs.

Table 10. Sentiment lexica with information on availability, size, size unit, and sentiment annotation classes

Authors	Availability	Size	Size unit	Class
Tsakalidis et al.[76]	Lmt[262]	2,260	word	subjectivity, polarity, and Ekman's six basic emotions
	Lmt[262]	190,667	n-gram	positive, negative
	Lmt[262]	32,980	n-gram	Ekman's six basic emotions
Giatsoglou et al.[237]	Err[263]	4,658	word	subjectivity, polarity, and Ekman's six basic emotions
Palogiannidi et al.[161]	Err[264]	407,000	word	valence, arousal, dominance
Antonakaki et al.[247]	no	4,915	word	positive, negative
Markopoulos et al.[231]	no	68,748	token	positive, negative

Lmt, limited availability; Err, unavailable; no, no information provided (see Table 3 for details). The references cited include URLs.

were manually annotated as objective, strongly subjective, or weakly subjective. Subjective words were further annotated as positive, negative, or both, and each annotation was rated on a scale from 1 (least) to 5 (most) based on Ekman's six basic emotions. The annotations were then automatically extended to all inflected forms, resulting in 32,884 unique entries. To capture informalities prevalent in Twitter content, the authors also developed two Twitter-specific lexicons collecting tweets using seed words from the first lexicon: the keyword-based lexicon (KBL) with 190,667 n-grams and the emoticon-based lexicon (EBL) with 32,980 n-grams. Giatsoglou et al.[237] proposed an expansion of the lexicon of Tsakalidis et al.,[269] which included 2,315 words annotated for subjectivity, polarity, and Ekman's six emotions. This expanded lexicon incorporated synonyms grouped around each term and assigned a vector containing the average emotion over all dimensions and terms, resulting in a total of 4,658 Greek terms. Palogiannidi et al.[161] introduced an affective lexicon of 1,034 words with human ratings for valence, arousal, and dominance, originating from Bradley and Lang.[270] The terms were translated, manually annotated by multiple annotators, and automatically expanded using a semantic model to estimate the semantic similarity between two words, resulting in a final lexicon of 407,000 words. The following lexicons are not publicly available. Antonakaki et al.[247] presented a lexicon consisting of 4,915 words manually annotated for polarity. This lexicon is a compilation of three independent lexicons: two general-purpose lexicons and one from the political domain. Markopoulos et al.[231] developed a sentiment lexicon of Greek words from a corpus they constructed, covering terms with positive or negative meanings. The lexicon includes all inflected forms of the words, resulting in 68,748 unique entries.

Summary of sentiment analysis and argument mining in Greek

SA for Greek is applied to hotel and product reviews, political posts, educational questionnaires, COVID-19-related posts, and trending topics discussed on Twitter, listed here in descending order of frequency. We have also observed studies that deal with a range of distinct emotions or a specific emotion, e.g., anxiety or irony, which is a sentiment that is extremely challenging to capture in NLP.[271] The SA task has attracted significant attention in the field of NLP for Greek (Figure 4), constituting approximately one-fourth of the studies (23.4%). This attention reached its peak in 2017 before experiencing a slight decrease. A similar trend is observed across ACL Anthology tracks, albeit slightly earlier, i.e., between 2013 and 2016.[28] Despite the abundance of studies, however, we observe that a publicly available Greek dataset that can serve as an SA benchmark does not exist. Even among the published datasets limitations exist, such as missing licenses or paywalls (the datasets marked as "Lmt" in the availability-type column) or unavailability due to HTTP errors (the datasets marked as "Err" in the availability-type column). Furthermore, studies exploring SA through lexicons have generated new ones, yet none of these are publicly accessible.

TRACK: AUTHORSHIP ANALYSIS

Authorship analysis attempts to infer information about the authorship of a piece of work.[272] It encompasses three primary tasks: author profiling, detecting sociolinguistic attributes of authors from their text; authorship verification, determining whether a text belongs to a specific author; and authorship attribution, pinpointing the right author of a particular text from a predefined set of potential authors.[272] Both authorship verification and authorship attribution are variations of the broader author identification problem, which seeks to determine the author of a text.[273] Another pertinent task within authorship analysis is author clustering, which entails grouping documents authored by the same individual into clusters, with each cluster representing a distinct author.[274] Although other tasks may relate to authorship analysis, research on Greek authorship analysis has predominantly focused on these five tasks; therefore, we concentrate on them.

Authorship analysis: Language models and methods

In Greek, authorship analysis has been supported by a workshop series addressing various tasks within this field. Furthermore, additional studies concentrating on the fundamental tasks of authorship analysis have been identified.

PAN

A workshop series and a networking initiative, called PAN,[275] has been dedicated to the fields of digital text forensics and stylometry since 2007. Its objective is to foster collaboration among researchers and practitioners, exploring text analysis in terms of authorship, originality, trustworthiness, and ethics, among others. PAN has organized shared tasks focusing on computational

Table 11. Datasets related to authorship analysis with information on availability, annotation type, size, size unit, and domain

Authors	Availability	Ann. type	Size	Size unit	Domain
Stamatatos et al.[280]	Lmt[294]	manual	330	document	articles, reviews
Stamatatos et al.[279]	Lmt[295]	manual	393	document	news
Stamatatos et al.[278]	Lmt[296]	manual	385	document	news
Juola and Stamatatos[277]	Lmt[297]	manual	120	article	news
Halvani et al.[286]	Lmt[298]	curated	190	recipe, article	cooking, various
Juola et al.[281]	no	manual	200	essay	personal & academic topics
Mikros[289]	no	curated	479,439	word	science, society, economy, art
Mikros[291]	no	curated	406,460	word	blog

Lmt, limited availability; no, no information provided. References cited are linkable except for "no"; see Table 3 for details. For annotation type, see Table 4 for details.

challenges related to authorship analysis, computational ethics, and plagiarism detection, amassing a total of 64 shared tasks with 55 datasets provided by the organizing committees and an additional nine contributed by the community.[276] Among these shared tasks, four specifically addressed Greek, with three dealing with author identification[277–279] and one with author clustering.[280]

Authorship attribution

Juola et al.[281] benchmarked an attribution framework, JGAAP,[282] on a corpus of Greek texts that were authored by students who also translated their texts to English. Authorship attribution was substantially more accurate in English than in Greek. They provided three possible reasons suitable for future investigation. First, the framework or the features tested excel in English (selection bias). Second, authorship pool bias may exist due to the authors' non-native English proficiency, potentially affecting the error rate. Third, linguistically, Greek may possess inherent complexities that hinder individual feature extraction.

Authorship verification

This task has been addressed in Greek in a multilingual setting. Kocher and Savoy[283] suggested an unsupervised baseline by concatenating the candidate author's texts and comparing the 200 most frequently occurring terms (words and punctuation symbols) extracted from these texts with those extracted from the disputed text. Hürlimann et al.[284] trained a binary linear classifier on top of engineered features (e.g., character n-grams, text similarity, and visual text attributes); the source code is available.[285] Halvani et al.[286] approached the task with a single-class classification, demonstrating strong performance in the PAN-2020 competition.[287,288]

Author profiling

Mikros[289] and Perifanos[290] performed gender identification in Greek tweets. They deployed ML algorithms using stylometric features at the character and word levels, most frequent words in the text, and gender-related keywords lists extracted from the texts. Specifically, Mikros[289] focused on stylometric features, including lexical and sublexical units, to analyze their distribution in texts written by male and female authors. His study concluded that men and women use most stylometric features differently. In a prior study, Mikros[291] conducted author gender identification and authorship attribution using stylometric features, employing the sequential minimal optimization (SMO) algorithm.[292] His findings indicated that author gender is conveyed through distinctive syntactical and morphological patterns, whereas authorship is linked to the over- or under-representation of certain high-frequency words.

Authorship analysis in Greek: Language resources

Table 11 displays the LRs corresponding to authorship analysis tasks in Greek. Some datasets have limited availability due to the lack of a provided license, while others are not available at all. Most of the limited-availability LRs originate from the PAN workshop series. Although our method returned the task overview papers from the PAN-2013-2014-2016 workshops, we include all Greek datasets from the PAN workshop series for comprehensive coverage. Juola and Stamatatos[277] delivered a corpus for the PAN-2013 author identification task, which contains documents in Greek, English, and Spanish. The Greek part of the corpus comprises newspaper articles published in the Greek weekly newspaper *To Vima*[293] from 1996 to 2012. The authors organized the Greek corpus into 50 verification task groups, where each group comprises documents with known authors and one document with an unknown author. The grouping criteria included genre, theme, writing date, and stylistic relationships. Stamatatos et al.[278] provided a larger corpus for the PAN-2014 author identification task, consisting of 200 verification task groups of documents and including Dutch in addition to the previously mentioned languages. Unlike PAN-2013, no stylistic analysis was conducted on the texts to identify authors with very similar styles or texts by the same author, with notable differences. The PAN-2015 corpus for the author identification task[279] is the same size as that in PAN-2014 and includes the same languages, but it allows for cross-topic and cross-genre author verification without assuming that all documents share the same genre or topic. Stamatatos et al.[280] introduced a dataset for the PAN-2016 author clustering task, including documents in Greek, English, and Dutch, spanning two genres (articles and reviews) and covering various topics. The Greek part of the corpus comprises six document groups specifically created for author clustering and authorship-link ranking.

Additionally, Halvani et al.[286] provided the test part of their dataset, which includes 120 recipes and 70 news articles, but it is also not accompanied by a license. The other three LRs are not publicly available. Juola et al.[281] performed authorship attribution on Greek and English student essays, which were written by 100 students following specific instructions. In contrast, Mikros[289,291] focused on identifying gender in news texts and blogs,

creating datasets that are equally balanced by gender and topic, with this information provided by the distributors.

Summary of authorship analysis in Greek

Authorship analysis studies in Greek address all three primary tasks: authorship attribution, verification, and profiling. A significant contribution to this field is the inclusion of Greek benchmark datasets in the PAN workshop series (from PAN-2013 to PAN-2016 workshops), which focus on digital text forensics and stylometry. The availability of Greek datasets through this workshop series has been pivotal for advancing authorship analysis research in Greek, with many early studies using these datasets. However, this initial boost was not sustained after 2016, rendering this track less popular than others (Figure 4). The most recent study retrieved dates back to 2019, with the majority of studies concentrated between 2012 and 2017. Recent global advancements in authorship analysis tasks, such as DL and transfer learning,[299] have not yet been adopted for Greek. Additionally, the evolution in the field has introduced new tasks, such as bots profiling, author obfuscation, and style change detection, areas where no relevant studies have been conducted in the Greek context.[300]

TRACK: ETHICS AND NLP

Within the thematic domain of ethics and NLP, a wide range of topics is addressed globally, encompassing issues such as overgeneralization, dual use of NLP technologies, privacy protection, bias in NLP models, under-representation, fairness, and toxicity detection, among others.[301] Studies pertaining to Greek within this domain primarily focus on toxicity detection, i.e., the automated identification of abusive, offensive, or otherwise harmful user-generated content (more details about definitions are provided in Note S4). This task is a necessity for online content moderation, including social media moderation, content filtering, and prevention of online harassment, all aimed at fostering a safer and more respectful online environment.[302] A notable exception among the many toxicity detection studies in Greek is the work of Ahn and Oh,[107] which explores ethnic bias detection in PLMs (discussed in track: machine learning for NLP).

Toxicity detection has sparked heated debates. Criticism primarily focuses on datasets and the way researchers approach the problem, often oversimplifying the issue and disregarding the variety of use cases.[303] Toxicity can be interpreted differently depending on factors such as social group membership, social status, and privilege, leading to unequal impacts on marginalized communities.[304] A second point of criticism highlights the fact that commonly used datasets, on which the models are trained and evaluated, often lack sufficient context to allow reliable judgments.[305,306] Lastly, the definition of toxicity is subjective and dependent on the annotator's perspective.[307,308] Recent studies indicate that annotators may not simply disagree but can be polarized in their assessments regarding the toxicity of the same text. For instance, a text can be found toxic by all women annotators but considered benign by all men.[309] However, such factors are rarely incorporated into research tasks, which adversely affects how the task is framed and how the automated systems are developed. Furthermore, differences in the interpretation of toxicity are evident in the datasets and models that are created.[307,308]

Ethics and NLP in Greek: Language models and methods

Toxicity detection: Multilingual shared task

In 2020, a subtask of offensive language identification for Greek was introduced as part of the SemEval-2020 Task 12 on Multilingual Offensive Language Identification in Social Media (OffensEval-2020).[310] The task focused on four other languages besides Greek: Danish, English, Turkish, and Arabic. Overall, 145 teams submitted official runs on the test data, 37 of which made an official submission on Greek, while the submissions for English were approximately double, with 81 submissions. The three top systems[26,311,312] primarily relied on BERT-based LMs, with the first two using multilingual models and the third a monolingual one.

Toxicity detection: Greek shared task subtask

The Greek dataset used for the OffensEval-2020 subtask is an extended version of the Offensive Greek Tweet Dataset (OGTD), which was developed by Pitenis et al.[81] In their original article, the authors trained ML classifiers on extracted features, including TF-IDF unigrams, bigrams, POS, and dependency relation tags and obtained their best results from a DL network, by feeding word embeddings[94] into LSTM and gated recurrent unit (GRU) cells equipped with self-attention mechanisms.[313] Three years after the release of the OffensEval-2020 subtask, Zampieri et al.[27] evaluated LLMs on OffensEval-2020 datasets and from the previous OffensEval-2019,[314] which focused exclusively on English. They used the top three systems of each language track as baselines. While eight LLMs were evaluated on the English datasets, only the Flan-T5-large LLM,[114] which is fine-tuned in other languages besides English, was tested on the other languages, including Greek. In all non-English languages, the macro F1 score of the LLM demonstrated a significant improvement from the third-place system. Additionally, they reviewed recent popular benchmark competitions on the topic, none of which, apart from OffensEval, included Greek. Both Zaikis et al.[92] and Ranasinghe and Zampieri[24] used the extended version of OGTD to evaluate their systems. The former developed a unified media-analysis framework designed to classify sentiment, emotion, irony, and hate speech in texts through a joint learning approach (see track: sentiment analysis and argument mining). They evaluated their system on this dataset and also tested their media domain fine-tuned version of GreekBERT, the Greek Media BERT.[102] The latter employed cross-lingual contextual word embeddings and transfer-learning techniques to adapt the XLM-R[49] model, initially trained on English offensive language data (OLID),[315] for detecting offensive language in Greek and six other low-resource languages.

Toxicity detection by companies

Toxicity detection in online content has also been explored in the Greek context in industry-led or industry-supported efforts. For instance, considering both fully and semi-automatic user content moderation in the comments section of the Gazzetta sports portal,[316] Pavlopoulos et al.[78] equipped a GRU-based RNN with a self-attention mechanism for toxicity detection. Their results suggested that an ablated version, using only self-attention and disregarding the RNN, performs considerably well given its simplicity. In a different study of the same data, Pavlopoulos

Table 12. Datasets designed for toxicity-detection-related tasks, with information on availability, annotation type, size, size unit, and detection class

Authors	Availability	Ann. type	Size	Size unit	Class
Zampieri et al.[310]	yes[323]	manual	10,287	tweet	offense
Perifanos and Goutsos[83]	Lmt[324]	manual	4,004	tweet	toxicity
Pontiki et al.[318]	Lmt[325]	automatic	4.490M	tweet	xenophobia
Pavlopoulos et al.[78]	Lmt[326]	manual	1.450M	comment	toxicity
Arcila-Calderón et al.[82]	Err[327]	manual	15,761	tweet	racism, xenophobia
Nikiforos et al.[322]	no	manual	583	sentence	bullying
Lekea and Karampelas[321]	no	automatic[a]	81	manifesto	terrorism

Yes, publicly available; Lmt, limited availability; Err, unavailable; no, no information provided (see Table 3 for details). For annotation type, see Table 4 for details. The references cited include URLs.
[a]Unclear annotation process.

et al.[79] showed that user embeddings lead to improved performance.

Hate speech

Research focusing on the phenomenon of hate speech targeting specific groups has garnered significant interest in Greek. Greece, serving as a primary entry point for a large number of immigrants arriving in Europe, has witnessed the emergence of xenophobic and racist opinions directed toward immigrants and refugees.[317] Arcila-Calderón et al.[82] tackled racist and/or xenophobic hate speech detection on tweets in Greek, Spanish, and Italian using BERT-based LMs. The same task was addressed by Perifanos and Goutsos,[83] also for Greek but with a multimodal approach, to account for hateful content that does not necessarily carry textual streams, i.e., images. For the text modality they used GreekBERT, and they also created the BERTaTweetGR LM (described in track: machine learning for NLP), while, for joint representations of text and tweet images, they used a single model that combines the representations of BERT and ResNet. Pontiki et al.[318] performed verbal aggression analysis on Twitter data. They identified different aspects of verbal aggression related to pre-defined targets of interest. Patsiouras et al.[238] classified political tweets into four different dimensions, one of which was aggressive language (further details of which are provided in track: sentiment analysis and argument mining). Kotsakis et al.[319] developed and evaluated a web framework[320] that was designed for automated data collection, hate speech detection, and content management of multilingual content from social media targeting refugees and migrants. The evaluation was conducted by human experts who assessed hate speech detection using both a lexicon-based approach and an RNN-based approach. Lekea and Karampelas[321] detected hate speech within terrorist manifestos, classifying these manifestos into three categories: no hate speech, moderate hate speech, and evident hate speech. Finally, Nikiforos et al.[322] detected bullying within virtual learning communities—online communities created for educational purposes—and applied linguistic analysis to recognize behavior patterns.

Ethics and NLP in Greek: Language resources

Table 12 presents the relevant LRs in Greek, along with their availability (as shown in Table 3), annotation type (detailed in Table 4), size, and the type of content targeted for detection. Only one dataset is publicly available, licensed, and accessible at the time of this study. Half of the datasets are publicly available but have licensing issues, another one was inaccessible, and two others are not publicly available (both with very few data).

Pitenis et al.[81] introduced a dataset comprising 1,401 offensive and 3,378 non-offensive tweets, sourced from popular and trending hashtags in Greece, keyword queries containing sensitive or obscene language, and tweets featuring /eisai/ (“you are”) as a keyword. Zampieri et al.[310] extended this dataset for OffensEval-2020, increasing the total to 10,287 tweets. The extension involved manual annotation by three annotators, with the final label determined by majority voting among the annotators.

Perifanos and Goutsos[83] curated 1,040 racist and/or xenophobic hate speech tweets and 2,964 non-hate-speech tweets, providing both their tweet IDs and code for retrieving them. Their dataset reflects an overlap of neo-Nazi, far-right, and alt-right social media accounts systematically targeting refugees, LGBTQ activists, feminists, and human rights advocates. The dataset was annotated by three human rights activists. The final label for each tweet was determined by majority voting among the annotators. Pontiki et al.[318] gathered a dataset of 4,490,572 tweets exhibiting verbal attacks against ten pre-defined target groups during the financial crisis in Greece. The dataset has limited availability, as it does not include the tweets or tweet IDs. Pavlopoulos et al.[78] offered 1.6 million manually moderated user-generated encrypted comments from Gazzetta,[316] a Greek sports news portal. Arcila-Calderón et al.[82] provided a multilingual dataset in Greek, Italian, and Spanish, containing records of racist/xenophobic hate speech from various sources such as news articles, Twitter, YouTube, and Facebook. Nikiforos et al.[322] developed a manually annotated corpus on bullying within VLCs using conversations from Wikispaces. The corpus was annotated by two annotators, but the dataset was not publicly released. Finally, Lekea and Karampelas[321] annotated manifestos authored by members of the terrorist organization 17 November with hate speech tags (moderate/apparent/no hate speech). However, they did not publicly release these annotated documents, and details of the annotation process were not clearly provided in their publication.

Summary of ethics and NLP in Greek

Studies on ethics and NLP in the context of Greek language are predominantly focused on toxicity detection rather than

addressing issues related to responsible AI, data ethics, or privacy preservation, which were covered in the second ACL Workshop on Ethics in NLP.[328] Toxicity detection in Greek has been facilitated within the setting of the OffensEval multilingual shared task. The Greek subtask attracted approximately half the submissions compared to its English counterpart. Additional studies on toxicity detection include collaborations with private companies and efforts specifically targeting hate speech against certain groups. An analysis of the toxicity detection methods and LRs available for Greek reveals that published studies are more concerned with social issues pertinent to Greek society[62,82,83,321] rather than improving existing methods or sharing new LRs. With a few exceptions focusing on hate speech detection from a social perspective,[321,322] the predominant approach in this domain relies heavily on DL techniques. Notably, shared LRs for Greek often lack conversational context, aligning with a global trend observed in toxicity detection research.[305] Furthermore, studies predominantly target social groups responsible for generating hate speech, overlooking hate speech originating from marginalized groups, who may use it as a form of self-defense or as a way to assert their rights.

TRACK: SUMMARIZATION

Summarization is the task of automatically generating concise and coherent summaries from longer texts while preserving key information and overall meaning.[329] It aims to distill the most important points, ideas, or arguments from a document or multiple documents into a shorter version, typically a paragraph or a few sentences.[330] Summarization in NLP research began back to 1958 with Luhn's work,[331] which automatically excerpted abstracts of magazine articles and technical papers. There are generally two main types of summarization: extractive summarization, i.e., extracting the most important sentences or phrases directly from the original text and then combining them to form a summary,[332] and abstractive summarization, i.e., generating new sentences that convey the essential information from the original text in a more concise manner. This method may involve paraphrasing and rephrasing content using NLG techniques, such as reinforcement learning (RL) approaches and sequence-to-sequence Transformer architectures.[333] In Greek, the most common language-generation approaches use sequence-to-sequence Transformer architectures.

Summarization in Greek: Language models and methods

For the Greek language, we identified extractive summarization techniques implemented in shared task series and a workshop, as well as two PLMs used to create abstractive summaries. Additionally, another study also investigated both types of summaries in the legal domain.

Extractive summarization

The Financial Narrative Summarization Shared Task (FNS) has been part of the Financial Narrative Processing (FNP) workshop series since 2020. It involves generating either abstractive or extractive summaries from financial annual reports. Initially focusing on English, it expanded to include Greek and Spanish in 2022.[334] In FNS-2023,[335] six systems from three participating teams competed, with extractive summarization being predominant among the top three systems.[336,337] The winning system[336] employed an algorithm[338] that allocates words across narrative sections based on their weights, combining and summarizing the content. The second and third best systems[337] performed summarization by first filtering out noisy content, either by retaining the first 10% of the text or using the BertSum summarizer[339] to select 3,000-word segments and then applying a positional LM,[340] which incorporates positional information to understand the order of words in a sequence. In FNS-2022,[334] 14 systems from seven teams competed, with most addressing Greek and Spanish.[334] The top-performing systems for Greek were all from one team,[338] including the system that later won in FNS-2023, and all performed extractive summarization. These systems combined narrative sections either by ascending page order or descending weight order to generate summaries. Giannakopoulos[341] provided an overview of the MultiLing 2013 Workshop at ACL 2013, which introduced a multilingual, multidocument summarization challenge. This challenge assessed summarization systems across ten languages, including Greek. It featured two tasks: generating summaries that describe document sets as event sequences and creating systems to evaluate these summaries. Among the seven participants, three submitted results for Greek.

Abstractive summarization

For Greek, there are two monolingual PLMs based on sequence-to-sequence Transformer architectures. Evdaimon et al.[23] developed GreekBART, a BART-based model[93] pre-trained on a corpus that included the same datasets as GreekBERT (see track: machine learning for NLP), and the Greek Web Corpus dataset.[94] Giarelis et al.[95] fine-tuned the multilingual T5 architecture LMs (google/mt5-small,[55] google/umt5-small,[96] and google/umt5-base[96]), which follow a sequence-to-sequence approach, on the GreekSUM train-split dataset,[23] creating the GreekT5 series of models. They evaluated both GreekT5 and GreekBART on the GreekSUM Abstract test-split dataset,[23] reporting that GreekT5 outperformed GreekBART in ROUGE metrics while GreekBART excelled in the BERTScore metric. The evaluation code is available.[342]

Extractive and abstractive summarization

Ket al.[343] addressed the challenge of summarizing Greek legal documents through both abstractive and extractive methods, using both automatic and human metrics for evaluation. They employed LexRank[344] and Biased LexRank[345] for extractive summarization, while for abstractive summarization they used a sequence-to-sequence approach with a BERT-based encoder-decoder architecture, initializing both components with GreekBERT weights.

Summarization in Greek: Language resources

Table 13 presents the pertinent LRs for summarization. We observe that one is publicly available, while the rest lack a license.

Koniaris et al.[343] created a legal corpus of 8,395 court decisions from Areios Pagos,[352] the Supreme Civil and Criminal Court of Greece. This corpus includes the decisions, their summaries, and related metadata, all sourced from the Areios Pagos website. The dataset is divided into training, validation, and testing sets. Evdaimon et al.[23] collected articles from a news website on various topics to create two summarization datasets.

Table 13. LRs for summarization, with information on availability, annotation type, size and size unit of the summarized documents, and text domain

Authors	Availability	Ann. type	Size	Size unit	Domain
Koniaris et al.[343]	yes[346]	curated	8,395	document	legal
Evdaimon et al.[23]	Lmt[347]	curated	151,000	document	news
Zavitsanos et al.[335]	Lmt[348]	manual	312	document	finance
El-Haj et al.[334]	Lmt[349]	manual	262	document	finance
Li et al.[350]	Lmt[351]	manual	1,350[a]	document	news

Yes, publicly available; Lmt, limited availability (see Table 3 for details). For annotation type, see Table 4 for details. The references cited include URLs.
[a]This is the size of the multilingual dataset, not just the Greek portion.

In the first dataset, the titles serve as the summaries, while in the second, the abstracts are used. Both datasets are also divided into training, validation, and testing sets. However, these datasets are currently not accompanied by a license. Zavitsanos et al.[335] and El-Haj et al.[334] supplied the Greek datasets for the FNS-2023 and FNS-2022 shared subtasks, respectively. The first dataset included 312 financial reports, while the second included 262 financial reports. In both datasets, each report ranged from 100 to 300 pages and was accompanied by at least two human-generated gold-standard summaries.

Li et al.[350] provided the corpora for the MultiLing 2013 Workshop at ACL 2013,[341] based on a subset of English content from WikiNews.[353] This English content was manually translated into nine languages, including Greek, resulting in nine parallel corpora. The English-Greek parallel corpus included ten topics, each with a human-generated summary serving as the gold standard.

Summary of summarization in Greek

Summarization in Greek has gained notable attention recently, mainly due to its inclusion in shared tasks (FNS 2022–2023) and the introduction of two monolingual encoder-decoder PLMs that perform NLG tasks. While the shared tasks were fostering both extractive and abstractive summarization, the top systems performed extractive summarization. In contrast, sequence-to-sequence Transformer architectures, specifically BART[93] and T5,[54] were implemented to support abstractive summarization for Greek in a monolingual setting. Recent research, building on these shared tasks and PLMs, has significantly advanced the field by introducing four new datasets specifically designed for summarization, compared to only one dataset available before 2022.

TRACK: QUESTION ANSWERING

QA aims to automatically answer user questions in natural language.[354] There are several real-world applications linked to QA, ranging from decision and customer support systems[355,356] to chatbots[357] and personal assistants.[358] Given a question such as "which is the birthplace of Plato?," a QA system is expected to predict the correct answer: "Athens." However, QA can be very broad in terms of the types of the questions that can be asked.[359] For example, a statement such as "my name is John," which is not a question, could receive an answer of the form "that is a nice name" or "nice to meet you, John."

Table 14. Dialog with ChatGPT to illustrate the generative approach whereby a model generates answers without relying on a pre-defined knowledge base

Question	Answer
What might be the future impact of widespread AI integration in daily life?	in a future with ubiquitous AI, we could experience personalized and efficient services, enhanced decision-making support, and seamless automation across various aspects of daily life, transforming the way we work and interact with technology

The QA format

The QA format can take multiple variations. For instance, it can extract the answer from a given knowledge base, whether structured (e.g., documents or database) or not, or it can generate the text of the answer without any such support, as is the case with ChatGPT.[360] An example of QA through prompting ChatGPT is shown in Table 14.

These systems can further be classified based on various criteria, such as the nature of the questions and the types of answers they require (e.g., factoid [refers to simple factual questions] vs. non-factoid). Additionally, classification can be based on the breadth of domain coverage and the sources of knowledge employed to generate answers (e.g., closed domain vs. open domain).[361]

QA evaluation

A QA system is usually trained and evaluated on an appropriate dataset, tackling tasks such as NLU, information retrieval (IR), reasoning, and world modeling.[362–364] These evaluations serve to measure the system's performance across various aspects of language understanding and response generation.

Question answering in Greek: Language models and methods

Existing QA systems for Greek use extractive algorithms that extract answers from given knowledge bases. Mountantonakis et al.[21] introduced an open-domain, factoid cross-lingual QA system. Specifically, they translated user questions from Greek to English and used English BERT-based QA models to retrieve answers from DBpedia abstracts.[365] The answers were then translated back into Greek before being returned to the user. Additionally, the context from which answers are retrieved, known as the knowledge base, may also be structured data. An example of this is the closed-domain, factoid QA system by Marakakis et al.,[366] which converted the user's question into a database query, searched the database, and combined the noun and verb phrases of the question with the response to form an answer in natural language.

Off-the-shelf

Services have been used to build QA systems providing conversational interfaces even without the need for programming

CellPress
OPEN ACCESS

knowledge.[367] Specifically, Malamas et al.[368] and Ventoura et al.[369] developed e-healthcare assistants, with the latter providing information and support specifically for the COVID-19 pandemic. Both studies used an NLU service, Rasa,[370] in order to generate the answer, which first extracted the intent (e.g., pharmacy finder) and the entities (e.g., "city" and "time") from the question (e.g., "what pharmacies will be open in Athens tomorrow morning at 8?").

Question answering in Greek: Language resources

Table 15 presents the only two Greek QA LRs developed in the studies reviewed. Both resources are monolingual and have limited accessibility. First, Mountantonakis et al.[21] provided an evaluation dataset for QA systems, comprising 20 texts on diverse subjects, derived from a Greek text bank.[371] For each of these 20 texts, they crafted ten questions along with their respective correct answers, resulting in a total of 200 questions and answers. Lopes et al.[372] offered a dataset of audio recordings and the corresponding transcriptions of 200 dialogs collected from call-center interactions regarding movie inquiries. This dataset, which is available upon request, is also manually annotated with gender, task success, anger, satisfaction, and miscommunication annotations, making it suitable for a range of NLP tasks beyond QA.

Summary of question answering in Greek

Reflecting on the available QA LRs and architectures for Greek, several key observations arise. First, there is a clear scarcity of QA resources specifically designed for Greek. To confirm this, we conducted a search in the Hugging Face repository for Greek QA datasets, which revealed 20 datasets containing Greek text. Of these, 18 were multilingual, which makes reliance on such resources essential for advancing Greek QA tasks. Moreover, these datasets are predominantly translations from English. However, a limitation of these multilingual, translated datasets is that they do not offer representative samples of Greek questions. This is consistent with the observation by Rogers et al.,[362] who noted that multilingual QA benchmarks often prioritize uniformity across languages, potentially overlooking natural or representative language-specific samples. Additionally, the development of QA architectures for Greek remains relatively underexplored, with only a handful of studies addressing this area. Notably, no DL-based QA solution has been proposed specifically for Greek, which may be due to the lack of dedicated monolingual QA LRs for the language.

TRACK: MACHINE TRANSLATION, MULTILINGUALISM, AND CROSS-LINGUAL NLP

This section concerns studies handling multiple languages in NLP, including Greek. The most canonical task in this thematic domain is MT, i.e., the automated translation from one natural language to another. Emerging as one of the earliest tasks a computer could possibly solve, MT was inspired by Warren Weaver's "translation memorandum" in 1947 and IBM's word-for-word translation system in 1954.[374] For Greek, most work focuses on evaluating MT, using MT for data augmentation, or enabling NLP tools in other languages through MT.

Table 15. QA datasets, indicating availability, annotation type, size, and size unit

Authors	Availability	Ann. type	Size	Size unit
Mountantonakis et al.[21]	Lmt[373]	manual	200	question-answer pair
Lopes et al.[372]	Lmt	manual	200	dialog

Yes, publicly available; Lmt, limited availability (see Table 3 for details). For annotation type, see Table 4 for details. The references cited include URLs.

Machine translation, multilingualism, and cross-lingual NLP in Greek: Language models and methods

Machine translation

Kouremenos et al.[375] developed a rule-based MT system between Greek and GSL for producing parallel corpora of Greek and GSL glossed text. In this context, glossing is a method for representing the meaning and grammatical structure of signed language in written form. The authors opted for a rule-based approach due to the absence of a standardized writing system for GSL, the scarcity of publicly available GSL grammar resources, and the lack of Greek-GSL parallel data. Beinborn et al.[376] focused on the automatic production of cognates using character-based MT, applying their method on language pairs with different alphabets, specifically from English to Greek, Russian, and Farsi. Their aim was to identify not only genetic cognates, meaning two words in two languages that have the same etymological origin,[377] but also words that are sufficiently similar to be associated by language learners. For example, the English word "strange" has the Italian correspondent "strano." The two words have different roots and are therefore genetically unrelated. For language learners, however, the similarity is more evident than, for example, the English-Italian genetic cognate father-padre. Their approach relied on phrase-based SMT using the MOSES framework,[378] but instead of translating phrases, they transformed character sequences from one language into the other, using words instead of sentences and characters instead of words. Pecina et al.[379] aimed to adapt a general-domain SMT system to specific domains by acquiring in-domain monolingual and parallel data through domain-focused web crawling. The authors specifically focused on two language pairs, i.e., English-Greek and English-French, and two domains, i.e., natural environment and labor legislation.

Studies evaluating MT systems have consistently demonstrated the superior performance of NMT compared to SMT. Mouratidis et al.[380] tackled MT evaluation specifically for the English-Greek and English-Italian language pairs. They developed a DL framework that integrates an RNN with linguistic features, word embeddings, and automatic MT metrics. The evaluation used small, noisy datasets consisting of educational video subtitles. In an earlier study, Stasimioti et al.[381] also included human evaluation, considering factors such as post-editing effort, adequacy and fluency ratings, and error classification. Previous studies of the same team retrieved by our search protocol dealing with MT evaluation were those by Mouratidis et al.[382,383] Additionally, Castilho et al.[384] conducted a quantitative and qualitative comparative evaluation of SMT and NMT using automatic metrics and input from a small group of

professional translators. The evaluation focused on the translation of educational texts in four language pairs: English to Greek, German, Portuguese, and Russian.

Multilingualism

The following studies investigate various aspects of language analysis and processing across multiple languages. Giorgi et al.[385] developed a cognitive architecture based on a large-scale NN for processing and producing four natural languages simultaneously, exhibiting disambiguation in semantic and grammatical levels. The architecture was evaluated using two approaches, namely individual training for each language and cumulative training across all languages, demonstrating competence in comprehending and responding to pre-school literature questions. Gamallo et al.[386] measured intra-linguistic distances between six isolated European languages (including Greek), and the inter-linguistic distances with other European languages. Various linguistic measures were deployed to assess the distance between language pairs. Fragkou[387] conducted language identification using forums containing Greek, English, and Greeklish content and employed techniques borrowed from topic change segmentation. Bollegala et al.[388] automatically detected translations for biomedical terms and introduced a dimensionality reduction technique.

Cross-lingual NLP

MT helps address the scarcity of Greek LRs. This is achieved either through data augmentation or by enabling the use of English LRs and leveraging NMT models to bridge the language gap. Moreover, multilingual PLMs facilitate the knowledge transfer across languages. Papaioannou et al.[22] investigated cross-lingual knowledge-transfer strategies for clinical phenotyping. They evaluated three approaches: (1) translating Greek and Spanish notes into English before using a medical-specific English encoder, (2) using multilingual encoders,[49] and (3) expanding multilingual encoders with adapters.[389] Their findings indicated that the second approach lags behind the other two. They also recommended the most suitable method based on specific scenarios. The source code is available.[390]

The remaining studies either used data augmentation or translated text into English (to use English systems) for task resolution. To augment their data and address class imbalance, Patsiouras et al.[238] (see track: sentiment analysis and argument mining) translated tweets from the minority class from Greek into English and back. They included back-translated sentences only if these retained the original meaning while differing in wording. Additionally, they augmented data by substituting synonyms using a lexicon. Athanasiou and Maragoudakis[19] automatically translated original Greek data into English and used both the original and translated data for SA (track: sentiment analysis and argument mining). Manias et al.[229] examined the impact of NMT on SA by comparing English DNN LMs applied to both source (English) and target (Greek or German) languages for SA (track: sentiment analysis and argument mining). Singh et al.[391] did not use augmentation but proposed a cross-lingual augmentation approach by replacing part of the natural language input with its translation into another language. This was assessed across 14 languages, including Greek, in NLI (track: semantics) and QA (track: question answering). For the latter task, Mountantonakis et al.[21] translated user questions from Greek to English, employing English QA LMs for answer retrieval and then translating the answers back to Greek. Similarly, Papadopoulos et al.[20] developed a Transformer-based NMT system for English-Greek and from Greek-English translation.[392] By then using the English translations as input, they performed Open IE (track: information extraction), and the responses were translated back into Greek. A similar strategy was followed by Shukla et al.,[338] the top team in a financial narrative summarization shared task,[334] who used text classification for English reports and then translated the top-rated narrative sections into Greek (and Spanish) (track: summarization). MT has also been applied in Greek SA to enable English lexicon-based feature extraction.[245]

Machine translation, multilingualism, and cross-lingual NLP in Greek: Language resources

Table 16 presents multilingual LRs created by the studies discussed in this track. Prokopidis et al.[393] created multilingual corpora (756 language pairs) from content available on Global Voices,[394] a platform where volunteers publish and translate news stories in 41 languages. The Greek documents number 3,629 and are translated into 40 languages; however, not all documents were translated into every language, resulting in 17,018 document pairs in total. The resource of Bollegala et al.[388] consists of pairs of biomedical terms in multiple languages, comprising also the corresponding character n-gram and contextual features. Giorgi et al.[385] created a dataset simulating parent-child verbal interactions featuring a fictional 4-year-old. Initially in English, the dataset includes around 700 sentences (declarative and interrogative), translated into Greek, Italian, and Albanian.

Multilingual datasets for MT comprising Greek can be found in online repositories, such as Hugging Face[397] or the CLARIN:EL repository.[398] Hugging Face, as accessed on June 15, 2023, hosts a collection of 35 multilingual parallel corpora[399] and 49 multilingual LMs,[400] created by only seven teams, where Greek is one of the many hosted languages. CLARIN:EL hosts 25 multilingual and 200 bilingual corpora, with many of them being derived from the multilingual datasets within the repository. Therefore, the availability of potential LRs for MT in Greek (e.g., in Hugging Face)[401] is not reflected in the corresponding number of studies in our survey. This is mainly due to the fact that our search protocol (search protocol; Note S1) could not capture recent NMT research papers encompassing numerous languages that were not explicitly mentioned in the title or the abstract.

Summary of machine translation, multilingualism, and cross-lingual NLP in Greek

Work related to MT, multilingualism, and cross-lingual NLP in Greek primarily focuses on evaluation, augmentation, and transformation purposes. Although the LRs in our survey (see Table 16) are limited in size, they still offer valuable insights. Additionally, useful multilingual resources, including Greek, are available online (see machine translation, multilingualism, and cross-lingual NLP in Greek: language resources). However, these resources might not always be accompanied by published papers. Even when such papers exist, they may not explicitly mention all the languages in their titles or abstracts, making these resources less visible and harder to document. This issue

Table 16. Multilingual LRs containing Greek, with information on availability, annotation type, size and size unit of the Greek part of the datasets, document pairs (for parallel datasets), and number of languages

Authors	Availability	Ann. type	Size	Size unit	Document pairs	Languages
Prokopidis et al.[393]	yes[395]	user-generated	3,581	document	17,018	41
Bollegala et al.[388]	Lmt[396]	automatic	–	term	N/A	5
Giorgi et al.[385]	no	manual	700	sentence	N/A	4

Yes, publicly available; Lmt, limited availability; no, no information provided (see Table 3 for details). For annotation type, see Table 4 for details. The references cited include URLs.

arises because research related to NMT often involves multilingual NMT models that enable translation between multiple language pairs using a single model.[402] Building NMT models for each language pair is impractical, even when parallel data are available.[403] As a result, papers on multilingual research rarely include all the languages in their titles or abstracts, making it challenging for our search protocol to identify relevant studies.

TRACK: NLP APPLICATIONS

In addition to the thematic topics discussed thus far, this section presents studies with NLP applications across various domains.

NLP applications in Greek: Language models and methods

The studies we identified leverage NLP tasks for applications in the legal, business, educational, clinical, and media domains in Greek.

Legal

Papaloukas et al.[404] conducted multiclass legal topic classification using a range of techniques, including traditional ML methods, RNN-based methods, and multilingual and monolingual Transformer-based methods, highlighting the efficacy of monolingual Transformer-based models. Lachana et al.[405] developed an information retrieval tool for legal documents that allows users to search for documents based on their unique number, keywords, or individual articles. The system identifies correlations among Greek laws based on their number format, extracts tags from legal documents using TF-IDF, and decomposes laws into articles using regular expressions. Garofalakis et al.[406] proposed a system that uses regular expressions and pattern matching to locate and update laws, consolidating the historical revisions of legal documents.

Business

Paraskevopoulos et al.[407] classified safety reports and workplace images. They employed a multimodal fusion pipeline, experimenting with Transformer-based text encoders, a visual Transformer-based model, and a visual CNN-based architecture to predict workplace safety audits. Boskou et al.[408] evaluated internal audits by combining 26 internal audit criteria into a single quality measure and applying linear regression with TF-IDF.

Education

Chatzipanagiotidis et al.[409] focused on readability classification for Greek. The task aims to assess whether a text is appropriate for a given group of readers with varying education levels, such as first (L1) or second language proficiency (L2), or in terms of special needs (e.g., due to cognitive disabilities). Using handcrafted features and conventional ML algorithms, the authors classified textbooks covering various school subjects as well as Greek as a second language.

Media

Piskorski et al.[410] overviewed SemEval-2023 Task 3, which focuses on detecting genre category, framing, and persuasion techniques in online news across nine languages, including Greek. Participants tackled three subtasks: categorizing articles into opinion, reporting, or satire; identifying frames among 14 generic options; and detecting persuasion techniques within paragraphs using a taxonomy of 23 techniques. Forty-one teams submitted entries for evaluation, with Greek, Georgian, and Spanish data used only for testing. Participants predominantly used Transformer-based models across all subtasks. In the News Genre Categorization subtask, they addressed data scarcity by combining multilingual datasets, using automatic translation, or finding similar datasets, with ensemble methods being popular. In both Framing detection and Persuasion Techniques detection subtasks, what differentiated the participating systems were the pre-processing and data-augmentation techniques.

Clinical

Athanasiou et al.[411] developed prediction models for influenza-like illness (ILI) outbreaks using ILI surveillance, weather, and Twitter data with LSTM NNs. They employed transfer learning to combine features from separate LSTM models for each data category. Results showed the transfer-learning model integrating all three data types outperformed models using individual sources. Stamouli et al.[412] analyzed transcribed spoken-narrative discourses using the ILSP Neural NLP toolkit (see track: syntax and grammar) to assess linguistic features relevant to language impairments. The results indicated no significant linguistic differences between remote and in-person data collection, which validates the feasibility of remote assessment.

NLP applications in Greek: Language resources

Table 17 displays datasets related to Greek NLP applications. The dataset presented by Papaloukas et al.,[404] consisting of legal resources from Greek legislation, is the only publicly available one. The corpus comes from a collection of Greek legislative documents titled "Permanent Greek Legislation Code – Raptarchis,"[413] classified from broader to more specialized categories. Paraskevopoulos et al.[407] shared a multimodal dataset, which is available upon request, containing 5,344 safety-related observations reported by 86 safety officers after inspecting 486 sites. Each observation includes a brief description of the issue, accompanying images, relevant metadata, and a priority score. Garofalakis et al.[406] provided an alpha version of the historical consolidation of 320 laws from the period 2004–2015, including the history of their revisions.

Table 17. Datasets related to NLP applications with information on availability, annotation type, size, size unit, domain, and annotation type

Authors	Availability	Ann. type	Size	Size unit	Domain	Annotation type
Papaloukas et al.[404]	yes[414]	curated[a]	47,563	document	legal	topic class
Paraskevopoulos et al.[407]	Lmt	manual	5,344	issue	industry	safety observation
Garofalakis et al.[406]	Lmt[415]	automatic	3,209	document	legal	law revision
Stamouli et al.[412]	no	hybrid	28,238	token	general	token, lemma, pos, syntax
Piskorski et al.[410]	no	manual	64	document	globally discussed topics	genre, framing and persuasion techniques
Lachana et al.[405]	no	automatic	70	document	legal	law revision
Boskou et al.[408]	no	manual	133	document	finance	internal audit criteria

Yes, publicly available; Lmt, limited availability; no, no information provided (see Table 3 for details). For annotation type, see Table 4 for details. The references cited include URLs.
[a]Unclear annotation process.

The following datasets are not publicly available. Stamouli et al.[412] transcribed 139 spoken narrative discourses from ten humans, while Piskorski et al.[410] described the dataset for the SemEval-2023 Task 3, which includes human annotations on genre, framing, and persuasion techniques in online news across nine languages. Lachana et al.[405] obtained a subset of Greek laws from the Government Gazette, establishing connections between the laws and categorizing the modification type of each law. Boskou et al.[408] compiled a corpus from the internal audit texts found in the annual reports of 133 publicly traded Greek companies for the year 2014. The texts were manually evaluated against 26 internal audit quality criteria.

Summary of NLP applications in Greek

Greek NLP applications span various domains, including legal, business, education, clinical, and media, with approaches often tailored to specific fields, such as consolidating historical revisions in the legal domain. Alternatively, they involve text classification tasks, such as assessing text readability education purposes. Additionally, approaches that are relatively uncommon in Greek NLP literature were employed, including multimodal approaches, information retrieval, and topic classification.

DISCUSSION

Challenges for monolingual NLP surveys

By undertaking a monolingual survey for Greek, we were able to identify two major challenges. We describe them below to assist future monolingual surveys for any language.

Missed multilingual entries

Monolingual surveys may operate in a reduced search space because the name of the language in question is part of the query. This poses particular challenges for studies focusing on specific languages within multilingual contexts, as such studies do not always explicitly mention all the languages involved in their titles or abstracts. However, the findings of our work reveal that 41% of the surveyed papers address multilingual research. While multilingual research may not be as extensively represented as monolingual research, it is sufficiently represented within the corpus examined.

False positives

On the other hand, the language name may be mentioned in the title or abstract not because it was the focus of the study but for a variety of other reasons (e.g., in the case of Greek, the etymology of a word, terminology, classical studies, etc.). Liu et al.,[416] for example, while mentioning an English term derived from a Greek one, explicitly mention the language in the abstract, which confused our search. The exact sentence was "meme is derived from the Greek word "Mimema" and refers to the idea being imitated." Another example is the work of Das et al.,[417] who referenced Ancient Greek, which falls outside the scope of this study. The exact sentence mentioned in the abstract was "Indian epics have not been analyzed computationally to the extent that Greek epics have."

The challenge of retrieving false positives was particularly pronounced for Greek. This is illustrated by the following observation. We compared the search results in Google Scholar for specific languages, sampled from well-supported (i.e., first-tier) or moderately supported (i.e., second-tier) languages (Figure 1). A search for the German language, which belongs to the first tier, yielded 5,100,000 results, for Arabic (first tier) 3,010,000, for Finnish (second tier) 2,440,000, and for Latin (second tier) 4,700,000. Greek (second tier) returned 4,840,000 results, a number that is comparable to or higher than languages with stronger support.

Our search protocol limited the search for the language name, i.e., "Greek," to the title or abstract of the papers. Meanwhile, the term "Natural Language Processing" was searched across the entire paper (see search protocol for details). This approach retrieved 1,135 unique papers, 142 of which were relevant to the survey's purpose. Although restricting the language name to the title or abstract might not capture all published papers for Greek NLP, it was a necessary measure to reduce the high number of false positives that could have been returned without this restriction.

Monolingual NLP survey scarcity

The level of the challenge ultimately depends on the language, but both issues mentioned above (missed multilingual entries and false positives) apply to some extent to any language. This added difficulty may explain the general scarcity of monolingual NLP surveys. By retrieving language-specific NLP surveys from Google Scholar, published between 2012 and 2023, we found that only 19% of well- and moderately supported languages have monolingual surveys for NLP (see monolingual NLP surveys in other languages). These surveys, however, do not provide the search protocol used, hindering reproducibility and

interpretability, nor do they classify the available LRs for quality and licensing, which makes them of limited use for experiments with multilingual and LLMs. While we did not search for NLP surveys in each of the thousands of less-supported languages, we acknowledge that the situation is likely worse for them, likely due to the limited attention they have received from the NLP research community.

Greek NLP trends

NLP tracks trends

From the outcomes of our survey, we were able to extract insights regarding trends in the NLP tracks. These shed light on shifts in the prevalence of NLP thematic areas in Greek from 2012 to 2023. Figure 4 illustrates the relative track popularity, with each presented as a time series (bold blue line), as opposed to the rest of the tracks that appear shadowed in the background.

SA emerged as the most prominent topic, constituting about a quarter of publications (see Figure 4), yet shows a declining trend since 2018, echoing global patterns.[28] In contrast, the tracks that have gained focus in recent years and align with global trends are ethics and NLP, summarization, IE, and NLP applications. Their gained focus is likely driven by the rise of Transformer-based PLMs, which also explains the recent relative interest in ML for NLP.

Semantics experiences fluctuations across the years, with three peaks. The latest peak, in 2020, is smaller than the initial peak in 2014, signaling a decline in interest after a slight rise around 2018. Notably, semantics studies in Greek predominantly focus on lexical semantics, rather than sentence-level semantics or discourse analysis. Syntax maintains interest within the Greek NLP community. This contrasts with global trends, as reflected in the shrinking number of submissions to ACL tracks focused on syntax: tagging, chunking, and parsing.[28]

MT showed a decline in interest in 2023, following a slight peak in the preceding years. This aligns with global trends,[28] where interest peaked in the early 2010s and has been steadily declining since. In this survey, we may have missed papers addressing research in a multilingual context (challenges for monolingual NLP surveys), which is primarily the case in MT, so the trends may not be entirely accurate for this track. Authorship analysis also shows a decline in interest, with the most recent paper dating to 2019 and the majority of publications concentrated between 2012 and 2017. Notably, studies within this topic primarily focus on traditional tasks like author profiling and authorship attribution and verification, rather than exploring newer tasks such as profiling of robots or detection of style change.

Shared tasks for Greek

Shared tasks are a well-established method for advancing NLP research, helping to define best practices and introduce new datasets.[418] Therefore, it is crucial to include less-supported languages in these task-specific events. For Greek, the tasks in shared tasks and workshops that address its context are author identification, author clustering, offensive language detection, and summarization. Specifically, the PAN Workshop series focused on Greek for authorship analysis tasks, such as author identification and clustering, from 2013 to 2016 (track: authorship analysis). In 2020, the OffensEval-2020 shared task included Greek in its offensive language detection challenge (track: ethics and NLP). The most recent is the FNS shared task from the FNP workshop series, which, since 2022, targets summarization for the Greek context. summarization for Greek was also addressed in an earlier workshop, MultiLing 2013 (track: summarization).

Analyzing Greek datasets

Dataset availability

We analyzed the Greek datasets developed by the authors of the surveyed studies with regard to their availability as defined in LR availability categories: each category corresponds to specific criteria applied to the resource's URL, license, and data format. A total of 94 datasets were identified, with more than half (59.6%) characterized as available by their creators. However, only 14.8% of the total datasets are publicly available according to our availability schema, meaning they are accessible, licensed, and in machine-readable format. This highlights the limited proportion of truly open resources despite claims of availability.

Among the remaining datasets, 33.3% are of limited availability. These include datasets that either lack a license, require a fee for access, or are accessible only upon request. Notably, 11.7% of the datasets yielded an HTTP error during our access attempts. This issue stems from storage on web pages that often lack proper maintenance and curation, such as institutional repositories or personal websites. These web pages often do not provide the same preservation guarantees as established trusted data repositories, such as GitHub[419] (used by Evdaimon et al.,[23] Perifanos and Goutsos,[83] Bilianos,[86] Garí Soler and Apidianaki,[108] Korre et al.,[141] and Kavros and Tzitzikas[144]), Zenodo[420] (used by Dritsa et al.,[167] Papantoniou et al.,[202] Stamatatos et al.,[280] and Fitsilis and Mikros[421]), Hugging Face[397] (used by Zampieri et al.,[310] Koniaris et al.,[343] and Papaloukas et al.[404]), or CLARIN:EL[398] (used by Pontiki et al.[318]).The largest category, comprising 40.4% of the datasets, includes those for which no availability information was provided. We observed that 31 of these 40 datasets are human annotated, underscoring a significant missed opportunity to expand the pool of gold-standard resources for Greek NLP.

Expanding this analysis further, Figure 5 presents the availability of Greek LRs over the years. Publicly available datasets (depicted in green) have been consistently provided since 2020, reflecting recent trends favoring data sharing and open access.[422] In contrast, datasets with no availability information (in black) and those of limited availability (in orange) have persisted across all years, often representing a significant proportion of the datasets in each year. This observation indicates that issues of restricted access are systemic and not confined to earlier periods. Datasets inaccessible due to HTTP errors (in red) appear sporadically across the years, likely due to inadequate storage maintenance.

Standardization of annotations

Annotated datasets constitute the majority of datasets developed in the surveyed papers, underscoring the importance of standardized annotation schemes for methodological comparability, especially for tasks where consistency and comparability across methodologies are crucial. Standardized annotations enable consistent benchmarks, enhance comparability of methods, and foster integration with broader research

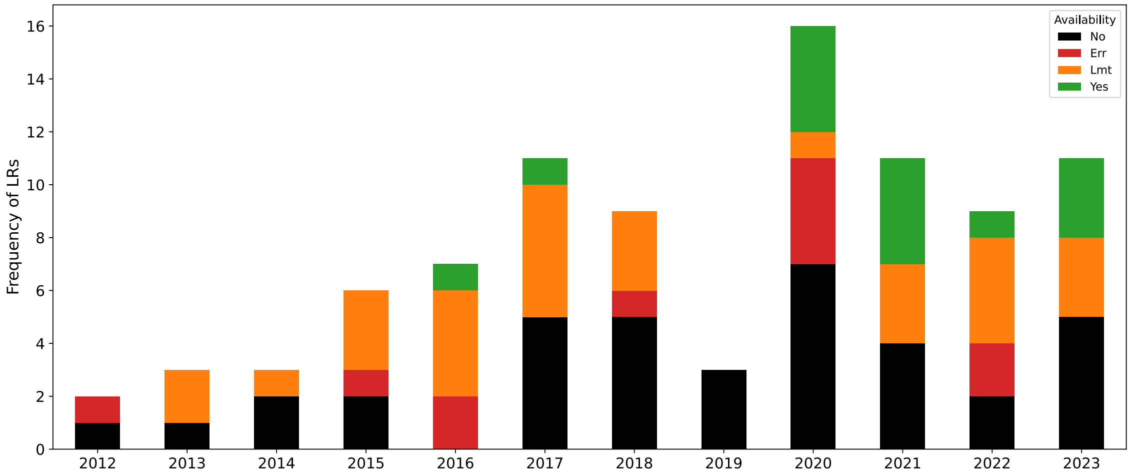

Figure 5. Number of Greek datasets per year according to their availability classification
Yes, publicly available; Lmt, limited public availability; Err, unavailable; No, no information provided; see Table 3 for more details.

efforts. For example, in syntax and GEC, standardized annotations are vital. Prokopidis and Papageorgiou[127] developed the Greek UD treebank for dependency parsing. This is part of the UD project[150] that offers standardized treebanks and provides consistent and unified annotation practices across languages. Korre et al.[141] established an annotation schema for grammatical error types in Greek. Similarly, in NER, Bartziokas et al.[205] provided two annotated datasets, one with four label tags akin to the CONLL-2003 dataset[206] and the other incorporating 18 tags for entities, as in the OntoNotes 5 English dataset,[207] enabling direct comparison across languages and studies. Standardized guidelines are also critical in toxicity detection and in SA, where annotation consistency is needed to compare models addressing sensitive or subjective content. For example, Zampieri et al.[310] provided a hierarchical three-level annotation schema for offensive language identification. In SA, the majority of studies performing emotion detection were based on Ekman's six basic emotions as a standard framework.

Multilingual nature of datasets

In terms of linguality (number of languages covered, i.e., mono- or multilingual), approximately 86% of the datasets are monolingual, while the remaining portion comprises bilingual or multilingual datasets. Furthermore, only a small fraction (5.3%) of all datasets comprises translations into Greek. This limited reliance on translations is advantageous, as translations may not faithfully capture the natural linguistic features and nuances of native Greek speakers.[362] The emphasis on native Greek datasets enhances the representativeness and quality of language resources for NLP tasks.

Greek NLP datasets: Raw and multitask

The LRs presented thus far include annotations for specific NLP tasks. Additionally, we identified corpora that provide raw text without any meta-information as well as those with meta-information that can be used for various NLP tasks. Table 18 shows all these datasets in Greek, classified by their availability, annotation type, and any automatically extracted meta-information included.

Greek datasets with no annotations

Datasets without annotations are versatile resources that are potentially applicable to various NLP tasks beyond their original intended purposes. For instance, publicly available raw LRs can facilitate pre-training purposes, such as (masked) language modeling. Prokopidis and Piperidis[74] created a dataset of 101,857 open-content web pages, comprising online archives of Greek newspapers from 2003 to 2020 and the Greek part of the w2c corpus,[428] scraped and pre-processed. Iosif et al.[162] shared a dataset consisting of web-document snippets in English, German, Italian, and Greek, with the Greek portion comprising 66M.

Greek datasets for various NLP tasks

Fitsilis and Mikros[421] assembled a corpus of 2,000 parliamentary questions from 2009 to 2019, corresponding to 638,865 tokens, while Dritsa et al.[167] processed 1.28M political speeches from Greek parliamentary records, spanning from July 1989 to July 2020. Both datasets are publicly available, and they comprise automatically extracted metadata related to parliamentary procedures (e.g., parliamentary period or political party). Barzokas et al.[168] generated a corpus of 34.88M tokens by processing e-books from Project Gutenberg[429] and OpenBook.[430] Lopes et al.[372] developed a dataset comprising audio recordings and transcripts of 200 dialogs from call-center interactions regarding movie inquiries, annotated with gender, task success, anger, satisfaction, and miscommunication. Additionally, Lioudakis et al.[164] compiled a dataset sourced from the online corpus of the newspaper *Macedonia*,[431] consisting of 8,005 articles annotated with their respective topics.

Emerging Greek benchmark datasets

Table 19 summarizes Greek datasets from our survey that qualify as benchmarks for NLP research. These datasets meet strict criteria. First, they should be publicly available (see the definition in availability taxonomy). That is, they should be accessible, licensed, free of charge, and in a machine-readable format. Second, they should include human-generated annotations (see the definition in annotation type taxonomy), which are classified as

Table 18. Raw or annotated Greek datasets for various NLP tasks classified according to their availability, annotation type, size, size unit, and automatically extracted meta-information

Authors	Availability	Ann. type	Size	Size unit	Meta-information
Dritsa et al.[167]	yes[423]	automatic	1.28M	political speech	member name, sitting date, parliamentary period, parliamentary session, parliamentary sitting, political party, government, member region, roles, member gender, speech
Fitsilis and Mikros[421]	yes[424]	automatic	2,000	parliamentary question	question type, subject, parliamentary period, parliamentary session, political party, submitter, ministers, ministers
Prokopidis and Piperidis[74]	yes[425]	no annotation	101,857	web page	N/A
Barzokas et al.[168]	yes[426]	curated	34.88M	token	literature type, author, publication year, ISBN
Lopes et al.[372]	Lmt	manual	200	dialog	gender, task success, anger, satisfaction, miscommunication
Lioudakis et al.[164]	Err[214]	curated[a]	8,005	article	topic
Iosif et al.[162]	Err[427]	no annotation	66M	web document snippet	N/A

Yes, publicly available; Lmt, limited availability; Err, unavailable (see Table 3 for details). For annotation type, see Table 4 for details. The references cited include URLs.
[a]Unclear annotation process.

"manual," "curated," "user-generated" or "hybrid" in Tables 6, 7, 8, 9, 10, 11, 12, 13, 14, 15, 16, and 17.

Of the 91 annotated datasets identified across all NLP track sections, only nine meet these criteria. While most provide train-test splits, reducing data leakage risks, four datasets lack split specifications,[141,168,203,393] necessitating caution in evaluation use. These datasets span across nine different NLP tasks, including summarization, NER, intent classification, topic classification, GEC, toxicity detection, syntactical and morphological analysis, MT, and text classification.

By revisiting the LR tables of the NLP track sections (Tables 6, 7, 8, 9, 10, 11, 12, 13, 14, 15, 16, and 17), certain resources marked as "Lmt" in the availability-type column could potentially become publicly available through proper licensing. Similarly, resources marked as "Err" could be converted into accessible benchmark datasets by curating their storage pages. These steps could transform 17 additional datasets into actionable benchmarks, accelerating progress in undersupported tasks such as SA and authorship analysis.

Challenges and opportunities for Greek in the LLM era

Our work offers a detailed account of the landscape of Greek NLP, highlighting not only the evolution of research themes but also the status of available LRs and licensing practices. By identifying openly available datasets and models (see Tables 5 and 19), this study lays important groundwork for the future tuning and training of LLMs tailored to Greek. However, this forward-looking potential must be approached with caution, especially in light of the unique challenges posed by limited linguistic representation in current LLMs.

LLMs perform best on languages that are well represented in their training data. For Greek—likely constituting only a small fraction of such datasets—models have limited exposure to its syntax, morphology, and usage patterns, as well as to idiomatic expressions, named entities, and culturally specific references. As a result, generated text in Greek may be grammatically awkward, semantically imprecise, and contextually or culturally inappropriate.[432] Furthermore, LLMs trained predominantly on English and other high-resource languages may misinterpret

Table 19. Emerging Greek NLP benchmark datasets: Publicly available Greek datasets with human-generated annotations

Authors	Dataset	Task	Split
Koniaris et al.[343]	DominusTea/GreekLegalSum[346]	summarization	yes
Rizou et al.[203]	Uniway[211]	ner, intent cl.	no
Papaloukas et al.[404]	AI-team-UoA/greek_legal_code[414]	topic cl.	yes
Korre et al.[141]	GNC,[147] GWE[147]	gec	no
Bartziokas et al.[205]	elNER[212]	ner	yes[a]
Zampieri et al.[310]	strombergnlp/offenseval_2020[323]	toxicity det.	yes
Barzokas et al.[168]	openbook, project_gutenberg[426]	text cl.	no
Prokopidis and Papageorgiou[127]	UD_Greek-GDT[148]	syntax	yes
Prokopidis et al.[393]	PGV[395]	mt	no

Train/test splits are indicated. The dataset references cited include URLs.
[a]Only training splits.

polysemous words, struggle with dialectal variation and code switching, and default to English-centric assumptions even when interacting in Greek.[106] Consequently, responses to Greek queries often reflect cultural norms and worldviews rooted in English-language data, while Greek-specific historical, legal, or societal knowledge may be omitted or distorted.

As LLMs become increasingly embedded in everyday applications, the risk of excluding speakers of variations of the Greek language (including regional variants) grows. Such users may experience miscommunication or reduced access to services or feel culturally invisible within AI systems. Over time this could reinforce social and linguistic hierarchies, further marginalizing non-standard language communities. There is thus a pressing need for more open, high-quality, and properly licensed Greek language resources—especially from communicative contexts—that can be ingested by LLMs to improve linguistic coverage, fairness, and inclusivity.

CONCLUSIONS

Our work achieves two primary goals. First, we introduce a generalizable methodology for conducting systematic monolingual NLP surveys. By addressing the lack of standardized frameworks for monolingual surveys, we provide a replicable approach that minimizes selection bias, ensures reproducibility, and organizes findings into coherent thematic tracks. The second goal concerns our application of this methodology to create a comprehensive survey of Greek NLP from 2012 to 2023. This methodology not only advances Greek NLP but also serves as a blueprint for undersupported languages worldwide.

A key contribution of our survey is the thorough cataloging of Greek LRs, including nine publicly available, human-annotated datasets spanning nine NLP tasks, such as summarization, NER, and MT. These resources hold significant potential as benchmarks for advancing Greek NLP research. While Greek remains resource scarce in certain tasks (e.g., SA), we have addressed LRs that, with licensing or maintenance resolution, can be converted easily to benchmarks. Our analysis of methodological shifts reveals that while DL dominates post 2019, traditional ML methods persist in certain tasks, signaling opportunities for more innovated approaches. Additionally, Greek NLP favors monolingual language models (e.g., GreekBERT) over multilingual systems, achieving state-of-the-art results in tasks such as SA. This preference underscores the importance of language-specific pre-training. Task-specific trends further illustrate Greek NLP's unique trajectory: while global interest in syntax declines, Greek retains a strong focus, likely due to its morphological complexity. Conversely, SA research declines locally, mirroring broader shifts toward emergent tasks like ethics and NLP.

To ensure accessibility and longevity, we host our survey results in an online repository, designed as a continuously evolving resource for the NLP community. Our systematic methodology ensures unbiased and replicable results, setting a standard for future monolingual surveys. By addressing resource disparities in Greek NLP and providing a replicable framework, our work bridges the gap between monolingual and multilingual NLP research, promoting inclusivity and equitable progress for undersupported languages worldwide.

RESOURCE AVAILABILITY

Lead contact

Requests for further information and resources should be directed to and will be fulfilled by the lead contact, John Pavlopoulos (annis@aueb.gr).

Materials availability

This study did not generate new materials.

Data and code availability

Original data have been deposited to Zenodo: https://doi.org/10.5281/zenodo.15314882,[433] including metadata of the surveyed papers/datasets and figures illustrating key findings on Greek NLP.

ACKNOWLEDGMENTS

This work has been partially supported by project MIS 5154714 of the National Recovery and Resilience Plan Greece 2.0 funded by the European Union under the NextGenerationEU Program.

AUTHOR CONTRIBUTIONS

Conceptualization, J.B. and J.P.; methodology, J.B., K.P., M.G., and J.P.; investigation, J.B. and K.P.; writing – original draft, J.B.; writing – review & editing, J.B., K.P., M.G., and J.P.; project administration, J.B. and J.P.

DECLARATION OF INTERESTS

The authors declare no competing interests.

SUPPLEMENTAL INFORMATION

Supplemental information can be found online at https://doi.org/10.1016/j.patter.2025.101313.

Received: February 3, 2025
Revised: March 17, 2025
Accepted: June 6, 2025
Published: July 21, 2025

REFERENCES

1. Vaswani, A., Shazeer, N., Parmar, N., Uszkoreit, J., Jones, L., Gomez, A. N., Kaiser, L.u., and Polosukhin, I. (2017). Attention is all you need. In Advances in Neural Information Processing Systems, *30*, I. Guyon, U.V. Luxburg, S. Bengio, H. Wallach, R. Fergus, S. Vishwanathan, and R. Garnett, eds. *(Curran Associates, Inc.), pp. 5998–6008.* https://proceedings.neurips.cc/paper_files/paper/2017/file/3f5ee243547dee91fbd053c1c4a845aa-Paper.pdf.
2. Brown, T.B., Mann, B., Ryder, N., Subbiah, M., Kaplan, J.D., Dhariwal, P., Neelakantan, A., Shyam, P., Sastry, G., Askell, A., et al. (2020). Language models are few-shot learners. In Advances in Neural Information Processing Systems, *33* (Curran Associates, Inc.), pp. 1877–1901. https://proceedings.neurips.cc/paper/2020/file/1457c0d6bfcb4967418bfb8ac142f64a-Paper.pdf.
3. Pires, T., Schlinger, E., and Garrette, D. (2019). How multilingual is multilingual bert? In Proceedings of the 57th Annual Meeting of the Association for Computational Linguistics, pp. 4996–5001.
4. Qin, L., Chen, Q., Zhou, Y., Chen, Z., Li, Y., Liao, L., Li, M., Che, W., and Yu, P.S. (2025). A survey of multilingual large language models. Patterns *6*, 101118. https://doi.org/10.1016/j.patter.2024.101118.
5. Blasi, D., Anastasopoulos, A., and Neubig, G. (2022). Systematic inequalities in language technology performance across the world's languages. In Proceedings of the 60th Annual Meeting of the Association for Computational Linguistics (Volume 1: Long Papers), S. Muresan, P. Nakov, and A. Villavicencio, eds. (Association for Computational

Linguistics), pp. 5486–5505. https://doi.org/10.18653/v1/2022.acl-long.376.

6. Bender, E.M., Gebru, T., McMillan-Major, A., and Shmitchell, S. (2021). On the dangers of stochastic parrots: Can language models be too big? In Proceedings of the 2021 ACM conference on fairness, accountability, and transparency, pp. 610–623.
7. Oflazer, K. (2014). Turkish and its challenges for language processing. Lang. Resour. Eval. *48*, 639–653. https://doi.org/10.1007/s10579-014-9267-2.
8. Shoufan, A., and Alameri, S. (2015). Natural language processing for dialectical Arabic: A survey. In Proceedings of the second workshop on Arabic natural language processing, pp. 36–48.
9. Al-Ayyoub, M., Nuseir, A., Alsmearat, K., Jararweh, Y., and Gupta, B. (2018). Deep learning for Arabic NLP: A survey. Journal of computational science *26*, 522–531. https://doi.org/10.1016/j.jocs.2017.11.011.
10. Larabi Marie-Sainte, S., Alalyani, N., Alotaibi, S., Ghouzali, S., and Abunadi, I. (2019). Arabic natural language processing and machine learning-based systems. IEEE Access *7*, 7011–7020. https://doi.org/10.1109/ACCESS.2018.2890076.
11. Papantoniou, K., and Tzitzikas, Y. (2020). NLP for the Greek language: a brief survey. In 11th Hellenic Conference on Artificial Intelligence, pp. 101–109. https://doi.org/10.1145/3411408.3411410.
12. Alam, F., Hasan, A., Alam, T., Khan, A., Tajrin, J., Khan, N., and Chowdhury, S.A. (2021). A review of bangla natural language processing tasks and the utility of transformer models. Preprint at arXiv. https://doi.org/10.48550/arXiv.2107.03844.
13. Hämäläinen, M., and Alnajjar, K. (2021). The Current State of Finnish NLP. In Proceedings of the Seventh International Workshop on Computational Linguistics of Uralic Languages, pp. 65–72.
14. Desai, N.P., and Dabhi, V.K. (2021). Taxonomic survey of Hindi Language NLP systems. Preprint at arXiv. https://doi.org/10.48550/arXiv.2102.00214.
15. Guellil, I., Saâdane, H., Azouaou, F., Gueni, B., and Nouvel, D. (2021). Arabic natural language processing: An overview. Journal of King Saud University-Computer and Information Sciences *33*, 497–507. https://doi.org/10.1016/j.jksuci.2019.02.006.
16. Darwish, K., Habash, N., Abbas, M., Al-Khalifa, H., Al-Natsheh, H.T., Bouamor, H., Bouzoubaa, K., Cavalli-Sforza, V., El-Beltagy, S.R., El-Hajj, W., et al. (2021). A panoramic survey of natural language processing in the Arab world. Commun. ACM *64*, 72–81. https://doi.org/10.1145/3447735.
17. Rajendran, S., Anand Kumar, M., Rajalakshmi, R., Dhanalakshmi, V., Balasubramanian, P., and Soman, K. (2022). Tamil NLP Technologies: Challenges, State of the Art, Trends and Future Scope. In International Conference on Speech and Language Technologies for Low-resource Languages (Springer), pp. 73–98.
18. Gonzalez-Dios, I., and Altuna, B. (2022). Natural Language Processing and Language Technologies for the Basque Language. Cuadernos Europeos de Deusto *20*, 203–230. https://doi.org/10.18543/ced.2477.
19. Athanasiou, V., and Maragoudakis, M. (2017). A novel, gradient boosting framework for sentiment analysis in languages where NLP resources are not plentiful: A case study for modern Greek. Algorithms *10*, 34. https://doi.org/10.3390/a10010034.
20. Papadopoulos, D., Papadakis, N., and Matsatsinis, N.F. (2021). PENELOPIE: enabling open information extraction for the Greek language through machine translation. Preprint at arXiv. https://doi.org/10.48550/arXiv.2103.15075.
21. Mountantonakis, M., Bastakis, M., Mertzanis, L., and Tzitzikas, Y. (2022). Tiresias: Bilingual question answering over dbpedia. In Workshop at ISWC. CEUR Workshop Proceedings (CEUR-WS.org).
22. Papaioannou, J.M., Grundmann, P., van Aken, B., Samaras, A., Kyparissidis, I., Giannakoulas, G., Gers, F., and Loeser, A. (2022). Cross-lingual knowledge transfer for clinical phenotyping. In Proceedings of the Thirteenth Language Resources and Evaluation Conference. Marseille, France: European Language Resources Association, N. Calzolari, F. Béchet, P. Blache, K. Choukri, C. Cieri, T. Declerck, S. Goggi, H. Isahara, B. Maegaard, J. Mariani, and et al.., eds., pp. 900–909.
23. Evdaimon, I., Abdine, H., Xypolopoulos, C., Outsios, S., Vazirgiannis, M., and Stamou, G. (2024). Greekbart: The first pretrained Greek sequence-to-sequence model. In Proceedings of the 2024 Joint International Conference on Computational Linguistics, Language Resources and Evaluation (LREC-COLING 2024), pp. 7949–7962.
24. Ranasinghe, T., and Zampieri, M. (2021). Multilingual offensive language identification for low-resource languages. ACM Trans. Asian Low-Resour. Lang. Inf. Process. *21*, 1–13. https://doi.org/10.1145/3457610.
25. Koutsikakis, J., Chalkidis, I., Malakasiotis, P., and Androutsopoulos, I. (2020). Greek-bert: The Greeks visiting sesame street. In 11th Hellenic Conference on Artificial Intelligence. SETN 2020 (Association for Computing Machinery), pp. 110–117. https://doi.org/10.1145/3411408.3411440.
26. Ahn, H., Sun, J., Park, C.Y., and Seo, J. (2020). Nlpdove at semeval-2020 task 12: Improving offensive language detection with cross-lingual transfer. Preprint at arXiv. https://doi.org/10.48550/arXiv.2008.01354.
27. Zampieri, M., Rosenthal, S., Nakov, P., Dmonte, A., and Ranasinghe, T. (2023). Offenseval 2023: Offensive language identification in the age of large language models. Nat. Lang. Eng. *29*, 1416–1435. https://doi.org/10.1017/S1351324923000517.
28. Rohatgi, S., Qin, Y., Aw, B., Unnithan, N., and Kan, M.Y. (2023). The ACL OCL corpus: Advancing open science in computational linguistics. In Proceedings of the 2023 Conference on Empirical Methods in Natural Language Processing, H. Bouamor, J. Pino, and K. Bali, eds. (Association for Computational Linguistics), pp. 10348–10361. https://doi.org/10.18653/v1/2023.emnlp-main.640.
29. Bender, E.M. The #benderrule: On naming the languages we study and why it matters. https://thegradient.pub/the-benderrule-on-naming-the-languages-we-study-and-why-it-matters/.
30. Phillips, A., and Davis, M. (2009). Tags for identifying languages (rfc 5646). Internet Engineering Task Force (IETF). https://datatracker.ietf.org/doc/html/rfc5646.html.
31. SIL International. Ethnologue: Languages of the world. https://www.ethnologue.com/.
32. Gavriilidou, M., Giagkou, M., Loizidou, D., and Piperidis, S. (2023). Language report Greek. In European Language Equality: A Strategic Agenda for Digital Language Equality, G. Rehm and A. Way, eds. (Springer), pp. 151–154. https://doi.org/10.1007/978-3-031-28819-7_19.
33. Davies, A.M. (2015). Greek Language (Oxford University Press). https://doi.org/10.1093/acrefore/9780199381135.013.2895.
34. Gavriilidou, M., Koutsombogera, M., Patrikakos, A., and Piperidis, S. (2012). The Greek Language in the Digital Age. In META-NET White Paper Series, G. Rehm and H. Uszkoreit, eds. (Springer).
35. Tzanidaki, D.I. (1995). Greek word order: towards a new approach. UCL Working Papers in Linguistics 7, 247–277.
36. Manning, C.D. (2015). Last words: Computational linguistics and deep learning. Comput. Linguist. *41*, 701–707. https://doi.org/10.1162/COLI_a_00239.
37. Qiu, X., Sun, T., Xu, Y., Shao, Y., Dai, N., and Huang, X. (2020). Pre-trained models for natural language processing: A survey. Sci. China Technol. Sci. *63*, 1872–1897. https://doi.org/10.1007/s11431-020-1647-3.
38. Bommasani, R., Hudson, D.A., Adeli, E., Altman, R., Arora, S., von Arx, S., Bernstein, M.S., Bohg, J., Bosselut, A., Brunskill, E., et al. (2021). On the opportunities and risks of foundation models. Preprint at arXiv. https://doi.org/10.48550/arXiv.2108.07258.
39. Minaee, S., Mikolov, T., Nikzad, N., Chenaghlu, M., Socher, R., Amatriain, X., and Gao, J. (2024). Large language models: A survey. Preprint at arXiv. https://doi.org/10.48550/arXiv.2402.06196.

40. Pascanu, R., Mikolov, T., and Bengio, Y. (2013). On the difficulty of training recurrent neural networks. In International conference on machine learning (Pmlr), pp. 1310–1318.

41. Rumelhart, D.E., Hinton, G.E., and Williams, R.J. (1986). Learning representations by back-propagating errors. Nature *323*, 533–536. https://doi.org/10.1038/323533a0.

42. Elman, J.L. (1990). Finding structure in time. Cogn. Sci. *14*, 179–211. https://doi.org/10.1207/s15516709cog1402_1.

43. Werbos, P.J. (1988). Generalization of backpropagation with application to a recurrent gas market model. Neural Netw. *1*, 339–356. https://doi.org/10.1016/0893-6080(88)90007-X.

44. Devlin, J., Chang, M.W., Lee, K., and Toutanova, K. (2019). BERT: Pre-training of deep bidirectional transformers for language understanding. In Proceedings of the 2019 Conference of the North American Chapter of the Association for Computational Linguistics: Human Language Technologies, Volume 1 (Long and Short Papers), J. Burstein, C. Doran, and T. Solorio, eds. (Association for Computational Linguistics), pp. 4171–4186. https://doi.org/10.18653/v1/N19-1423.

45. Liu, Y., Ott, M., Goyal, N., Du, J., Joshi, M., Chen, D., Levy, O., Lewis, M., Zettlemoyer, L., and Stoyanov, V. (2019). Roberta: A robustly optimized bert pretraining approach. Preprint at arXiv. https://doi.org/10.48550/arXiv.1907.11692.

46. Lan, Z., Chen, M., Goodman, S., Gimpel, K., Sharma, P., and Soricut, R. (2020). Albert: A lite bert for self-supervised learning of language representations. Preprint at arXiv. https://doi.org/10.48550/arXiv.1909.11942.

47. He, P., Liu, X., Gao, J., and Chen, W. (2021). Deberta: Decoding-enhanced bert with disentangled attention. Preprint at arXiv. https://doi.org/10.48550/arXiv.2006.03654.

48. Lample, G., and Conneau, A. (2019). Cross-lingual language model pretraining. Preprint at arXiv. https://doi.org/10.48550/arXiv.1901.07291.

49. Conneau, A., Khandelwal, K., Goyal, N., Chaudhary, V., Wenzek, G., Guzmán, F., Grave, É., Ott, M., Zettlemoyer, L., and Stoyanov, V. (2020). Unsupervised cross-lingual representation learning at scale. In Proceedings of the 58th Annual Meeting of the Association for Computational Linguistics, pp. 8440–8451.

50. Yang, Z., Dai, Z., Yang, Y., Carbonell, J., Salakhutdinov, R., and Le, Q.V. (2020). Xlnet: Generalized autoregressive pretraining for language understanding. Preprint at arXiv. https://doi.org/10.48550/arXiv.1906.08237.

51. Hugging Face. Bertology documentation. https://huggingface.co/docs/transformers/bertology.

52. Radford, A., Narasimhan, K., Salimans, T., and Sutskever, I. (2018). Improving Language Understanding by Generative Pre-training (OpenAI).

53. Radford, A., Wu, J., Child, R., Luan, D., Amodei, D., and Sutskever, I. (2019). Language Models Are Unsupervised Multitask Learners (OpenAI). https://openai.com/blog/language-unsupervised.

54. Raffel, C., Shazeer, N., Roberts, A., Lee, K., Narang, S., Matena, M., Zhou, Y., Li, W., and Liu, P.J. (2020). Exploring the limits of transfer learning with a unified text-to-text transformer. J. Mach. Learn. Res. *21*, 5485–5551.

55. Xue, L., Constant, N., Roberts, A., Kale, M., Al-Rfou, R., Siddhant, A., Barua, A., and Raffel, C. (2021). mt5: A massively multilingual pre-trained text-to-text transformer. In Proceedings of the 2021 Conference of the North American Chapter of the Association for Computational Linguistics: Human Language Technologies, pp. 483–498.

56. Lewis, M., Liu, Y., Goyal, N., Ghazvininejad, M., Mohamed, A., Levy, O., Stoyanov, V., and Zettlemoyer, L. (2019). Bart: Denoising sequence-to-sequence pre-training for natural language generation, translation, and comprehension. Preprint at arXiv. https://doi.org/10.48550/arXiv.1910.13461.

57. Wei, J., Bosma, M., Zhao, V.Y., Guu, K., Yu, A.W., Lester, B., Du, N., Dai, A.M., and Le, Q.V. (2021). Finetuned language models are zero-shot learners. Preprint at arXiv. https://doi.org/10.48550/arXiv.2109.01652.

58. Rae, J.W., Borgeaud, S., Cai, T., Millican, K., Hoffmann, J., Song, F., Aslanides, J., Henderson, S., Ring, R., Young, S., et al. (2021). Scaling language models: Methods, analysis & insights from training gopher. Preprint at arXiv. https://doi.org/10.48550/arXiv.2112.11446.

59. Sanh, V., Webson, A., Raffel, C., Bach, S.H., Sutawika, L., Alyafeai, Z., Chaffin, A., Stiegler, A., Scao, T.L., Raja, A., et al. (2021). Multitask prompted training enables zero-shot task generalization. Preprint at arXiv. https://doi.org/10.48550/arXiv.2110.08207.

60. Du, N., Huang, Y., Dai, A.M., Tong, S., Lepikhin, D., Xu, Y., Krikun, M., Zhou, Y., Yu, A.W., Firat, O., et al. (2022). Glam: Efficient scaling of language models with mixture-of-experts. In International Conference on Machine Learning (PMLR), pp. 5547–5569.

61. Giarelis, N., Mastrokostas, C., Siachos, I., and Karacapilidis, N. (2023). A review of Greek NLP technologies for chatbot development. In Proceedings of the 27th Pan-Hellenic Conference on Progress in Computing and Informatics, pp. 15–20.

62. Nikiforos, M.N., Voutos, Y., Drougani, A., Mylonas, P., and Kermanidis, K. L. (2021). The modern Greek language on the social web: A survey of data sets and mining applications. Data *6*, 52. https://doi.org/10.3390/data6050052.

63. Alexandridis, G., Varlamis, I., Korovesis, K., Caridakis, G., and Tsantilas, P. (2021). A survey on sentiment analysis and opinion mining in Greek social media. Information *12*, 331. https://doi.org/10.3390/info12080331.

64. Krasadakis, P., Sakkopoulos, E., and Verykios, V.S. (2022). A natural language processing survey on legislative and Greek documents. In 25th Pan-Hellenic Conference on Informatics. PCI 2021 (Association for Computing Machinery), pp. 407–412. https://doi.org/10.1145/3503823.3503898.

65. Association for Computational Linguistics. ACL anthology. https://aclanthology.org/.

66. Allen Institute for AI. Semantic scholar. https://www.semanticscholar.org/.

67. Elsevier. Scopus. https://www.scopus.com/home.uri.

68. ACL. Data and software for building the ACL anthology GitHub. https://github.com/acl-org/acl-anthology.

69. Google. Google scholar. https://scholar.google.com/.

70. Bommasani, R., Liang, P., and Lee, T. (2023). Holistic evaluation of language models. Ann. N. Y. Acad. Sci. *1525*, 140–146.

71. Association for Computational Linguistics. ACL 2023 main conference call for papers. https://2023.aclweb.org/calls/main_conference/.

72. Wilkinson, M.D., Dumontier, M., Aalbersberg, I.J.J., Appleton, G., Axton, M., Baak, A., Blomberg, N., Boiten, J.W., da Silva Santos, L.B., Bourne, P.E., et al. (2016). The FAIR Guiding Principles for scientific data management and stewardship. Sci. Data *3*, 160018. https://doi.org/10.1038/sdata.2016.18.

73. Belinkov, Y., Gehrmann, S., and Pavlick, E. (2020). Interpretability and analysis in neural NLP. In Proceedings of the 58th annual meeting of the association for computational linguistics: tutorial abstracts, pp. 1–5.

74. Prokopidis, P., and Piperidis, S. (2020). A neural NLP toolkit for Greek. In 11th Hellenic Conference on Artificial Intelligence, pp. 125–128. https://doi.org/10.1145/3411408.3411430.

75. Bojanowski, P., Grave, E., Joulin, A., and Mikolov, T. (2017). Enriching word vectors with subword information. Trans. Assoc. Comput. Linguist. *5*, 135–146. https://doi.org/10.1162/tacl_a_00051.

76. Tsakalidis, A., Papadopoulos, S., Voskaki, R., Ioannidou, K., Boididou, C., Cristea, A.I., Liakata, M., and Kompatsiaris, Y. (2018). Building and evaluating resources for sentiment analysis in the Greek language. Lang. Resour. Eval. *52*, 1021–1044. https://doi.org/10.1007/s10579-018-9420-4.

77. Mikolov, T., Sutskever, I., Chen, K., Corrado, G.S., and Dean, J. (2013). Distributed representations of words and phrases and their compositionality. In Advances in Neural Information Processing Systems, *26*, C. Burges, L. Bottou, M. Welling, Z. Ghahramani, and K. Weinberger, eds. *(Curran Associates, Inc.), pp. 3111–3119.* https://proceedings.neurips.cc/paper_files/paper/2013/file/9aa42b31882ec039965f3c4923ce901b-Paper.pdf.

CellPress
OPEN ACCESS

78. Pavlopoulos, J., Malakasiotis, P., and Androutsopoulos, I. (2017). Deep learning for user comment moderation. Preprint at arXiv. https://doi.org/10.48550/arXiv.1705.09993.
79. Pavlopoulos, J., Malakasiotis, P., Bakagianni, J., and Androutsopoulos, I. (2017). Improved abusive comment moderation with user embeddings. Preprint at arXiv. https://doi.org/10.48550/arXiv.1708.03699.
80. Medrouk, L., and Pappa, A. (2017). Deep learning model for sentiment analysis in multi-lingual corpus. In Neural Information Processing, D. Liu, S. Xie, Y. Li, D. Zhao, and E.S.M. El-Alfy, eds. (Springer International Publishing), pp. 205–212. https://doi.org/10.1007/978-3-319-70087-8_22.
81. Pitenis, Z., Zampieri, M., and Ranasinghe, T. (2020). Offensive language identification in Greek. Preprint at arXiv. https://doi.org/10.48550/arXiv.2003.07459.
82. Arcila-Calderón, C., Amores, J.J., Sánchez-Holgado, P., Vrysis, L., Vryzas, N., and Oller Alonso, M. (2022). How to detect online hate towards migrants and refugees? developing and evaluating a classifier of racist and xenophobic hate speech using shallow and deep learning. Sustainability *14*, 13094. https://doi.org/10.3390/su142013094.
83. Perifanos, K., and Goutsos, D. (2021). Multimodal hate speech detection in Greek social media. Multimodal Technol. Interact. *5*, 34. https://doi.org/10.3390/mti5070034.
84. Alexandridis, G., Korovesis, K., Varlamis, I., Tsantilas, P., and Caridakis, G. (2022). Emotion detection on Greek social media using bidirectional encoder representations from transformers. In 25th Pan-Hellenic Conference on Informatics. PCI 2021 (Association for Computing Machinery), pp. 28–32. https://doi.org/10.1145/3503823.3503829.
85. Rizou, S., Paflioti, A., Theofilatos, A., Vakali, A., Sarigiannidis, G., and Chatzisavvas, K.C. (2022). Multilingual name entity recognition and intent classification employing deep learning architectures. Simulat. Model. Pract. Theor. *120*, 102620. https://doi.org/10.1016/j.simpat.2022.102620.
86. Bilianos, D. (2022). Experiments in text classification: Analyzing the sentiment of electronic product reviews in Greek. J. Quant. Ling. *29*, 374–386. https://doi.org/10.1080/09296174.2021.1885872.
87. Kapoteli, E., Koukaras, P., and Tjortjis, C. (2022). Social media sentiment analysis related to covid-19 vaccines: Case studies in English and Greek language. In Artificial Intelligence Applications and Innovations, I. Maglogiannis, L. Iliadis, J. Macintyre, and P. Cortez, eds. (Springer International Publishing), pp. 360–372. https://doi.org/10.1007/978-3-031-08337-2_30.
88. Wikimedia Foundation. Greek wikipedia dumps. https://dumps.wikimedia.org/elwiki/.
89. Koehn, P. (2005). Europarl: A parallel corpus for statistical machine translation. In Proceedings of machine translation summit x: papers, pp. 79–86.
90. Suárez, P.J.O., Sagot, B., and Romary, L. (2019). Asynchronous pipeline for processing huge corpora on medium to low resource infrastructures. In 7th Workshop on the Challenges in the Management of Large Corpora (CMLC-7) (Leibniz-Institut für Deutsche Sprache), pp. 9–16. https://doi.org/10.14618/IDS-PUB-9021.
91. Common Crawl Foundation (2025). Common crawl. https://commoncrawl.org/.
92. Zaikis, D., Stylianou, N., and Vlahavas, I. (2023). Pima: Parameter-shared intelligent media analytics framework for low resource languages. Appl. Sci. *13*, 3265. https://doi.org/10.3390/app13053265.
93. Lewis, M., Liu, Y., Goyal, N., Ghazvininejad, M., Mohamed, A., Levy, O., Stoyanov, V., and Zettlemoyer, L. (2020). Bart: Denoising sequence-to-sequence pre-training for natural language generation, translation, and comprehension. In Proceedings of the 58th Annual Meeting of the Association for Computational Linguistics, pp. 7871–7880.
94. Outsios, S., Skianis, K., Meladianos, P., Xypolopoulos, C., and Vazirgiannis, M. (2018). Word embeddings from large-scale Greek web content. Preprint at arXiv. https://doi.org/10.48550/arXiv.1810.06694.
95. Giarelis, N., Mastrokostas, C., and Karacapilidis, N. (2024). Greekt5: Sequence-to-sequence models for Greek news summarization. In IFIP International Conference on Artificial Intelligence Applications and Innovations (Springer), pp. 60–73. https://doi.org/10.1007/978-3-031-63215-0_5.
96. Chung, H.W., Garcia, X., Roberts, A., Tay, Y., Firat, O., Narang, S., and Constant, N. (2024). Unimax: Fairer and more effective language sampling for large-scale multilingual pretraining. In The Eleventh International Conference on Learning Representations.
97. Giarelis, N., Mastrokostas, C., and Karacapilidis, N. (2024). Greekt5-mt5-small-greeksum. Hugging Face. https://huggingface.co/IMISLab/GreekT5-mt5-small-greeksum.
98. Giarelis, N., Mastrokostas, C., and Karacapilidis, N. (2024). Greekt5-umt5-small-greeksum. Hugging Face. https://huggingface.co/IMISLab/GreekT5-umt5-small-greeksum.
99. Giarelis, N., Mastrokostas, C., and Karacapilidis, N. (2024). Greekt5-umt5-base-greeksum. Hugging Face. https://huggingface.co/IMISLab/GreekT5-umt5-base-greeksum.
100. Evdaimon, I., Abdine, H., Xypolopoulos, C., Outsios, S., Vazirgiannis, M., and Stamou, G. (2023). Greekbart: The first pretrained Greek sequence-to-sequence model. GitHub. https://github.com/iakovosevdaimon/GreekBART.
101. Koutsikakis, J., Chalkidis, I., Malakasiotis, P., and Androutsopoulos, I. (2020). Greekbert: bert-base-greek-uncased-v1. Hugging Face. https://huggingface.co/nlpaueb/bert-base-greek-uncased-v1.
102. Zaikis, D., Stylianou, N., and Vlahavas, I. (2021). Greek media bert base uncased. Hugging Face. https://huggingface.co/dimitriz/greek-media-bert-base-uncased.
103. Alexandridis, G., Varlamis, I., Korovesis, K., Caridakis, G., and Tsantilas, P. (2021). Greeksocialbert base greek uncased v1. Hugging Face. https://huggingface.co/gealexandri/greeksocialbert-base-greek-uncased-v1.
104. Alexandridis, G., Varlamis, I., Korovesis, K., Caridakis, G., and Tsantilas, P. (2021). Palobert base greek uncased v1. Hugging Face. https://huggingface.co/gealexandri/palobert-base-greek-uncased-v1.
105. Perifanos, K., and Goutsos, D. (2021). Bertatweetgr. Hugging Face. https://huggingface.co/Konstantinos/BERTaTweetGR.
106. Papadimitriou, I., Lopez, K., and Jurafsky, D. (2023). Multilingual bert has an accent: Evaluating English influences on fluency in multilingual models. In Findings of the Association for Computational Linguistics: EACL 2023, pp. 1194–1200.
107. Ahn, J., and Oh, A. (2021). Mitigating language-dependent ethnic bias in bert. Preprint at arXiv. https://doi.org/10.48550/arXiv.2109.05704.
108. Garí Soler, A., and Apidianaki, M. (2021). Let's play mono-poly: Bert can reveal words' polysemy level and partitionability into senses. Trans. Assoc. Comput. Linguist. *9*, 825–844. https://doi.org/10.1162/tacl_a_00400.
109. Garí Soler, A., and Apidianaki, M. (2021). Let's play mono-poly. GitHub. https://github.com/ainagari/monopoly.
110. Gonen, H., Ravfogel, S., Elazar, Y., and Goldberg, Y. (2020). It's not Greek to mbert: Inducing word-level translations from multilingual BERT. Preprint at arXiv. https://doi.org/10.48550/arXiv.2010.08275.
111. Gonen, H., Ravfogel, S., Elazar, Y., and Goldberg, Y. (2020). It's not Greek to mbert: Inducing word-level translations from multilingual bert. GitHub. https://github.com/gonenhila/mbert.
112. Balloccu, S., Schmidtová, P., Lango, M., and Dusek, O. (2024). Leak, Cheat, Repeat: Data Contamination and Evaluation Malpractices in Closed-Source LLMs. In Proceedings of the 18th Conference of the European Chapter of the Association for Computational Linguistics (Volume 1: Long Papers), Y. Graham and M. Purver, eds. (Association for Computational Linguistics), pp. 67–93.
113. Achiam, J., Adler, S., Agarwal, S., Ahmad, L., Akkaya, I., Aleman, F.L., Almeida, D., Altenschmidt, J., Altman, S., Anadkat, S., et al. (2023). Gpt-4 technical report. Preprint at arXiv. https://doi.org/10.48550/arXiv.2303.08774.

114. Chung, H.W., Hou, L., Longpre, S., Zoph, B., Tay, Y., Fedus, W., Li, Y., Wang, X., Dehghani, M., Brahma, S., et al. (2024). Scaling instruction-finetuned language models. J. Mach. Learn. Res. *25*, 1–53. https://doi.org/10.5555/3722577.3722647.

115. Loukas, L., Smyrnioudis, N., Dikonomaki, C., Barbakos, S., Toumazatos, A., Koutsikakis, J., Kyriakakis, M., Georgiou, M., Vassos, S., Pavlopoulos, J., and Androutsopoulos, I. (2025). GR-NLP-TOOLKIT: An open-source NLP toolkit for Modern Greek. In Proceedings of the 31st International Conference on Computational Linguistics: System Demonstrations, O. Rambow, L. Wanner, M. Apidianaki, H. Al-Khalifa, B.D. Eugenio, S. Schockaert, B. Mather, and M. Dras, eds. (Association for Computational Linguistics), pp. 174–182.

116. Voukoutis, L., Roussis, D., Paraskevopoulos, G., Sofianopoulos, S., Prokopidis, P., Papavasileiou, V., Katsamanis, A., Piperidis, S., and Katsouros, V. (2024). Meltemi: The first open large language model for Greek. Preprint at arXiv. https://doi.org/10.48550/arXiv.2407.20743.

117. Institute for Language and Speech Processing (ILSP) (2024). Llama-krikri-8b-base. Hugging Face. https://huggingface.co/ilsp/Llama-Krikri-8B-Base.

118. Liang, Y., Duan, N., Gong, Y., Wu, N., Guo, F., Qi, W., Gong, M., Shou, L., Jiang, D., Cao, G., et al. (2020). Xglue: A new benchmark dataset for cross-lingual pre-training, understanding and generation. In Proceedings of the 2020 Conference on Empirical Methods in Natural Language Processing (EMNLP), pp. 6008–6018.

119. Hu, J., Ruder, S., Siddhant, A., Neubig, G., Firat, O., and Johnson, M. (2020). Xtreme: A massively multilingual multi-task benchmark for evaluating cross-lingual generalisation. In International conference on machine learning (PMLR), pp. 4411–4421.

120. Woolf, B.P. (2010). Building Intelligent Interactive Tutors: Student-Centered Strategies for Revolutionizing E-Learning (Morgan Kaufmann).

121. Cambria, E., Poria, S., Gelbukh, A., and Thelwall, M. (2017). Sentiment analysis is a big suitcase. IEEE Intell. Syst. *32*, 74–80. https://doi.org/10.1109/MIS.2017.4531228.

122. Zhang, X., Mao, R., and Cambria, E. (2023). A survey on syntactic processing techniques. Artif. Intell. Rev. *56*, 5645–5728. https://doi.org/10.1007/s10462-022-10300-7.

123. Wang, Y., Wang, Y., Dang, K., Liu, J., and Liu, Z. (2021). A comprehensive survey of grammatical error correction. ACM Trans. Intell. Syst. Technol. *12*, 1–51. https://doi.org/10.1145/3474840.

124. Kiss, T., and Strunk, J. (2006). Unsupervised multilingual sentence boundary detection. Comput. Linguist. *32*, 485–525. https://doi.org/10.1162/coli.2006.32.4.485.

125. Qi, P., Dozat, T., Zhang, Y., and Manning, C.D. (2019). Universal dependency parsing from scratch. Preprint at arXiv. https://doi.org/10.48550/arXiv.1901.10457.

126. Dozat, T., and Manning, C.D. (2016). Deep biaffine attention for neural dependency parsing. Preprint at arXiv. https://doi.org/10.48550/arXiv.1611.01734.

127. Prokopidis, P., and Papageorgiou, H. (2017). Universal dependencies for Greek. In Proceedings of the nodalida 2017 workshop on universal dependencies (udw 2017), pp. 102–106.

128. Partalidou, E., Spyromitros-Xioufis, E., Doropoulos, S., Vologiannidis, S., and Diamantaras, K. (2019). Design and implementation of an open source Greek pos tagger and entity recognizer using spacy. In IEEE/WIC/ACM International Conference on Web Intelligence. WI '19 IEEE (Association for Computing Machinery), pp. 337–341. https://doi.org/10.1145/3350546.3352543.

129. Honnibal, M., and Montani, I. (2017). spacy 2: Natural language understanding with bloom embeddings, convolutional neural networks and incremental parsing. Sentometrics Research. https://sentometrics-research.com/publication/72/.

130. Prokopidis, P., Georgantopoulos, B., and Papageorgiou, H. (2011). A suite of natural language processing tools for Greek. In The 10th international conference of Greek linguistics.

131. Bird, S. (2006). Nltk: the natural language toolkit. In Proceedings of the COLING/ACL 2006 Interactive Presentation Sessions, pp. 69–72.

132. Al-Rfou, R. (2015). Polyglot: Natural language pipeline supporting massive multilingual applications. GitHub. https://github.com/aboSamoor/polyglot.

133. Explosion AI (2025). spacy Greek language models. spaCy. https://spacy.io/models/el.

134. GFOSS - Open Technologies Alliance (2018). Greek language support for spacy (gsoc 2018 project). GitHub. https://github.com/eellak/gsoc2018-spacy.

135. Qi, P., Zhang, Y., Zhang, Y., Bolton, J., and Manning, C.D. (2020). Stanza: A python natural language processing toolkit for many human languages. Preprint at arXiv. https://doi.org/10.48550/arXiv.2003.07082.

136. Straka, M., and Straková, J. (2017). Tokenizing, pos tagging, lemmatizing and parsing ud 2.0 with udpipe. In Proceedings of the CoNLL 2017 shared task: Multilingual parsing from raw text to universal dependencies, pp. 88–99.

137. Zuhra, F.T., and Saleem, K. (2023). Hybrid embeddings for transition-based dependency parsing of free word order languages. Inf. Process. Manag. *60*, 103334. https://doi.org/10.1016/j.ipm.2023.103334.

138. Wong, D.F., Chao, L.S., and Zeng, X. (2014). isentenizer-: Multilingual sentence boundary detection model. Sci. World J. *2014*, 196574. https://doi.org/10.1155/2014/196574.

139. Fotopoulou, A., and Giouli, V. (2015). Mwes: support/light verb constructions vs fixed expressions in modern Greek and french. In Workshop on Multiword units in machine translation and translation technology. Tradulex, pp. 68–73.

140. Samaridi, N., and Markantonatou, S. (2014). Parsing Modern Greek verb MWEs with LFG/XLE grammars. In Proceedings of the 10th Workshop on Multiword Expressions (MWE) (Association for Computational Linguistics), pp. 33–37.

141. Korre, K., Chatzipanagiotou, M., and Pavlopoulos, J. (2021). Elerrant: Automatic grammatical error type classification for Greek. In Proceedings of the International Conference on Recent Advances in Natural Language Processing (RANLP 2021), pp. 708–717.

142. Gakis, P., Panagiotakopoulos, C., Sgarbas, K., Tsalidis, C., and Verykios, V. (2016). Design and construction of the Greek grammar checker. Digital Scholarship Humanities *32*, fqw025. https://doi.org/10.1093/llc/fqw025.

143. Neurolingo L.P. (2017). Grammar checker. http://www.neurolingo.gr/en/online_tools/ggc.

144. Kavros, A., and Tzitzikas, Y. (2022). Soundexgr: An algorithm for phonetic matching for the Greek language. Nat. Lang. Eng. *29*, 1305–1340. https://doi.org/10.1017/S1351324922000018.

145. Kavros, A., and Tzitzikas, Y. (2022). Soundexgr: An algorithm for phonetic matching for the Greek language. GitHub. https://github.com/YannisTzitzikas/SoundexGR.

146. Hua, Y., Danescu-Niculescu-Mizil, C., Taraborelli, D., Thain, N., Sorensen, J., and Dixon, L. (2018). WikiConv: A corpus of the complete conversational history of a large online collaborative community. In Proceedings of the 2018 Conference on Empirical Methods in Natural Language Processing (Association for Computational Linguistics), pp. 2818–2823.

147. Korre, K., Chatzipanagiotou, M., and Pavlopoulos, J. (2021). Elerrant: Greek version of errant. GitHub. https://github.com/katkorre/elerrant.

148. Prokopidis, P., and Papageorgiou, H. (2017). Ud_greek-gdt: Universal dependencies treebank. GitHub. https://github.com/UniversalDependencies/UD_Greek-GDT.

149. Gakis, P., Panagiotakopoulos, C., Sgarbas, K., and Tsalidis, C. (2015). Analysis of lexical ambiguity in modern Greek using a computational lexicon. Digital Scholarship in the Humanities *30*, 20–38. https://doi.org/10.1093/llc/fqt035.

150. Nivre, J., De Marneffe, M.C., Ginter, F., Goldberg, Y., Hajic, J., Manning, C.D., McDonald, R., Petrov, S., Pyysalo, S., Silveira, N., et al. (2016). Universal dependencies v1: A multilingual treebank collection. In

CellPress
OPEN ACCESS

Proceedings of the Tenth International Conference on Language Resources and Evaluation (LREC'16), pp. 1659–1666.

151. Prokopidis, P., Desipri, E., Koutsombogera, M., Papageorgiou, H., and Piperidis, S. (2005). Theoretical and practical issues in the construction of a Greek dependency corpus. In Proceedings of the Fourth Workshop on Treebanks and Linguistic Theories:(TLT 2005): 9-10 december 2005, Barcelona. Publicaciones y Ediciones= Publicacions i Edicions, pp. 149–160.

152. Gakis, P., Panagiotakopoulos, C., Sgarbas, K., and Tsalidis, C. (2012). Design and implementation of an electronic lexicon for modern Greek. Lit. Ling. Comput. *27*, 155–169. https://doi.org/10.1093/llc/fqs002.

153. Goddard, C. (2011). Semantic Analysis: A Practical Introduction (Oxford University Press).

154. Cruse, D.A. (1986). Lexical Semantics (Cambridge university press).

155. Prasad, R., Dinesh, N., Lee, A., Miltsakaki, E., Robaldo, L., Joshi, A.K., and Webber, B.L. (2008). The penn discourse treebank 2.0. In Proceedings of the Sixth International Language Resources and Evaluation (LREC'08) (European Language Resources Association (ELRA)), pp. 2961–2968.

156. Lenci, A., Sahlgren, M., Jeuniaux, P., Cuba Gyllensten, A., and Miliani, M. (2022). A comparative evaluation and analysis of three generations of distributional semantic models. Lang. Resour. Eval. *56*, 1269–1313. https://doi.org/10.1007/s10579-021-09575-z.

157. Harris, Z.S. (1954). Distributional structure. Word *10*, 146–162. https://doi.org/10.1080/00437956.1954.11659520.

158. Sahlgren, M. (2008). The distributional hypothesis. Italian Journal of Linguistics *20*, 33–53.

159. Zervanou, K., Iosif, E., and Potamianos, A. (2014). Word semantic similarity for morphologically rich languages. In LREC, pp. 1642–1648.

160. Palogiannidi, E., Losif, E., Koutsakis, P., and Potamianos, A. (2015). Valence, arousal and dominance estimation for English, German, Greek, Portuguese and Spanish lexica using semantic models. In INTERSPEECH 2015, pp. 1527–1531.

161. Palogiannidi, E., Koutsakis, P., Iosif, E., and Potamianos, A. (2016). Affective lexicon creation for the Greek language. In Proceedings of the Tenth International Conference on Language Resources and Evaluation (LREC'16) (European Language Resources Association (ELRA)), pp. 2867–2872.

162. Iosif, E., Georgiladakis, S., and Potamianos, A. (2016). Cognitively motivated distributional representations of meaning. In Proceedings of the Tenth International Conference on Language Resources and Evaluation (LREC'16) (European Language Resources Association (ELRA)), pp. 1226–1232.

163. Kahneman, D. (2011). Thinking, Fast and Slow, *499* (Farrar, Straus and Giroux).

164. Lioudakis, M., Outsios, S., and Vazirgiannis, M. (2019). An ensemble method for producing word representations for the Greek language. Preprint at arXiv. https://doi.org/10.18653/v1/2020.loresmt-1.13.

165. Lioudakis, M., Outsios, S., and Vazirgiannis, M. (2019). An ensemble method for producing word representations for the Greek language. GitHub. https://github.com/mikeliou/greek_word_embeddings.

166. Outsios, S., Karatsalos, C., Skianis, K., and Vazirgiannis, M. (2020). Evaluation of Greek word embeddings. In Proceedings of the Twelfth Language Resources and Evaluation Conference (European Language Resources Association), pp. 2543–2551.

167. Dritsa, K., Thoma, A., Pavlopoulos, I., and Louridas, P. (2022). A Greek parliament proceedings dataset for computational linguistics and political analysis. In Advances in Neural Information Processing Systems, *35*, S. Koyejo, S. Mohamed, A. Agarwal, D. Belgrave, K. Cho, and A. Oh, eds. *(Curran Associates, Inc.), pp. 28874–28888.* https://proceedings.neurips.cc/paper_files/paper/2022/file/b96ce67b2f2d45e4ab315e13a6b5b9c5-Paper-Datasets_and_Benchmarks.pdf.

168. Barzokas, V., Papagiannopoulou, E., and Tsoumakas, G. (2020). Studying the evolution of Greek words via word embeddings. In 11th Hellenic Conference on Artificial Intelligence. SETN 2020 (Association for Computing Machinery), pp. 118–124. https://doi.org/10.1145/3411408.3411425.

169. Hamilton, W.L., Leskovec, J., and Jurafsky, D. (2016). Diachronic word embeddings reveal statistical laws of semantic change. In Proceedings of the 54th Annual Meeting of the Association for Computational Linguistics (Volume 1: Long Papers), pp. 1489–1501.

170. Di Carlo, V., Bianchi, F., and Palmonari, M. (2019). Training temporal word embeddings with a compass. In Proceedings of the AAAI Conference on Artificial Intelligence vol. 33. pp. 6326–6334.

171. Gonen, H., Jawahar, G., Seddah, D., and Goldberg, Y. (2020). Simple, interpretable and stable method for detecting words with usage change across corpora. In Proceedings of the 58th Annual Meeting of the Association for Computational Linguistics, D. Jurafsky, J. Chai, N. Schluter, and J. Tetreault, eds. (Association for Computational Linguistics), pp. 538–555. https://doi.org/10.18653/v1/2020.acl-main.51.

172. Hamilton, W.L., Leskovec, J., and Jurafsky, D. (2016). Cultural shift or linguistic drift? comparing two computational measures of semantic change. In Proceedings of the Conference on Empirical Methods in Natural Language Processing. vol. 2016. pp. 2116–2121.

173. Florou, E., Perifanos, K., and Goutsos, D. (2018). Neural embeddings for metaphor detection in a corpus of Greek texts. In 2018 9th International Conference on Information, Intelligence, Systems and Applications (IISA) (IEEE), pp. 1–4. https://doi.org/10.1109/IISA.2018.8633668.

174. Steen, G. (2007). Finding Metaphor in Grammar and Usage: A Methodological Analysis of Theory and Research. In Converging evidence in language and communication research (J. Benjamins Publishing Company).

175. Chowdhury, S.A., Ghosh, A., Stepanov, E.A., Bayer, A.O., Riccardi, G., and Klasinas, I. (2014). Cross-language transfer of semantic annotation via targeted crowdsourcing. In INTERSPEECH, pp. 2108–2112. https://doi.org/10.21437/Interspeech.2014-478.

176. Kamath, A., and Das, R. (2018). A survey on semantic parsing. Preprint at arXiv. https://doi.org/10.48550/arXiv.1812.00978.

177. Li, J., Zhu, M., Lu, W., and Zhou, G. (2015). Improving semantic parsing with enriched synchronous context-free grammar. In Proceedings of the 2015 Conference on Empirical Methods in Natural Language Processing, pp. 1455–1465.

178. Conneau, A., Rinott, R., Lample, G., Williams, A., Bowman, S.R., Schwenk, H., and Stoyanov, V. (2018). Xnli: Evaluating cross-lingual sentence representations. In Proceedings of the 2018 Conference on Empirical Methods in Natural Language Processing (Association for Computational Linguistics), pp. 2475–2485.

179. Parikh, A., Täckström, O., Das, D., and Uszkoreit, J. (2016). A decomposable attention model for natural language inference. In Proceedings of the 2016 Conference on Empirical Methods in Natural Language Processing, pp. 2249–2255.

180. Giachos, I., Papakitsos, E.C., Antonopoulos, I., and Laskaris, N. (2023). Systemic and hole semantics in human-machine language interfaces. In 2023 17th International Conference on Engineering of Modern Electric Systems (EMES) (IEEE), pp. 1–4. https://doi.org/10.1109/EMES58375.2023.10171635.

181. Ganitkevitch, J., and Callison-Burch, C. (2014). The multilingual paraphrase database. In LREC, pp. 4276–4283. Citeseer.

182. Ganitkevitch, J., and Callison-Burch, C. (2013). The paraphrase database. http://paraphrase.org/.

183. Outsios, S., Karatsalos, C., Skianis, K., and Vazirgiannis, M. (2020). Wordsim353 dataset translated. http://archive.aueb.gr:7000/resources/.

184. Outsios, S., Karatsalos, C., Skianis, K., and Vazirgiannis, M. (2020). Greek word analogy test set. http://archive.aueb.gr:7000/resources/.

185. Pilitsidou, V., and Giouli, V. (2021). Frame semantics in the specialized domain of finance: Building a termbase to aid translation. In Lexicography for Inclusion: Proceedings of the 19th EURALEX

International Congress, 7–9 September 2021, Alexandroupolis, *1*, Z. Gavriilidou, M. Mitsiaki, and A. Fliatouras, eds. *(Democritus University of Thrace), pp. 263–271.* https://euralex.org/publications/frame-semantics-in-the-specialized-domain-of-finance-building-a-termbase-to-translation/.

186. Giouli, V., Pilitsidou, V., and Christopoulos, H. (2020). Greek within the global FrameNet initiative: Challenges and conclusions so far. In Proceedings of the International FrameNet Workshop 2020: Towards a Global, Multilingual FrameNet (European Language Resources Association), pp. 48–55.
187. Ganitkevitch, J., Van Durme, B., and Callison-Burch, C. (2013). Ppdb: The paraphrase database. In Proceedings of the 2013 Conference of the North American Chapter of the Association for Computational Linguistics: Human Language Technologies, pp. 758–764.
188. Bovi, C.D., Camacho-Collados, J., Raganato, A., and Navigli, R. (2017). Eurosense: Automatic harvesting of multilingual sense annotations from parallel text. In Proceedings of the 55th Annual Meeting of the Association for Computational Linguistics (Volume 2: Short Papers), pp. 594–600.
189. Navigli, R., and Ponzetto, S.P. (2012). Babelnet: The automatic construction, evaluation and application of a wide-coverage multilingual semantic network. Artif. Intell. *193*, 217–250. https://doi.org/10.1016/j.artint.2012.07.001.
190. Finkelstein, L., Gabrilovich, E., Matias, Y., Rivlin, E., Solan, Z., Wolfman, G., and Ruppin, E. (2001). Placing search in context: The concept revisited. In Proceedings of the 10th international conference on World Wide Web, pp. 406–414.
191. Mikolov, T., Chen, K., Corrado, G., and Dean, J. (2013). Efficient estimation of word representations in vector space. Preprint at arXiv. https://doi.org/10.48550/arXiv.1301.3781.
192. Nikolaev, D., and Padó, S. (2023). The universe of utterances according to bert. In Proceedings of the 15th International Conference on Computational Semantics, pp. 99–105.
193. Nikolaev, D., and Padó, S. (2023). Investigating semantic subspaces of transformer sentence embeddings through linear structural probing. In Proceedings of the 6th BlackboxNLP Workshop: Analyzing and Interpreting Neural Networks for NLP, Y. Belinkov, S. Hao, J. Jumelet, N. Kim, A. McCarthy, and H. Mohebbi, eds. (Association for Computational Linguistics), pp. 142–154. https://doi.org/10.18653/v1/2023.blackboxnlp-1.11.
194. Yang, Y., Wu, Z., Yang, Y., Lian, S., Guo, F., and Wang, Z. (2022). A survey of information extraction based on deep learning. Appl. Sci. *12*, 9691. https://doi.org/10.3390/app12199691.
195. Mouratidis, D., Kermanidis, K., and Kanavos, A. (2023). Comparative study of recurrent and dense neural networks for classifying maritime terms. In 2023 14th International Conference on Information, Intelligence, Systems & Applications (IISA) (IEEE), pp. 1–6. https://doi.org/10.1109/IISA59645.2023.10345925.
196. Mouratidis, D., Mathe, E., Voutos, Y., Stamou, K., Kermanidis, K.L., Mylonas, P., and Kanavos, A. (2022). Domain-specific term extraction: A case study on Greek maritime legal texts. In Proceedings of the 12th Hellenic Conference on Artificial Intelligence. SETN '22 (Association for Computing Machinery), pp. 1–6.
197. Papadopoulos, D., Papadakis, N., and Matsatsinis, N. (2021). Penelopie: Enabling open information extraction for the Greek language through machine translation. GitHub. https://github.com/lighteternal/PENELOPIE.
198. Barbaresi, A., and Lejeune, G. (2020). Out-of-the-box and into the ditch? multilingual evaluation of generic text extraction tools. In Proceedings of the 12th Web as Corpus Workshop (European Language Resources Association), pp. 5–13.
199. Lejeune, G., Brixtel, R., Doucet, A., and Lucas, N. (2015). Multilingual event extraction for epidemic detection. Artif. Intell. Med. *65*, 131–143. https://doi.org/10.1016/j.artmed.2015.06.005.
200. Brixtel, R., Lejeune, G., Doucet, A., and Lucas, N. (2013). Any language early detection of epidemic diseases from web news streams. In 2013 IEEE International Conference on Healthcare Informatics (IEEE), pp. 159–168. https://doi.org/10.1109/ICHI.2013.94.
201. Lejeune, G., Brixtel, R., Doucet, A., and Lucas, N. (2012). Daniel: Language independent character-based news surveillance. In International Conference on NLP (Springer), pp. 64–75. https://doi.org/10.1007/978-3-642-33983-7_7.
202. Papantoniou, K., Efthymiou, V., and Plexousakis, D. (2023). Automating benchmark generation for named entity recognition and entity linking. In European Semantic Web Conference (Springer), pp. 143–148. https://doi.org/10.1007/978-3-031-43458-7_27.
203. Rizou, S., Theofilatos, A., Paflioti, A., Pissari, E., Varlamis, I., Sarigiannidis, G., and Chatzisavvas, K.C. (2023). Efficient intent classification and entity recognition for university administrative services employing deep learning models. Intelligent Systems with Applications *19*, 200247. https://doi.org/10.1016/j.iswa.2023.200247.
204. Hemphill, C.T., Godfrey, J.J., and Doddington, G.R. (1990). The ATIS spoken language systems pilot corpus. In Speech and Natural Language: Proceedings of a Workshop Held at Hidden Valley, Pennsylvania, June 24-27,1990, pp. 96–101. https://doi.org/10.3115/116580.116613.
205. Bartziokas, N., Mavropoulos, T., and Kotropoulos, C. (2020). Datasets and performance metrics for Greek named entity recognition. In 11th Hellenic Conference on Artificial Intelligence. SETN 2020 (Association for Computing Machinery), pp. 160–167. https://doi.org/10.1145/3411408.3411437.
206. Sang, E.F., and De Meulder, F. (2003). Introduction to the conll-2003 shared task: Language-independent named entity recognition. Preprint at arXiv. https://doi.org/10.48550/arXiv.cs/0306050.
207. Pradhan, S.S., Hovy, E., Marcus, M., Palmer, M., Ramshaw, L., and Weischedel, R. (2007). Ontonotes: A unified relational semantic representation. In International Conference on Semantic Computing (ICSC 2007) (IEEE), pp. 517–526. https://doi.org/10.1109/ICSC.2007.83.
208. Angelidis, I., Chalkidis, I., and Koubarakis, M. (2018). Named entity recognition, linking and generation for Greek legislation. In JURIX, pp. 1–10. https://doi.org/10.3233/978-1-61499-935-5-1.
209. Papantoniou, K., Efthymiou, V., and Flouris, G. (2021). El-nel: Entity linking for Greek news articles. In ISWC (Posters/Demos/Industry).
210. Papantoniou, K., Efthymiou, V., and Plexousakis, D. (2022). Dataset for named entity recognition and entity linking from Greek wikipedia events. Zenodo. https://doi.org/10.5281/zenodo.7429037.
211. Rizou, S., Theofilatos, A., Paflioti, A., Pissari, E., Varlamis, I., Sarigiannidis, G., and Chatzisavvas, K.C. (2023). Uniway dataset. mSensis. https://msensis.com/wp-content/uploads/2023/06/uniway.zip.
212. Bartziokas, N., Mavropoulos, T., and Kotropoulos, C. (2021). elner: Greek named entity recognition. GitHub. https://github.com/nmpartzio/elNER.
213. Rizou, S., Paflioti, A., Theofilatos, A., Vakali, A., Sarigiannidis, G., and Chatzisavvas, K. (2022). Atis gr dataset. https://msensis.com/research-and-development/downloads.
214. Lioudakis, M., Outsios, S., and Vazirgiannis, M. (2019). Greek Ner Spacy Dataset (AUEB). http://archive.aueb.gr:7000/resources/.
215. Angelidis, I., Chalkidis, I., and Koubarakis, M. (2018). Publications – Legislation Mining Project (National and Kapodistrian University of Athens). https://legislation.di.uoa.gr/publications.
216. Lejeune, G., Brixtel, R., Doucet, A., and Lucas, N. (2012). Daniel: Language Independent Character-Based News Surveillance (Université de Caen Normandie). https://lejeuneg.users.greyc.fr/daniel/.
217. Explosion AI. Prodigy: An annotation tool for ai, machine learning & NLP. https://prodi.gy/.
218. Lawrence, J., and Reed, C. (2020). Argument mining: A survey. Comput. Linguist. *45*, 765–818. https://doi.org/10.1162/coli_a_00364.
219. Liu, B. (2020). Sentiment Analysis: Mining Opinions, Sentiments, and Emotions (Cambridge university press).

CellPress
OPEN ACCESS

220. Al-Ghuribi, S.M., and Noah, S.A.M. (2021). A comprehensive overview of recommender system and sentiment analysis. Preprint at arXiv. https://doi.org/10.48550/arXiv.2109.08794.

221. Rokade, P.P., and Aruna, K.D. (2019). Business intelligence analytics using sentiment analysis-a survey. Int. J. Electr. Comput. Eng. *9*, 613. https://doi.org/10.11591/ijece.v9i1.pp613-620.

222. Mudinas, A., Zhang, D., and Levene, M. (2019). Market trend prediction using sentiment analysis: lessons learned and paths forward. Preprint at arXiv. https://doi.org/10.48550/arXiv.1903.05440.

223. Chauhan, P., Sharma, N., and Sikka, G. (2021). The emergence of social media data and sentiment analysis in election prediction. J. Ambient Intell. Humaniz. Comput. *12*, 2601–2627. https://doi.org/10.1007/s12652-020-02423-y.

224. Turney, P.D. (2002). Thumbs up or thumbs down? semantic orientation applied to unsupervised classification of reviews. Preprint at arXiv. https://doi.org/10.48550/arXiv.cs/0212032.

225. Pang, B., Lee, L., and Vaithyanathan, S. (2002). Thumbs up? sentiment classification using machine learning techniques. Preprint at arXiv. https://doi.org/10.48550/arXiv.cs/0205070.

226. Fragkis, N. (2022). Greek movies dataset. Kaggle. https://www.kaggle.com/datasets/nikosfragkis/greek-movies-dataset.

227. Braoudaki, A., Kanellou, E., Kozanitis, C., and Fatourou, P. (2020). Hybrid data driven and rule based sentiment analysis on Greek text. Procedia Comput. Sci. *178*, 234–243. 9th International Young Scientists Conference in Computational Science, YSC2020, 05-12 September 2020. https://doi.org/10.1016/j.procs.2020.11.025.

228. Medrouk, L., and Pappa, A. (2018). Do deep networks really need complex modules for multilingual sentiment polarity detection and domain classification? In 2018 International Joint Conference on Neural Networks (IJCNN), pp. 1–6. https://doi.org/10.1109/IJCNN.2018.8489613.

229. Manias, G., Mavrogiorgou, A., Kiourtis, A., and Kyriazis, D. (2020). An evaluation of neural machine translation and pre-trained word embeddings in multilingual neural sentiment analysis. In 2020 IEEE International Conference on Progress in Informatics and Computing (PIC) (IEEE), pp. 274–283. https://doi.org/10.1109/PIC50277.2020.9350849.

230. Utathya (2018). Imdb review dataset. Kaggle. https://www.kaggle.com/utathya/imdb-review-dataset.

231. Markopoulos, G., Mikros, G., Iliadi, A., and Liontos, M. (2015). Sentiment analysis of hotel reviews in Greek: A comparison of unigram features. In Cultural Tourism in a Digital Era, V. Katsoni, ed. (Springer International Publishing), pp. 373–383.

232. Spatiotis, N., Mporas, I., Paraskevas, M., and Perikos, I. (2016). Sentiment analysis for the Greek language. In Proceedings of the 20th Pan-Hellenic Conference on Informatics. PCI '16 (Association for Computing Machinery), pp. 1–4.

233. Spatiotis, N., Paraskevas, M., Perikos, I., and Mporas, I. (2017). Examining the impact of feature selection on sentiment analysis for the Greek language. In Speech and Computer: 19th International Conference, SPECOM 2017, Hatfield, UK, September 12-16, 2017, Proceedings 19 (Springer), pp. 353–361. https://doi.org/10.1007/978-3-319-66429-3_34.

234. Spatiotis, N., Perikos, I., Mporas, I., and Paraskevas, M. (2019). Examining the impact of discretization technique on sentiment analysis for the Greek language. In 2019 10th International Conference on Information, Intelligence, Systems and Applications (IISA), pp. 1–6. https://doi.org/10.1109/IISA.2019.8900699.

235. Beleveslis, D., Tjortjis, C., Psaradelis, D., and Nikoglou, D. (2019). A hybrid method for sentiment analysis of election related tweets. In 2019 4th South-East Europe Design Automation, Computer Engineering, Computer Networks and Social Media Conference (SEEDA-CECNSM), pp. 1–6. https://doi.org/10.1109/SEEDA-CECNSM.2019.8908289.

236. Spatiotis, N., Perikos, I., Mporas, I., and Paraskevas, M. (2020). Sentiment analysis of teachers using social information in educational platform environments. Int. J. Artif. Intell. Tools *29*, 2040004. https://doi.org/10.1142/S0218213020400047.

237. Giatsoglou, M., Vozalis, M.G., Diamantaras, K., Vakali, A., Sarigiannidis, G., and Chatzisavvas, K.C. (2017). Sentiment analysis leveraging emotions and word embeddings. Expert Syst. Appl. *69*, 214–224. https://doi.org/10.1016/j.eswa.2016.10.043.

238. Patsiouras, E., Koroni, I., Mademlis, I., and Pitas, I. (2023). Greekpolitics: Sentiment analysis on Greek politically charged tweets. In 2023 31st European Signal Processing Conference (EUSIPCO) (IEEE), pp. 1320–1324. https://doi.org/10.23919/EUSIPCO58844.2023.10289909.

239. Katika, A., Zoulias, E., Koufi, V., and Malamateniou, F. (2023). Mining Greek tweets on long covid using sentiment analysis and topic modeling. In Healthcare Transformation with Informatics and Artificial Intelligence, J. Mantas, P. Gallos, E. Zoulias, A. Hasman, M.S. Househ, M. Charalampidou, and A. Magdalinou, eds. (IOS Press), pp. 545–548. https://doi.org/10.3233/SHTI230554.

240. Konstantinidis, N. (2023). Gpt-2 model for Greek. Hugging Face. https://huggingface.co/nikokons/gpt2-greek.

241. Drakopoulos, G., Giannoukou, I., Mylonas, P., and Sioutas, S. (2020). A graph neural network for assessing the affective coherence of twitter graphs. In 2020 IEEE International Conference on Big Data (Big Data) (IEEE), pp. 3618–3627. https://doi.org/10.1109/BigData50022.2020.9378492.

242. Charalampakis, B., Spathis, D., Kouslis, E., and Kermanidis, K. (2016). A comparison between semi-supervised and supervised text mining techniques on detecting irony in Greek political tweets. Eng. Appl. Artif. Intell. *51*, 50–57. Mining the Humanities: Technologies and Applications. https://doi.org/10.1016/j.engappai.2016.01.007.

243. Charalampakis, B., Spathis, D., Kouslis, E., and Kermanidis, K. (2015). Detecting irony on Greek political tweets: A text mining approach. In Proceedings of the 16th International Conference on Engineering Applications of Neural Networks (INNS). EANN '15 (Association for Computing Machinery), pp. 1–5.

244. Solakidis, G.S., Vavliakis, K.N., and Mitkas, P.A. (2014). Multilingual sentiment analysis using emoticons and keywords. In 2014 IEEE/WIC/ACM International Joint Conferences on Web Intelligence (WI) and Intelligent Agent Technologies (IAT), *2* (IEEE), pp. 102–109. https://doi.org/10.1109/WI-IAT.2014.86.

245. Chatzakou, D., Vakali, A., and Kafetsios, K. (2017). Detecting variation of emotions in online activities. Expert Syst. Appl. *89*, 318–332. https://doi.org/10.1016/j.eswa.2017.07.044.

246. Kydros, D., Argyropoulou, M., and Vrana, V. (2021). A content and sentiment analysis of Greek tweets during the pandemic. Sustainability *13*, 6150. https://doi.org/10.3390/su13116150.

247. Antonakaki, D., Spiliotopoulos, D., V. Samaras, C., Pratikakis, P., Ioannidis, S., and Fragopoulou, P. (2017). Social media analysis during political turbulence. PLoS One *12*, e0186836. https://doi.org/10.1371/journal.pone.0186836.

248. Antonakaki, D., Spiliotopoulos, D., Samaras, C.V., Ioannidis, S., and Fragopoulou, P. (2016). Investigating the complete corpus of referendum and elections tweets. In 2016 IEEE/ACM International Conference on Advances in Social Networks Analysis and Mining (ASONAM) (IEEE), pp. 100–105. https://doi.org/10.1109/ASONAM.2016.7752220.

249. Antonakaki, D., Spiliotopoulos, D., V. Samaras, C., Pratikakis, P., Ioannidis, S., and Fragopoulou, P. (2017). Elections study: Release 0.1. Zenodo. https://doi.org/10.5281/zenodo.820555.

250. Petasis, G., Spiliotopoulos, D., Tsirakis, N., and Tsantilas, P. (2014). Sentiment analysis for reputation management: Mining the Greek web. In Artificial Intelligence: Methods and Applications: 8th Hellenic Conference on AI, SETN 2014, Ioannina, Greece, May 15-17, 2014. Proceedings 8 (Springer), pp. 327–340. https://doi.org/10.1007/978-3-319-07064-3_26.

251. Tsakalidis, A., Aletras, N., Cristea, A.I., and Liakata, M. (2018). Nowcasting the stance of social media users in a sudden vote: The case of the Greek referendum. In Proceedings of the 27th ACM International Conference on Information and Knowledge Management. CIKM ’18 (Association for Computing Machinery), pp. 367–376. https://doi.org/10.1145/3269206.3271783.

252. Sliwa, A., Ma, Y., Liu, R., Borad, N., Ziyaei, S., Ghobadi, M., Sabbah, F., and Aker, A. (2018). Multi-lingual argumentative corpora in English, Turkish, Greek, Albanian, Croatian, Serbian, Macedonian, Bulgarian, Romanian and Arabic. In Proceedings of the Eleventh International Conference on Language Resources and Evaluation (LREC 2018) (European Language Resources Association (ELRA)).

253. Sardianos, C., Katakis, I.M., Petasis, G., and Karkaletsis, V. (2015). Argument extraction from news. In Proceedings of the 2nd Workshop on Argumentation Mining, pp. 56–66.

254. Lafferty, J.D., McCallum, A., and Pereira, F.C. (2001). Conditional random fields: Probabilistic models for segmenting and labeling sequence data. In Proceedings of the Eighteenth International Conference on Machine Learning, pp. 282–289.

255. Chen, Y., and Skiena, S. (2014). Building sentiment lexicons for all major languages. In Proceedings of the 52nd Annual Meeting of the Association for Computational Linguistics (Volume 2: Short Papers), pp. 383–389. https://doi.org/10.3115/v1/P14-2063.

256. Patsiouras, E., Koroni, I., Mademlis, I., and Pitas, I. (2023). Auth Greek politics dataset. In Artificial Intelligence and Information Analysis Group, Aristotle University of Thessaloniki https://aiia.csd.auth.gr/auth-greekpolitics-dataset.

257. Bilianos, D. (2020). Greek sentiment analysis. GitHub. https://github.com/DimitrisBil/greek-sentiment-analysis.

258. Chatzakou, D., Vakali, A., and Kafetsios, K. (2016). Annotated twitter dataset for emotion detection. Dropbox. http://bit.ly/2bLgVUP.

259. Antonakaki, D., Spiliotopoulos, D., V. Samaras, C., Pratikakis, P., Ioannidis, S., and Fragopoulou, P. (2017). Social media analysis during political turbulence. Figshare. https://figshare.com/articles/dataset/Social_media_analysis_during_political_turbulence_DATA/5492443/1.

260. Makrynioti, N., and Vassalos, V. (2015). Sentiment extraction from tweets: Multilingual challenges. In Big Data Analytics and Knowledge Discovery, S. Madria and T. Hara, eds. (Springer International Publishing), pp. 136–148. https://doi.org/10.1007/978-3-319-22729-0_11.

261. Charalampakis, B., Spathis, D., Kouslis, E., and Kermanidis, K. (2015). Websent: A web-based sentiment analysis tool. In Humanistic Informatics Laboratory, Ionian University https://di.ionio.gr/hilab/doku.php.

262. Tsakalidis, A., Papadopoulos, S., and Kompatsiaris, Y. (2017). Building and evaluating resources for sentiment analysis in the Greek language. In Multimedia Knowledge and Social Media Analytics Laboratory, CERTH http://mklab.iti.gr/resources/tsakalidis2017building.zip.

263. mSensis. Demon: Deep emotion understanding. https://msensis.com/research-and-development/demon.

264. Palogiannidi, E., Koutsakis, P., Iosif, E., and Potamianos, A. (2016). Greek Affective Lexicon Automatically Created (Technical University of Crete). https://www.telecom.tuc.gr/~epalogiannidi/docs/resources/greek_affective_lexicon_automatically_created.zip.

265. Apache Software Foundation (2004). Apache license, version 2.0. https://www.apache.org/licenses/LICENSE-2.0.html.

266. X, Inc. (2024). X terms of service. https://x.com/en/tos.

267. Avgi SA. Avgi: the morning newspaper of the left. https://www.avgi.gr/.

268. Manolis Triantafyllidis Foundation. Dictionary of standard modern Greek. Institute for Modern Greek Studies, Aristotle University of Thessaloniki. https://www.greek-language.gr/greekLang/modern_greek/tools/lexica/triantafyllides/.

269. Tsakalidis, A., Papadopoulos, S., and Kompatsiaris, I. (2014). An ensemble model for cross-domain polarity classification on twitter. In Web Information Systems Engineering–WISE 2014: 15th International Conference, Thessaloniki, Greece, October 12-14, 2014, Proceedings, Part II 15 (Springer), pp. 168–177. https://doi.org/10.1007/978-3-319-11746-1_12.

270. Bradley, M.M., and Lang, P.J. (1999). Affective norms for English words (anew): Instruction manual and affective ratings. In Tech. Rep. Technical report C-1, the center for research in psychophysiology.

271. Wankhade, M., Rao, A.C.S., and Kulkarni, C. (2022). A survey on sentiment analysis methods, applications, and challenges. Artif. Intell. Rev. *55*, 5731–5780. https://doi.org/10.1007/s10462-022-10144-1.

272. El, S.E.M., and Kassou, I. (2014). Authorship analysis studies: A survey. Int. J. Comput. Appl. *86*, 22–29. https://doi.org/10.5120/15038-3384.

273. Kestemont, M., Tschuggnall, M., Stamatatos, E., Daelemans, W., Specht, G., Stein, B., and Potthast, M. (2018). Overview of the author identification task at pan-2018: cross-domain authorship attribution and style change detection. In Working Notes Papers of the CLEF 2018 Evaluation Labs. Avignon, France, September 10-14, 2018/Cappellato, Linda [edit.]; et al., pp. 1–25.

274. Rosso, P., Rangel, F., Potthast, M., Stamatatos, E., Tschuggnall, M., and Stein, B. (2016). Overview of pan’16. In Experimental IR Meets Multilinguality, Multimodality, and Interaction, N. Fuhr, P. Quaresma, T. Gonçalves, B. Larsen, K. Balog, C. Macdonald, L. Cappellato, and N. Ferro, eds. (Springer International Publishing), pp. 332–350. https://doi.org/10.1007/978-3-319-44564-9_28.

275. Webis Group. Pan: Digital text forensics and stylometry. https://pan.webis.de/.

276. Bevendorff, J., Chinea-Ríos, M., Franco-Salvador, M., Heini, A., Körner, E., Kredens, K., Mayerl, M., Pęzik, P., Potthast, M., Rangel, F., et al. (2023). Overview of pan 2023: Authorship verification, multi-author writing style analysis, profiling cryptocurrency influencers, and trigger detection. In European Conference on Information Retrieval (Springer), pp. 518–526.

277. Juola, P., and Stamatatos, E. (2013). Overview of the author identification task at pan 2013. In Working Notes for CLEF 2013 Conference, *1179*, P. Forner, R. Navigli, D. Tufis, and N. Ferro, eds. *(CEUR-WS.org), pp. 1–20.* https://ceur-ws.org/Vol-1179/CLEF2013wn-PAN-JuolaEt2013.pdf.

278. Stamatatos, E., Daelemans, W., Verhoeven, B., Potthast, M., Stein, B., Juola, P., Sanchez-Perez, M.A., and Barrón-Cedeño, A. (2014). Overview of the author identification task at pan 2014. In CEUR Workshop Proceedings, *1180* (CEUR-WS), pp. 877–897.

279. Stamatatos, E., Daelemans, W., Verhoeven, B., Juola, P., López López, A., Potthast, M., and Stein, B. (2015). Overview of the Author Identification Task at PAN 2015. In Working Notes Papers of the CLEF 2015 Evaluation Labs vol. 1391 of *Lecture Notes in Computer Science*, L. Cappellato, N. Ferro, G. Jones, and E. San Juan, eds., pp. 877–897.

280. Stamatatos, E., Tschuggnall, M., Verhoeven, B., Daelemans, W., Specht, G., Stein, B., and Potthast, M. (2016). Clustering by Authorship Within and Across Documents. In Working Notes Papers of the CLEF 2016 Evaluation Labs vol. 1609 of *Lecture Notes in Computer Science*, K. Balog, L. Cappellato, N. Ferro, and C. Macdonald, eds., pp. 691–715.

281. Juola, P., Mikros, G.K., and Vinsick, S. (2019). A comparative assessment of the difficulty of authorship attribution in Greek and in English. J. Assoc. Inf. Sci. Technol. *70*, 61–70. https://doi.org/10.1002/asi.24073.

282. Juola, P. (2020). Jgaap: Java graphical authorship attribution program. GitHub. https://github.com/evllabs/JGAAP.

283. Kocher, M., and Savoy, J. (2017). A simple and efficient algorithm for authorship verification. J. Assoc. Inf. Sci. Technol. *68*, 259–269. https://doi.org/10.1002/asi.23648.

284. Hürlimann, M., Weck, B., van den Berg, E., Suster, S., and Nissim, M. (2015). Glad: Groningen lightweight authorship detection. In Proceedings of CLEF 2015 Labs and Workshops, Notebook Papers, CEUR Workshop, pp. 1–12.

285. Hürlimann, M., Weck, B., van den Berg, E., Suster, S., and Nissim, M. (2015). Glad: Groningen lightweight authorship detection. GitHub. URL: https://github.com/pan-webis-de/huerlimann15.

CellPress
OPEN ACCESS

286. Halvani, O., Winter, C., and Pflug, A. (2016). Authorship verification for different languages, genres and topics. Digit. Invest. *16*, S33–S43. https://doi.org/10.1016/j.diin.2016.01.006.

287. Halvani, O., Graner, L., and Regev, R. (2020). Taveer: an interpretable topic-agnostic authorship verification method. In Proceedings of the 15th International Conference on Availability, Reliability and Security, pp. 1–10.

288. Kestemont, M., Manjavacas, E., Markov, I., Bevendorff, J., Wiegmann, M., Stamatatos, E., Potthast, M., and Stein, B. (2020). Overview of the cross-domain authorship verification task at pan 2020. In Working notes of CLEF 2020-Conference and Labs of the Evaluation Forum, 22-25 September, Thessaloniki, Greece, pp. 1–14.

289. Mikros, G.K. (2013). Systematic stylometric differences in men and women authors: A corpus-based study. In Issues in Quantitative Linguistics 3 vol. 13 of *Studies in Quantitative Linguistics*, R. Köhler and G. Altmann, eds. (RAM-Verlag), pp. 206–223. https://www.isbn.de/buch/9783942303125/issues-in-quantitative-linguistics-3.

290. Mikros, G.K., and Perifanos, K. (2015). Gender identification in modern Greek tweets. Recent Contributions to Quantitative Linguistics *70*, 75–88. https://doi.org/10.1515/9783110420296-008.

291. Mikros, G.K. (2012). Authorship attribution and gender identification in Greek blogs. Methods and Applications of Quantitative Linguistics *21*, 21–32.

292. Platt, J.C. (1999). Fast training of support vector machines using sequential minimal optimization. In Advances in Kernel Methods: Support Vector Learning, B. Schölkopf, C.J. Burges, and A.J. Smola, eds. (MIT Press), pp. 185–208. https://doi.org/10.7551/mitpress/1130.003.0016.

293. Alter Ego Media S.A. To vima: Greek news portal. https://www.tovima.gr/.

294. Stamatatos, E., Tschuggnall, M., Verhoeven, B., Daelemans, W., Specht, G., Stein, B., and Potthast, M. (2016). Pan16 author identification: Clustering. Zenodo. https://doi.org/10.5281/zenodo.3737586.

295. Stamatatos, E., Daelemans, W., Verhoeven, B., Juola, P., López-López, A., Potthast, M., and Stein, B. (2015). Pan15 author identification: Verification. Zenodo. https://doi.org/10.5281/zenodo.3737562.

296. Stamatatos, E., Daelemans, W., Verhoeven, B., Potthast, M., Stein, B., Juola, P., Sanchez-Perez, M.A., and Barrón-Cedeño, A. (2014). Pan14 author identification: Verification. Zenodo. URL: https://doi.org/10.5281/zenodo.3716033. doi: https://doi.org/10.5281/zenodo.3716033.

297. Juola, P., and Stamatatos, E. (2013). Pan13 author identification: Verification. Zenodo. URL: https://doi.org/10.5281/zenodo.3715999. doi: https://doi.org/10.5281/zenodo.3715999.

298. Halvani, O., Winter, C., and Pflug, A. (2015). Halvani, winter and plug dataset. http://bit.ly/1OjFRhJ.

299. Savoy, J. (2020). Machine Learning Methods for Stylometry: Authorship Attribution and Author Profiling (Springer). https://doi.org/10.1007/978-3-030-53360-1. https://link.springer.com/book/10.1007/978-3-030-53360-1.

300. Potthast, M., Rosso, P., Stamatatos, E., and Stein, B. (2019). A decade of shared tasks in digital text forensics at pan. In Advances in Information Retrieval: 41st European Conference on IR Research, ECIR 2019, Cologne, Germany, April 14–18, 2019, Proceedings, Part II 41 (Springer), pp. 291–300.

301. D. Hovy, S. Spruit, M. Mitchell, E.M. Bender, M. Strube, and H. Wallach, eds. (2017). Proceedings of the First ACL Workshop on Ethics in Natural Language Processing (Association for Computational Linguistics). https://doi.org/10.18653/v1/W17-16.

302. Schmidt, A., and Wiegand, M. (2017). A survey on hate speech detection using natural language processing. In Proceedings of the Fifth International Workshop on Natural Language Processing for Social Media, pp. 1–10.

303. Díaz, M., Amironesei, R., Weidinger, L., and Gabriel, I. (2022). Accounting for offensive speech as a practice of resistance. In Proceedings of the Sixth Workshop on Online Abuse and Harms (WOAH), pp. 192–202.

304. Welbl, J., Glaese, A., Uesato, J., Dathathri, S., Mellor, J., Hendricks, L.A., Anderson, K., Kohli, P., Coppin, B., and Huang, P.S. (2021). Challenges in detoxifying language models. Preprint at arXiv. https://doi.org/10.48550/arXiv.2109.07445.

305. Pavlopoulos, J., Sorensen, J., Dixon, L., Thain, N., and Androutsopoulos, I. (2020). Toxicity detection: Does context really matter?. Preprint at arXiv. https://doi.org/10.48550/arXiv.2006.00998.

306. Hovy, D., and Yang, D. (2021). The importance of modeling social factors of language: Theory and practice. In Proceedings of the 2021 Conference of the North American Chapter of the Association for Computational Linguistics: Human Language Technologies, pp. 588–602.

307. Sap, M., Card, D., Gabriel, S., Choi, Y., and Smith, N.A. (2019). The risk of racial bias in hate speech detection. In Proceedings of the 57th annual meeting of the association for computational linguistics, pp. 1668–1678.

308. Gordon, M.L., Lam, M.S., Park, J.S., Patel, K., Hancock, J., Hashimoto, T., and Bernstein, M.S. (2022). Jury learning: Integrating dissenting voices into machine learning models. In Proceedings of the 2022 CHI Conference on Human Factors in Computing Systems, pp. 1–19. https://doi.org/10.1145/3491102.350200.

309. Pavlopoulos, J., and Likas, A. (2024). Polarized opinion detection improves the detection of toxic language. In Proceedings of the 18th Conference of the European Chapter of the Association for Computational Linguistics (Volume 1: Long Papers), pp. 1946–1958.

310. Zampieri, M., Nakov, P., Rosenthal, S., Atanasova, P., Karadzhov, G., Mubarak, H., Derczynski, L., Pitenis, Z., and Çöltekin, Ç. (2020). Semeval-2020 task 12: Multilingual offensive language identification in social media (offenseval 2020). Preprint at arXiv. https://doi.org/10.48550/arXiv.2006.07235.

311. Wang, S., Liu, J., Ouyang, X., and Sun, Y. (2020). Galileo at semeval-2020 task 12: Multi-lingual learning for offensive language identification using pre-trained language models. Preprint at arXiv. https://doi.org/10.48550/arXiv.2010.03542.

312. Socha, K. (2020). Ks@ lth at semeval-2020 task 12: Fine-tuning multi-and monolingual transformer models for offensive language detection. In Proceedings of the Fourteenth Workshop on Semantic Evaluation, pp. 2045–2053.

313. Plum, A., Ranasinghe, T., Orasan, C., and Mitkov, R. (2019). Rgcl at Germeval 2019: Offensive Language Detection with Deep Learning (German Society for Computational Linguistics & Language Technology).

314. Zampieri, M., Malmasi, S., Nakov, P., Rosenthal, S., Farra, N., and Kumar, R. (2019). Semeval-2019 task 6: Identifying and categorizing offensive language in social media (offenseval). In Proceedings of the 13th International Workshop on Semantic Evaluation, pp. 75–86.

315. Zampieri, M., Malmasi, S., Nakov, P., Rosenthal, S., Farra, N., and Kumar, R. (2019). Predicting the type and target of offensive posts in social media. Preprint at arXiv. https://doi.org/10.48550/arXiv.1902.09666.

316. Liquid Media. Gazzetta. http://www.gazzetta.gr/.

317. Arvanitidis, P., Papagiannitsis, G., Desli, A.Z., Vergou, P., and Gourgouliani, S. (2021). Attitudes towards refugees & immigrants in greece: A national-local comparative analysis. European Journal of Geography *12*, 39–55. https://doi.org/10.48088/ejg.p.arv.12.3.39.55.

318. Pontiki, M., Gavriilidou, M., Gkoumas, D., and Piperidis, S. (2020). Verbal aggression as an indicator of xenophobic attitudes in Greek Twitter during and after the financial crisis. In Proceedings of the Workshop about Language Resources for the SSH Cloud (European Language Resources Association), pp. 19–26.

319. Kotsakis, R., Vrysis, L., Vryzas, N., Saridou, T., Matsiola, M., Veglis, A., and Dimoulas, C. (2023). A web framework for information aggregation and management of multilingual hate speech. Heliyon *9*, e16084. https://doi.org/10.1016/j.heliyon.2023.e16084.

320. The PHARM Project Team (2021). Pharm scripts. GitHub. https://github.com/thepharmproject/set_of_scripts.

321. Lekea, I.K., and Karampelas, P. (2018). Detecting hate speech within the terrorist argument: a Greek case. In 2018 IEEE/ACM International

Conference on Advances in Social Networks Analysis and Mining (ASONAM) (IEEE), pp. 1084–1091. https://doi.org/10.1109/ASONAM.2018.8508270.

322. Nikiforos, S., Tzanavaris, S., and Kermanidis, K.L. (2020). Virtual learning communities (vlcs) rethinking: influence on behavior modification—bullying detection through machine learning and natural language processing. J. Comput. Educ. *7*, 531–551. https://doi.org/10.1007/s40692-020-00166-5.

323. Strømberg, A. (2020). Offenseval 2020 dataset. Hugging Face. https://huggingface.co/datasets/strombergnlp/offenseval_2020.

324. Perifanos, K., and Goutsos, D. (2021). Multimodal hate speech detection. GitHub. https://github.com/kperi/MultimodalHateSpeechDetection.

325. Pontiki, M. Clarin: Xenophobia resources. CLARIN:EL. https://inventory.clarin.gr/search/xenophobia.

326. Pavlopoulos, J., Malakasiotis, P., and Androutsopoulos, I. (2015). Gazzetta comments dataset. https://archive.org/details/gazzetta-comments-dataset.tar.

327. PHARM Project Consortium. Pharm Project. University of Salamanca. https://pharmproject.usal.es/.

328. M. Alfano, D. Hovy, M. Mitchell, and M. Strube, eds. (2018). Proceedings of the Second ACL Workshop on Ethics in Natural Language Processing (Association for Computational Linguistics). https://doi.org/10.18653/v1/W18-08.

329. El-Kassas, W.S., Salama, C.R., Rafea, A.A., and Mohamed, H.K. (2021). Automatic text summarization: A comprehensive survey. Expert Syst. Appl. *165*, 113679. https://doi.org/10.1016/j.eswa.2020.113679.

330. Maybury, M.T. (1995). Generating summaries from event data. Inf. Process. Manag. *31*, 735–751. https://doi.org/10.1016/0306-4573(95)00025-C.

331. Luhn, H.P. (1958). The automatic creation of literature abstracts. IBM J. Res. Dev. *2*, 159–165. https://doi.org/10.1147/rd.22.0159.

332. Giarelis, N., Mastrokostas, C., and Karacapilidis, N. (2023). Abstractive vs. extractive summarization: An experimental review. Appl. Sci. *13*, 7620. https://doi.org/10.3390/app13137620.

333. Alomari, A., Idris, N., Sabri, A.Q.M., and Alsmadi, I. (2022). Deep reinforcement and transfer learning for abstractive text summarization: A review. Comput. Speech Lang *71*, 101276.

334. El-Haj, M., Zmandar, N., Rayson, P., AbuRa'ed, A., Litvak, M., Pittaras, N., Giannakopoulos, G., Kosmopoulos, A., Carbajo-Coronado, B., and Moreno-Sandoval, A. (2022). The financial narrative summarisation shared task (FNS 2022). In Proceedings of the 4th Financial Narrative Processing Workshop @LREC2022 (European Language Resources Association), pp. 43–52.

335. Zavitsanos, E., Kosmopoulos, A., Giannakopoulos, G., Litvak, M., Carbajo-Coronado, B., Moreno-Sandoval, A., and El-Haj, M. (2023). The financial narrative summarisation shared task (fns 2023). In 2023 IEEE International Conference on Big Data (BigData) (IEEE), pp. 2890–2896. https://doi.org/10.1109/BigData59044.2023.10386228.

336. Shukla, N.K., Katikeri, R., Raja, M., Sivam, G., Yadav, S., Vaid, A., and Prabhakararao, S. (2023). Generative ai approach to distributed summarization of financial narratives. In 2023 IEEE International Conference on Big Data (BigData) (IEEE), pp. 2872–2876. https://doi.org/10.1109/BigData59044.2023.10386313.

337. Vanetik, N., Podkaminer, E., and Litvak, M. (2023). Summarizing financial reports with positional language model. In 2023 IEEE International Conference on Big Data (BigData) (IEEE), pp. 2877–2883. https://doi.org/10.1109/BigData59044.2023.10386704.

338. Shukla, N., Vaid, A., Katikeri, R., Keeriyadath, S., and Raja, M. (2022). Dimsum: Distributed and multilingual summarization of financial narratives. In Proceedings of the 4th Financial Narrative Processing Workshop@ LREC2022, pp. 65–72.

339. Liu, Y. (2019). Fine-tune bert for extractive summarization. Preprint at arXiv. https://doi.org/10.48550/arXiv.1903.10318.

340. Lv, Y., and Zhai, C. (2009). Positional language models for information retrieval. In Proceedings of the 32nd International ACM SIGIR Conference on Research and Development in Information Retrieval, pp. 299–306. https://doi.org/10.1145/1571941.1571994.

341. Giannakopoulos, G. (2013). Multi-document multilingual summarization and evaluation tracks in ACL 2013 MultiLing Workshop. In Proceedings of the MultiLing 2013 Workshop on Multilingual Multi-Document Summarization, pp. 20–28.

342. Giarelis, N., Mastrokostas, C., and Karacapilidis, N. (2024). Greekt5: A series of Greek sequence-to-sequence models for news summarization. GitHub. https://github.com/NC0DER/GreekT5.

343. Koniaris, M., Galanis, D., Giannini, E., and Tsanakas, P. (2023). Evaluation of automatic legal text summarization techniques for Greek case law. Information *14*, 250. https://doi.org/10.3390/info14040250.

344. Erkan, G., and Radev, D.R. (2004). Lexrank: Graph-based lexical centrality as salience in text summarization. J. Artif. Intell. Res. *22*, 457–479. https://doi.org/10.1613/jair.1523.

345. Otterbacher, J., Erkan, G., and Radev, D.R. (2009). Biased lexrank: Passage retrieval using random walks with question-based priors. Inf. Process. Manag. *45*, 42–54. https://doi.org/10.1016/j.ipm.2008.06.004.

346. DominusTea (2023). Greeklegalsum: A Greek legal summarization dataset. Hugging Face. https://huggingface.co/datasets/DominusTea/GreekLegalSum.

347. Evdaimon, I. (2022). Greeksum: A Greek news summarization dataset. GitHub. https://github.com/iakovosevdaimon/GreekSUM.

348. Zavitsanos, E., Kosmopoulos, A., Giannakopoulos, G., Litvak, M., Carbajo-Coronado, B., Moreno-Sandoval, A., and El-Haj, M. (2023). The Financial Narrative Summarisation Shared Task (FNS 2023) (IEEE Computer Society Press). https://doi.org/10.1109/BigData59044.2023.10386228.

349. European Language Resources Association (2022). 4th Financial Narrative Processing Workshop (FNP 2022) (European Language Resources Association). https://aclanthology.org/2022.fnp-1.0.pdf.

350. Li, L., Forăscu, C., El-Haj, M., and Giannakopoulos, G. (2013). Multi-document multilingual summarization corpus preparation, part 1: Arabic, English, Greek, Chinese, Romanian. In Proceedings of the MultiLing 2013 Workshop on Multilingual Multi-Document Summarization, pp. 1–12.

351. Li, L., Forăscu, C., El-Haj, M., and Giannakopoulos, G. (2014). MultiLing community datasets. NCSR Demokritos. http://multiling.iit.demokritos.gr/pages/view/1571/datasets.

352. Areios Pagos. Areios pagos: Supreme civil and criminal court of greece. https://www.areiospagos.gr/.

353. Wikimedia Foundation. Wikinews: Free news source. https://www.wikinews.org/.

354. Zhu, F., Lei, W., Wang, C., Zheng, J., Poria, S., and Chua, T. (2021). Retrieving and reading: A comprehensive survey on open-domain question answering. Preprint at arXiv. https://doi.org/10.48550/arXiv.2101.00774.

355. Goodwin, T.R., and Harabagiu, S.M. (2016). Medical question answering for clinical decision support. In Proceedings of the 25th ACM international on conference on information and knowledge management, pp. 297–306.

356. Li, Y., Miao, Q., Geng, J., Alt, C., Schwarzenberg, R., Hennig, L., Hu, C., and Xu, F. (2018). Question answering for technical customer support. In Natural Language Processing and Chinese Computing: 7th CCF International Conference, NLPCC 2018, Hohhot, China, August 26–30, 2018, Proceedings, Part I 7 (Springer), pp. 3–15.

357. Adamopoulou, E., and Moussiades, L. (2020). Chatbots: History, technology, and applications. Machine Learning with Applications *2*, 100006. https://doi.org/10.1016/j.mlwa.2020.100006.

358. de Barcelos Silva, A., Gomes, M.M., da Costa, C.A., da Rosa Righi, R., Barbosa, J.L.V., Pessin, G., De Doncker, G., and Federizzi, G. (2020).

CellPress
OPEN ACCESS

Intelligent personal assistants: A systematic literature review. Expert Syst. Appl. *147*, 113193. https://doi.org/10.1016/j.eswa.2020.113193.

359. Gardner, M., Berant, J., Hajishirzi, H., Talmor, A., and Min, S. (2019). Question answering is a format; when is it useful?. Preprint at arXiv. https://doi.org/10.48550/arXiv.1909.11291.

360. OpenAI. Chatgpt by openai. https://chat.openai.com.

361. Chen, D., and Yih, W.t. (2020). Open-domain question answering. In Proceedings of the 58th Annual Meeting of the Association for Computational Linguistics: Tutorial Abstracts, A. Savary and Y. Zhang, eds. (Association for Computational Linguistics), pp. 34–37. https://doi.org/10.18653/v1/2020.acl-tutorials.8.

362. Rogers, A., Gardner, M., and Augenstein, I. (2023). Qa dataset explosion: A taxonomy of NLP resources for question answering and reading comprehension. ACM Comput. Surv. *55*, 1–45. https://doi.org/10.1145/3560260.

363. Schlegel, V., Valentino, M., Freitas, A., Nenadic, G., and Batista-Navarro, R. (2020). A framework for evaluation of machine reading comprehension gold standards. Preprint at arXiv. https://doi.org/10.48550/arXiv.2003.04642.

364. Sugawara, S., Kido, Y., Yokono, H., and Aizawa, A. (2017). Evaluation metrics for machine reading comprehension: Prerequisite skills and readability. In Proceedings of the 55th Annual Meeting of the Association for Computational Linguistics (Volume 1: Long Papers), pp. 806–817.

365. DBpedia Association. Dbpedia: Structured information from wikipedia. https://www.dbpedia.org/.

366. Marakakis, E., Kondylakis, H., and Aris, P. (2017). Apantisis: A Greek question-answering system for knowledge-base exploriaton. In Strategic Innovative Marketing, A. Kavoura, D.P. Sakas, and P. Tomaras, eds. (Springer International Publishing), pp. 501–510. https://doi.org/10.1007/978-3-319-56288-9_67.

367. Braun, D., Mendez, A.H., Matthes, F., and Langen, M. (2017). Evaluating natural language understanding services for conversational question answering systems. In Proceedings of the 18th annual SIGdial meeting on discourse and dialogue, pp. 174–185.

368. Malamas, N., Papangelou, K., and Symeonidis, A.L. (2022). Upon improving the performance of localized healthcare virtual assistants. Healthcare *10*, 99. https://doi.org/10.3390/healthcare10010099.

369. Ventoura, N., Palios, K., Vasilakis, Y., Paraskevopoulos, G., Katsamanis, N., and Katsouros, V. (2021). Theano: A Greek-speaking conversational agent for COVID-19. In Proceedings of the 1st Workshop on NLP for Positive Impact (Association for Computational Linguistics), pp. 36–46. https://doi.org/10.18653/v1/2021.nlp4posimpact-1.5.

370. Rasa Technologies GmbH. Rasa: Open source conversational ai. https://rasa.com/.

371. Centre for the Greek Language. Text bank. https://www.greek-language.gr/certification/dbs/teachers/.

372. Lopes, J., Chorianopoulou, A., Palogiannidi, E., Moniz, H., Abad, A., Louka, K., Iosif, E., and Potamianos, A. (2016). The SpeDial datasets: datasets for spoken dialogue systems analytics. In Proceedings of the Tenth International Conference on Language Resources and Evaluation (LREC'16) (European Language Resources Association (ELRA)), pp. 104–110.

373. Bastakis, M. (2023). Tiresias evaluation results. GitHub. https://github.com/mbastakis/Tiresias/tree/master/evaluation_results.

374. Hutchins, J. (1997). From first conception to first demonstration: the nascent years of machine translation, 1947–1954. a chronology. Mach. Transl. *12*, 195–252. https://doi.org/10.1023/A:1007969630568.

375. Kouremenos, D., Ntalianis, K., and Kollias, S. (2018). A novel rule based machine translation scheme from Greek to Greek sign language: Production of different types of large corpora and language models evaluation. Comput. Speech Lang *51*, 110–135. https://doi.org/10.1016/j.csl.2018.04.001.

376. Beinborn, L., Zesch, T., and Gurevych, I. (2013). Cognate production using character-based machine translation. In Proceedings of the sixth international joint conference on natural language processing, pp. 883–891.

377. Crystal, D. (2011). A Dictionary of Linguistics and Phonetics (John Wiley & Sons).

378. Koehn, P., Hoang, H., Birch, A., Callison-Burch, C., Federico, M., Bertoldi, N., Cowan, B., Shen, W., Moran, C., Zens, R., et al. (2007). Moses: Open source toolkit for statistical machine translation. In Proceedings of the 45th annual meeting of the association for computational linguistics companion volume proceedings of the demo and poster sessions, pp. 177–180.

379. Pecina, P., Toral, A., Papavassiliou, V., Prokopidis, P., and Van Genabith, J. (2012). Domain adaptation of statistical machine translation using web-crawled resources: a case study. In Proceedings of the 16th Annual Conference of the European Association for Machine Translation, pp. 145–152.

380. Mouratidis, D., Kermanidis, K.L., and Sosoni, V. (2021). Innovatively fused deep learning with limited noisy data for evaluating translations from poor into rich morphology. Appl. Sci. *11*, 639. https://doi.org/10.3390/app11020639.

381. Stasimioti, M., Sosoni, V., Kermanidis, K., and Mouratidis, D. (2020). Machine translation quality: A comparative evaluation of SMT, NMT and tailored-NMT outputs. In Proceedings of the 22nd Annual Conference of the European Association for Machine Translation. Lisboa, Portugal: European Association for Machine Translation, pp. 441–450.

382. Mouratidis, D., Kermanidis, K.L., and Sosoni, V. (2020). Innovative deep neural network fusion for pairwise translation evaluation. In Artificial Intelligence Applications and Innovations: 16th IFIP WG 12.5 International Conference, AIAI 2020, Neos Marmaras, Greece, June 5–7, 2020, Proceedings, Part II 16 (Springer), pp. 76–87. https://doi.org/10.1007/978-3-030-49186-4_7.

383. Mouratidis, D., and Kermanidis, K.L. (2019). Ensemble and deep learning for language-independent automatic selection of parallel data. Algorithms *12*, 26. https://doi.org/10.3390/a12010026.

384. Castilho, S., Moorkens, J., Gaspari, F., Sennrich, R., Way, A., and Georgakopoulou, P. (2018). Evaluating mt for massive open online courses: A multifaceted comparison between pbsmt and nmt systems. Mach. Translat. *32*, 255–278. https://doi.org/10.1007/s10590-018-9221-y.

385. Giorgi, I., Golosio, B., Esposito, M., Cangelosi, A., and Masala, G.L. (2021). Modeling multiple language learning in a developmental cognitive architecture. IEEE Trans. Cogn. Dev. Syst. *13*, 922–933. https://doi.org/10.1109/TCDS.2020.3033963.

386. Gamallo, P., Pichel, J.R., and Alegria, I. (2020). Measuring language distance of isolated european languages. Information *11*, 181. https://doi.org/10.3390/info11040181.

387. Fragkou, P. (2014). Text segmentation for language identification in Greek forums. Proced. Soc. Behav. Sci. *147*, 160–166. https://doi.org/10.1016/j.sbspro.2014.07.140.

388. Bollegala, D., Kontonatsios, G., and Ananiadou, S. (2015). A cross-lingual similarity measure for detecting biomedical term translations. PLoS One *10*, e0126196. https://doi.org/10.1371/journal.pone.0126196.

389. Pfeiffer, J., Vulić, I., Gurevych, I., and Ruder, S. (2020). Mad-x: An adapter-based framework for multi-task cross-lingual transfer. Preprint at arXiv. https://doi.org/10.48550/arXiv.2005.00052.

390. Papaioannou, J.M., Grundmann, P., van Aken, B., Samaras, A., Kyparissidis, I., Giannakoulas, G., Gers, F., and Loeser, A. (2022). Cross-lingual knowledge transfer for clinical phenotyping. GitHub. https://github.com/neuron1682/cross-lingual-phenotype-prediction/tree/main.

391. Singh, J., McCann, B., Keskar, N.S., Xiong, C., and Socher, R. (2019). Xlda: Cross-lingual data augmentation for natural language inference

and question answering. Preprint at arXiv. https://doi.org/10.48550/arxiv.1905.11471.

392. Papadopoulos, D., Papadakis, N., and Matsatsinis, N.F. (2021). lighteternal models. Hugging Face. https://huggingface.co/lighteternal.

393. Prokopidis, P., Papavassiliou, V., and Piperidis, S. (2016). Parallel Global Voices: a collection of multilingual corpora with citizen media stories. In Proceedings of the Tenth International Conference on Language Resources and Evaluation (LREC'16) (European Language Resources Association (ELRA)), pp. 900–905.

394. Global Voices. Global voices. https://globalvoices.org/.

395. Prokopidis, P., Papavassiliou, V., and Piperidis, S. Pgv datasets). http://nlp.ilsp.gr/pgv/.

396. Bollegala, D., Kontonatsios, G., and Ananiadou, S. (2015). S1 dataset. PLoS One. https://doi.org/10.1371/journal.pone.0126196.

397. Hugging Face, Inc. Hugging face. https://huggingface.co.

398. CLARIN:EL. Clarin:el research infrastructure for language resources & technologies. CLARIN:EL. https://inventory.clarin.gr/.

399. Hugging Face. Hugging face datasets: Greek translation. https://huggingface.co/datasets?task_categories=task_categories:translation&language=language:el&sort=downloads.

400. Hugging Face. Hugging face models: Greek translation. https://huggingface.co/models?pipeline_tag=translation&language=el&sort=downloads.

401. Hugging Face. Hugging face datasets. https://huggingface.co/datasets.

402. Dabre, R., Chu, C., and Kunchukuttan, A. (2020). A survey of multilingual neural machine translation. ACM Comput. Surv. *53*, 1–38. https://doi.org/10.1145/3406095.

403. Haddow, B., Bawden, R., Barone, A.V.M., Helcl, J., and Birch, A. (2022). Survey of low-resource machine translation. Comput. Linguist. *48*, 673–732. https://doi.org/10.1162/coli_a_00446.

404. Papaloukas, C., Chalkidis, I., Athinaios, K., Pantazi, D., and Koubarakis, M. (2021). Multi-granular legal topic classification on Greek legislation. In Proceedings of the Natural Legal Language Processing Workshop 2021 (Association for Computational Linguistics), pp. 63–75.

405. Lachana, Z., Loutsaris, M.A., Alexopoulos, C., and Charalabidis, Y. (2020). Automated analysis and interrelation of legal elements based on text mining. Int. J. E Serv. Mobile Appl. *12*, 79–96. https://doi.org/10.4018/IJESMA.2020040105.

406. Garofalakis, J., Plessas, K., and Plessas, A. (2016). A semi-automatic system for the consolidation of Greek legislative texts. In Proceedings of the 20th Pan-Hellenic Conference on Informatics. PCI '16 (Association for Computing Machinery), pp. 1–6.

407. Paraskevopoulos, G., Pistofidis, P., Banoutsos, G., Georgiou, E., and Katsouros, V. (2022). Multimodal classification of safety-report observations. Appl. Sci. *12*, 5781. https://doi.org/10.3390/app12125781.

408. Boskou, G., Kirkos, E., and Spathis, C. (2018). Assessing internal audit with text mining. J. Info. Know. Mgmt. *17*, 1850020. https://doi.org/10.1142/S021964921850020X.

409. Chatzipanagiotidis, S., Giagkou, M., and Meurers, D. (2021). Broad linguistic complexity analysis for Greek readability classification. In Proceedings of the 16th Workshop on Innovative Use of NLP for Building Educational Applications, pp. 48–58.

410. Piskorski, J., Stefanovitch, N., Da San Martino, G., and Nakov, P. (2023). Semeval-2023 task 3: Detecting the category, the framing, and the persuasion techniques in online news in a multi-lingual setup. In Proceedings of the 17th International Workshop on Semantic Evaluation (SemEval-2023), pp. 2343–2361.

411. Athanasiou, M., Fragkozidis, G., Zarkogianni, K., and Nikita, K.S. (2023). Long short-term memory–based prediction of the spread of influenza-like illness leveraging surveillance, weather, and twitter data: Model development and validation. J. Med. Internet Res. *25*, e42519. https://doi.org/10.2196/42519.

412. Stamouli, S., Nerantzini, M., Papakyritsis, I., Katsamanis, A., Chatzoudis, G., Dimou, A.L., Plitsis, M., Katsouros, V., Varlokosta, S., and Terzi, A. (2023). A web-based application for eliciting narrative discourse from Greek-speaking people with and without language impairments. Front. Commun. *8*, 919617. https://doi.org/10.3389/fcomm.2023.919617.

413. Hellenic Republic. Greek government gazette. https://raptarchis.gov.gr.

414. Papaloukas, C., Chalkidis, I., Athinaios, K., Pantazi, D., and Koubarakis, M. (2021). Greek legal code dataset. Hugging Face. https://huggingface.co/datasets/greek_legal_code.

415. Garofalakis, J., Plessas, K., and Plessas, A. (2016). Greek laws alpha. GitHub. https://github.com/OpenLawsGR/greek_laws_alpha.

416. Liu, Z., Sun, C., Jiang, Y., Jiang, S., and Ming, M. (2021). Multi-modal application: Image memes generation. Preprint at arXiv. https://doi.org/10.48550/arXiv.2112.01651.

417. Das, D., Das, B., and Mahesh, K. (2016). A computational analysis of mahabharata. In Proceedings of the 13th International Conference on Natural Language Processing, pp. 219–228.

418. Escartín, C.P., Reijers, W., Lynn, T., Moorkens, J., Way, A., and Liu, C.H. (2017). Ethical considerations in NLP shared tasks. In Proceedings of the First Workshop on Ethics in Natural Language Processing (Association for Computational Linguistics), pp. 66–73. https://doi.org/10.18653/v1/W17-1608.

419. GitHub, Inc. Github. https://github.com/.

420. CERN/OpenAIRE. Zenodo. https://zenodo.org/.

421. Fitsilis, F., and Mikros, G. (2021). Development and validation of a corpus of written parliamentary questions in the hellenic parliament. J. Open Human. Data *7*, 18. https://doi.org/10.5334/johd.45.

422. Cao, H., Dodge, J., Lo, K., McFarland, D.A., and Wang, L.L. (2023). The rise of open science: Tracking the evolution and perceived value of data and methods link-sharing practices. Preprint at arXiv. https://doi.org/10.48550/arXiv.2310.03193.

423. Dritsa, K., Thoma, K., Pavlopoulos, J., and Louridas, P. (2022). A Greek parliament proceedings dataset for computational linguistics and political analysis. Zenodo. https://doi.org/10.5281/zenodo.6626315.

424. Fitsilis, F., and Mikros, G. (2021). Corpus of parliamentary questions in the hellenic parliament. Zenodo. https://doi.org/10.5281/zenodo.4747451.

425. Prokopidis, P., and Piperidis, S. (2020). Resources for the paper "a neural NLP toolkit for Greek" (setn-2020). Institute for Language and Speech Processing (ILSP). http://nlp.ilsp.gr/setn-2020/.

426. Barzokas, V., Papagiannopoulou, E., and Tsoumakas, G. (2024). Time-stamped Greek corpus. GitHub. https://github.com/intelligence-csd-auth-gr/greek-words-evolution.git.

427. Iosif, E., Georgiladakis, S., and Potamianos, A. (2016). Greek Web Document Snippets Dataset (Technical University of Crete). http://www.telecom.tuc.gr/~iosife/downloads/adsms/actindex.html/.

428. Majliš, M., and Žabokrtský, Z. (2012). Language richness of the web. In Proceedings of the Eighth International Conference on Language Resources and Evaluation (LREC'12), pp. 2927–2934.

429. Project Gutenberg. Project gutenberg. https://www.gutenberg.org/.

430. Openbook.gr Community. Openbook.gr. https://www.openbook.gr/.

431. Center for the Greek Language (2007). Corpus of the newspaper "macedonia". Center for the Greek Language. http://www.greek-language.gr/greekLang/modern_greek/tools/corpora/makedonia/content.html.

432. Fokides, E., and Peristeraki, E. (2025). Comparing ChatGTP's correction and feedback comments with that of educators in the context of primary students' short essays written in English and Greek. Educ. Inf. Technol. *30*, 2577–2621. https://doi.org/10.1007/s10639-024-12912-8.

433. Bakagianni, J., Pouli, K., Gavriilidou, M., and Pavlopoulos, J. (2025). Greek-NLP/survey: A systematic survey of natural language processing research for the Greek language. Zenodo. URL: https://doi.org/10.5281/zenodo.15314882.

Patterns, Volume *6*

Supplemental information

A systematic survey of natural language processing for the Greek language

Juli Bakagianni, Kanella Pouli, Maria Gavriilidou, and John Pavlopoulos

Supplemental Notes

Note S1. Quality Assurance Search Round

The search strategy for retrieving publications on Greek Natural Language Processing (NLP) research involved querying three databases using their APIs, with the query terms Greek (or Modern Greek) and “natural language processing” for the period January 2012 to December 2023 (Section Search Protocol).

To ascertain that we performed a comprehensive search, we conducted an additional round of searches on Google Scholar for the same time period. Google Scholar does not provide an API for automated data retrieval, which is why it was not included in the core search rounds. This supplementary search employed more specific query terms aimed at broadening our exploration, including specific NLP downstream tasks alongside “Greek” and either “Natural Language Processing” or “NLP”. Table S1 summarizes this supplementary search process.

We performed 23 queries, employing various alternatives, acronyms, and operators for selected NLP tasks from November 6th, 2023, to January 12th, 2024. Whenever the retrieved papers numbered less than 100, all were examined; otherwise, only the initial thirty were reviewed. The count of the top 30 papers seemed adequate, as beyond these, the subsequent papers didn’t appear to be relevant.

Table S1: The queries and the number of papers retrieved from Google Scholar during the quality assurance search round.

Query	Number of papers
“toxicity detection” “natural language processing” greek	64
“toxicity detection” greek nlp	70
“toxicity detection” + greek + nlp	40
“toxicity detection” and “greek” and “nlp”	32
“abusive language” “natural language processing” greek	420
“hateful language” “natural language processing” greek	48
“aggressive language” “natural language processing” greek	44
“authorship attribution” “natural language processing” greek	803
“authorship identification” “natural language processing” greek	330
“authorship analysis” “natural language processing” greek	324
“authorship detection” “natural language processing” greek	61
“sentiment analysis” greek nlp	7,490
“sentiment analysis” “natural language processing” greek	7,050
“sentiment analysis” “greek” “nlp”	2,430
“machine translation” greek nlp	9,160
“machine translation” “greek” “nlp”	3,750
“named entity recognition” “natural language processing” greek	4350
“named entity recognition” greek	5,550
“question answering” “natural language processing” greek	4,840
summarization “natural language processing” greek	16,600
semantics “natural language processing” greek	17,100
syntax “natural language processing” greek	14,400

The queries led us to review 698 publications. After removing duplicates both within the same round and across previous core search rounds, 357 publications were selected. Following the search process of the protocol, we kept those publications that referenced the term “Greek” in either the title or abstract. Additionally, for this particular round, we refined our approach to the term “Natural Language Processing” by including only those publications that explicitly mentioned the term within the publication’s content, excluding those where it appeared only in the references. In the subsequent filtering process (see Section Filtering Strategy), applying the exclusion criteria yielded five additional publications, which were included in the final list of surveyed papers.

Note S2. Natural Language Understanding and Generation

This Appendix first discusses Natural Language Understanding (NLU), which enables machines to understand natural language, and then Natural Language Generation (NLG), which is the process of generating meaningful text.[1]

Note S2.1. Natural Language Understanding

NLU enables machines to understand, interpret, and derive meaning from human language in a way that is both accurate and contextually relevant. It addresses a wide range of linguistic phenomena, from lexical semantics concerning aspects of word meaning to the high-level reasoning and application of world knowledge.[2] These linguistic phenomena are foundational for various NLP tasks, as they provide the essential linguistic comprehension needed to analyse the textual data.[3]

Traditional machine learning approaches represent texts by engineered features (e.g., based on POS tags or lexicons) or by character and word embeddings, encoding the linguistic knowledge into the input, before solving the task at hand. In the deep learning (DL) era, on the other hand, linguistic skills are encoded in neural network models via language modeling.[4] Therefore they are considered as an evaluation criterion, by assessing language models (LM) on datasets annotated for various linguistic phenomena. General-purpose NLU evaluation benchmark datasets exist primarily for English,[2,5? ? –7] but also for other languages.[8–13] Unfortunately, such monolingual NLU benchmarks are currently unavailable for Greek, based on our survey. There are multilingual NLU benchmarks, where the non-English language parts are translated either by humans, such as Belebele,[14] or automatically, such as ARC Greek.[?] However, even manual translations are distinct from naturally occurring data created by native speakers due to various factors, such as cultural nuances, idiomatic expressions, and context-specific references in native speakers' natural language usage.[15]

Note S2.2. Natural Language Generation

NLG involves the generation of human understandable natural language text from various data formats, such as text, structured data, video, and audio. NLG techniques are used in many downstream tasks, such as summarization, dialogue generation, Question Answering (QA), and Machine Translation (MT). Advancements in DL, particularly with Transformer-based LMs such as BART[16] (which follows the encoder-decoder neural architecture) and GPT-2[17] (which uses a decoder-only architecture), as well as the latest developments in large language models, such as the GPT, Llama, and Palm families, have fueled rapid progress in NLG (Section The DL Era). Alongside the advancement of NLG models, attention toward their limitations and potential risks has also increased.[18] An example is output degeneration,[19,20] which refers to generated output that is bland, incoherent, or gets stuck in repetitive loops. Another example is the generation of nonsensical text, or text that is unfaithful to the provided source input,[21,22] also known as hallucination.[23]

Note S3. Sentiment Analysis and Argument Mining

Sentiment, opinion, and emotion can be approached by various annotation schemas. Our work presents only the ones employed in the retrieved papers. Binary Sentiment Analysis (SA), where the text's polarity is either positive or negative, is the primary approach used in most papers cited in this study.[24–35] However, several studies[26,29,36–39] added a neutral class for texts carrying no opinion.

Five-class sentiment classification[40–43] increases the granularity, with scales from positive to negative. Subjectivity detection can precede SA, by addressing the binary task of whether the text comprises sentiment or not.[44] Instead of the coarse level of sentiment and subjectivity, however, one can also focus on fine-grained emotions,[37,44–46] using different emotion categories that have been suggested by theorists in psychology.[47? ? ?] Ekman has identified six emotions, i.e., anger, disgust, fear, joy, sadness, and surprise, as primary. A common approach, finally, concerns the selection of a single emotion or sentiment that is related to a specific condition, such as the anxiety during the COVID19 pandemic[27] or sarcasm/irony.[37,48,49]

A critical distinction between SA approaches is the level of granularity of the analysis. The coarser granularity concerns the document level, where the overall sentiment of an entire document is assessed. A finer granularity regards the sentence level, where the sentiment of short texts, such as tweets or the individual sentences within a document, is analyzed. SA can also be aspect-based, where the sentiment towards specific aspects or features of an entity is evaluated. These are described in detail below:

- **Document-level SA** involves the detection of the emotions expressed in an opinion document (e.g., a lengthy product review) or its classification as positive or negative (i.e., the sentiment polarity). Analysis at this level focuses on the sentiment of the entire document, as a whole (e.g., a product review), without considering entities or aspects within the document.

- **Sentence-level SA** concerns short texts, such as tweets or the sentences of a document. Although analysis at the sentence level is similar to that at the document level, the former can be more challenging due to the limited context contained. Also, classification at the sentence level cannot easily ignore the neutral class, because sentences with no opinion are more likely to appear. That is, even opinionated documents comprise sentences that bear no sentiment. Furthermore, a common (although implicit) assumption at research tasks

focusing on the sentence level is that each sentence expresses a single sentiment,[50] which, however, is not always the case.

- **Aspect-based SA**, also known as topic-based, entity-based, or target-based SA, as there is not a single term that sounds natural in every application domain,[51] is the analysis that focuses on the sentiment expressed in a document or sentence with respect to a specific aspect of an entity. For example, in the sentence "iPhone's battery lasts long" the entity is iPhone and the aspect is battery. Closely related to aspect-based SA is the **stance detection** task,[52] which aims to identify the stance (as in favor of or against) of the text author towards a target (person, organization, movement, policy, etc.) either explicitly mentioned or implied within the text.[53,54] Only a few published studies concern SA on Greek at this granularity.
- **Argument Mining** is the task of extracting natural language arguments and their relationships from text. The final objective is to provide machine-processable structured data for computational models of argument, to facilitate the automatic identification of reasoning capabilities upon the retrieved arguments and relations.[55]

Note S4. Toxicity Detection

Our study uses the term toxicity as a broader umbrella term of unwanted user-generated content including hateful and violent speech.[56] Although no standard definition exists today and some researchers are against oversimplifying solutions,[57] our choice is driven simply by the practical need to cover all the subtypes in a single name. However, the definition of toxicity itself is controversial: different cultures, languages, dialects, social groups, minority groups do not necessarily agree on what constitutes toxicity; furthermore, aggressive language use is not always toxic, e.g., inside a group, swear words can function as solidarity markers.

Supplemental References

1. Khurana, D., Koli, A., Khatter, K., and Singh, S. (2023). Natural language processing: State of the art, current trends and challenges. Multimedia tools and applications *82*, 3713–3744. doi: `https://doi.org/10.1007/s11042-022-13428-4`.
2. Wang, A., Singh, A., Michael, J., Hill, F., Levy, O., and Bowman, S. (2018). GLUE: A multi-task benchmark and analysis platform for natural language understanding. In T. Linzen, G. Chrupała, and A. Alishahi, eds. Proceedings of the 2018 EMNLP Workshop BlackboxNLP: Analyzing and Interpreting Neural Networks for NLP. Brussels, Belgium: Association for Computational Linguistics pp. 353–355. doi: `10.18653/v1/W18-5446`.
3. Bowman, S.R., and Dahl, G. (2021). What will it take to fix benchmarking in natural language understanding? In K. Toutanova, A. Rumshisky, L. Zettlemoyer, D. Hakkani-Tur, I. Beltagy, S. Bethard, R. Cotterell, T. Chakraborty, and Y. Zhou, eds. Proceedings of the 2021 Conference of the North American Chapter of the Association for Computational Linguistics: Human Language Technologies. Online: Association for Computational Linguistics pp. 4843–4855. doi: `10.18653/v1/2021.naacl-main.385`.
4. Belinkov, Y., Gehrmann, S., and Pavlick, E. (2020). Interpretability and analysis in neural nlp. In Proceedings of the 58th annual meeting of the association for computational linguistics: tutorial abstracts. pp. 1–5.
5. Bommasani, R., Liang, P., and Lee, T. (2023). Holistic evaluation of language models. Annals of the New York Academy of Sciences *1525*, 140–146.
6. Chen, Z., and Gao, Q. (2022). Curriculum: A broad-coverage benchmark for linguistic phenomena in natural language understanding. In M. Carpuat, de M.C. Marneffe, and I.V. Meza Ruiz, eds. Proceedings of the 2022 Conference of the North American Chapter of the Association for Computational Linguistics: Human Language Technologies. Seattle, United States: Association for Computational Linguistics pp. 3204–3219. doi: `10.18653/v1/2022.naacl-main.234`.
7. Nie, Y., Williams, A., Dinan, E., Bansal, M., Weston, J., and Kiela, D. (2019). Adversarial nli: A new benchmark for natural language understanding. Preprint at arXiv `https://doi.org/10.48550/arXiv.1910.14599`.
8. Urbizu, G., San Vicente, I., Saralegi, X., Agerri, R., and Soroa, A. (2022). Basqueglue: A natural language understanding benchmark for basque. In Proceedings of the Thirteenth Language Resources and Evaluation Conference. pp. 1603–1612.

9. Shavrina, T., Fenogenova, A., Anton, E., Shevelev, D., Artemova, E., Malykh, V., Mikhailov, V., Tikhonova, M., Chertok, A., and Evlampiev, A. (2020). RussianSuperGLUE: A Russian language understanding evaluation benchmark. In B. Webber, T. Cohn, Y. He, and Y. Liu, eds. Proceedings of the 2020 Conference on Empirical Methods in Natural Language Processing (EMNLP). Online: Association for Computational Linguistics pp. 4717–4726. doi: 10.18653/v1/2020.emnlp-main.381.

10. Wilie, B., Vincentio, K., Winata, G.I., Cahyawijaya, S., Li, X., Lim, Z.Y., Soleman, S., Mahendra, R., Fung, P., Bahar, S., and Purwarianti, A. (2020). IndoNLU: Benchmark and resources for evaluating Indonesian natural language understanding. In K.F. Wong, K. Knight, and H. Wu, eds. Proceedings of the 1st Conference of the Asia-Pacific Chapter of the Association for Computational Linguistics and the 10th International Joint Conference on Natural Language Processing. Suzhou, China: Association for Computational Linguistics pp. 843–857.

11. Xu, L., Hu, H., Zhang, X., Li, L., Cao, C., Li, Y., Xu, Y., Sun, K., Yu, D., Yu, C. et al. (2020). Clue: A chinese language understanding evaluation benchmark. In Proceedings of the 28th International Conference on Computational Linguistics. pp. 4762–4772.

12. Rybak, P., Mroczkowski, R., Tracz, J., and Gawlik, I. (2020). KLEJ: Comprehensive benchmark for Polish language understanding. In D. Jurafsky, J. Chai, N. Schluter, and J. Tetreault, eds. Proceedings of the 58th Annual Meeting of the Association for Computational Linguistics. Online: Association for Computational Linguistics pp. 1191–1201. doi: 10.18653/v1/2020.acl-main.111.

13. Ham, J., Choe, Y.J., Park, K., Choi, I., and Soh, H. (2020). Kornli and korsts: New benchmark datasets for korean natural language understanding. In Findings of the Association for Computational Linguistics: EMNLP 2020. pp. 422–430.

14. Bandarkar, L., Liang, D., Muller, B., Artetxe, M., Shukla, S.N., Husa, D., Goyal, N., Krishnan, A., Zettlemoyer, L., and Khabsa, M. (2023). The belebele benchmark: a parallel reading comprehension dataset in 122 language variants. Preprint at arXiv https://doi.org/10.48550/arXiv.2308.16884.

15. Rogers, A., Gardner, M., and Augenstein, I. (2023). Qa dataset explosion: A taxonomy of nlp resources for question answering and reading comprehension. ACM Comput. Surv. *55*, 1–45. doi: 10.1145/3560260.

16. Lewis, M., Liu, Y., Goyal, N., Ghazvininejad, M., Mohamed, A., Levy, O., Stoyanov, V., and Zettlemoyer, L. (2020). BART: Denoising sequence-to-sequence pre-training for natural language generation, translation, and comprehension. In Proceedings of the 58th Annual Meeting of the Association for Computational Linguistics. Online: Association for Computational Linguistics pp. 7871–7880.

17. Radford, A., Wu, J., Child, R., Luan, D., Amodei, D., Sutskever, I. et al. (2019). Language models are unsupervised multitask learners. OpenAI. https://openai.com/blog/language-unsupervised.

18. Ji, Z., Lee, N., Frieske, R., Yu, T., Su, D., Xu, Y., Ishii, E., Bang, Y.J., Madotto, A., and Fung, P. (2023). Survey of hallucination in natural language generation. ACM Computing Surveys *55*, 1–38. doi: https://doi.org/10.1145/3571730.

19. Holtzman, A., Buys, J., Du, L., Forbes, M., and Choi, Y. (2019). The curious case of neural text degeneration. Preprint at arXiv https://doi.org/10.48550/arXiv.1904.09751.

20. Welleck, S., Kulikov, I., Roller, S., Dinan, E., Cho, K., and Weston, J. (2019). Neural text generation with unlikelihood training. Preprint at arXiv https://doi.org/10.48550/arXiv.1908.04319.

21. Raunak, V., Menezes, A., and Junczys-Dowmunt, M. (2021). The curious case of hallucinations in neural machine translation. Preprint at arXiv https://doi.org/10.48550/arXiv.2104.06683.

22. Rohrbach, A., Hendricks, L.A., Burns, K., Darrell, T., and Saenko, K. (2018). Object hallucination in image captioning. Preprint at arXiv https://doi.org/10.48550/arXiv.1809.02156.

23. Maynez, J., Narayan, S., Bohnet, B., and McDonald, R. (2020). On faithfulness and factuality in abstractive summarization. Preprint at arXiv https://doi.org/10.48550/arXiv.2005.00661.

24. Bilianos, D. (2022). Experiments in text classification: Analyzing the sentiment of electronic product reviews in greek. Journal of Quantitative Linguistics *29*, 374–386. doi: 10.1080/09296174.2021.1885872.

25. Kapoteli, E., Koukaras, P., and Tjortjis, C. (2022). Social media sentiment analysis related to covid-19 vaccines: Case studies in english and greek language. In I. Maglogiannis, L. Iliadis, J. Macintyre, and P. Cortez, eds. Artificial Intelligence Applications and Innovations. Cham: Springer International Publishing. ISBN 978-3-031-08337-2 pp. 360–372. doi: https://doi.org/10.1007/978-3-031-08337-2_30.

26. Alexandridis, G., Varlamis, I., Korovesis, K., Caridakis, G., and Tsantilas, P. (2021). A survey on sentiment analysis and opinion mining in greek social media. Information *12*. doi: 10.3390/info12080331.

27. Kydros, D., Argyropoulou, M., and Vrana, V. (2021). A content and sentiment analysis of greek tweets during the pandemic. Sustainability *13*, 6150. doi: 10.3390/su13116150.

28. Braoudaki, A., Kanellou, E., Kozanitis, C., and Fatourou, P. (2020). Hybrid data driven and rule based sentiment analysis on greek text. Procedia Computer Science *178*, 234–243. doi: https://doi.org/10.1016/j.procs.2020.11.025. 9th International Young Scientists Conference in Computational Science, YSC2020, 05-12 September 2020.

29. Beleveslis, D., Tjortjis, C., Psaradelis, D., and Nikoglou, D. (2019). A hybrid method for sentiment analysis of election related tweets. In 2019 4th South-East Europe Design Automation, Computer Engineering, Computer Networks and Social Media Conference (SEEDA-CECNSM). pp. 1–6. doi: 10.1109/SEEDA-CECNSM.2019.8908289.

30. Medrouk, L., and Pappa, A. (2018). Do deep networks really need complex modules for multilingual sentiment polarity detection and domain classification? In 2018 International Joint Conference on Neural Networks (IJCNN). pp. 1–6. doi: 10.1109/IJCNN.2018.8489613.

31. Medrouk, L., and Pappa, A. (2017). Deep learning model for sentiment analysis in multi-lingual corpus. In D. Liu, S. Xie, Y. Li, D. Zhao, and E.S.M. El-Alfy, eds. Neural Information Processing. Cham: Springer International Publishing. ISBN 978-3-319-70087-8 pp. 205–212. doi: https://doi.org/10.1007/978-3-319-70087-8_22.

32. Athanasiou, V., and Maragoudakis, M. (2017). A novel, gradient boosting framework for sentiment analysis in languages where nlp resources are not plentiful: A case study for modern greek. Algorithms *10*. doi: 10.3390/a10010034.

33. Giatsoglou, M., Vozalis, M.G., Diamantaras, K., Vakali, A., Sarigiannidis, G., and Chatzisavvas, K.C. (2017). Sentiment analysis leveraging emotions and word embeddings. Expert Systems with Applications *69*, 214–224. doi: https://doi.org/10.1016/j.eswa.2016.10.043.

34. Charalampakis, B., Spathis, D., Kouslis, E., and Kermanidis, K. (2016). A comparison between semi-supervised and supervised text mining techniques on detecting irony in greek political tweets. Engineering Applications of Artificial Intelligence *51*, 50–57. doi: https://doi.org/10.1016/j.engappai.2016.01.007. Mining the Humanities: Technologies and Applications.

35. Markopoulos, G., Mikros, G., Iliadi, A., and Liontos, M. (2015). Sentiment analysis of hotel reviews in greek: A comparison of unigram features. In V. Katsoni, ed. Cultural Tourism in a Digital Era. Cham: Springer International Publishing. ISBN 978-3-319-15859-4 pp. 373–383.

36. Drakopoulos, G., Giannoukou, I., Mylonas, P., and Sioutas, S. (2020). A graph neural network for assessing the affective coherence of twitter graphs. In 2020 IEEE International Conference on Big Data (Big Data). IEEE pp. 3618–3627. doi: 10.1109/BigData50022.2020.9378492.

37. Tsakalidis, A., Papadopoulos, S., Voskaki, R., Ioannidou, K., Boididou, C., Cristea, A.I., Liakata, M., and Kompatsiaris, Y. (2018). Building and evaluating resources for sentiment analysis in the greek language. Language resources and evaluation *52*, 1021–1044. doi: https://doi.org/10.1007/s10579-018-9420-4.

38. Makrynioti, N., and Vassalos, V. (2015). Sentiment extraction from tweets: Multilingual challenges. In S. Madria, and T. Hara, eds. Big Data Analytics and Knowledge Discovery. Cham: Springer International Publishing. ISBN 978-3-319-22729-0 pp. 136–148. doi: https://doi.org/10.1007/978-3-319-22729-0_11.

39. Petasis, G., Spiliotopoulos, D., Tsirakis, N., and Tsantilas, P. (2014). Sentiment analysis for reputation management: Mining the greek web. In Artificial Intelligence: Methods and Applications: 8th Hellenic Conference on AI, SETN 2014, Ioannina, Greece, May 15-17, 2014. Proceedings 8. Springer pp. 327–340. doi: https://doi.org/10.1007/978-3-319-07064-3_26.

40. Spatiotis, N., Perikos, I., Mporas, I., and Paraskevas, M. (2020). Sentiment analysis of teachers using social information in educational platform environments. International Journal on Artificial Intelligence Tools *29*, 1–29. doi: https://doi.org/10.1142/S0218213020400047.

41. Spatiotis, N., Perikos, I., Mporas, I., and Paraskevas, M. (2019). Examining the impact of discretization technique on sentiment analysis for the greek language. In 2019 10th International Conference on Information, Intelligence, Systems and Applications (IISA). pp. 1–6. doi: 10.1109/IISA.2019.8900699.

42. Spatiotis, N., Paraskevas, M., Perikos, I., and Mporas, I. (2017). Examining the impact of feature selection on sentiment analysis for the greek language. In Speech and Computer: 19th International Conference, SPECOM 2017, Hatfield, UK, September 12-16, 2017, Proceedings 19. Springer pp. 353–361. doi: https://doi.org/10.1007/978-3-319-66429-3_34.

43. Spatiotis, N., Mporas, I., Paraskevas, M., and Perikos, I. (2016). Sentiment analysis for the greek language. In Proceedings of the 20th Pan-Hellenic Conference on Informatics. PCI '16. New York, NY, USA: Association for Computing Machinery. ISBN 9781450347891 pp. 1–4.

44. Solakidis, G.S., Vavliakis, K.N., and Mitkas, P.A. (2014). Multilingual sentiment analysis using emoticons and keywords. In 2014 IEEE/WIC/ACM International Joint Conferences on Web Intelligence (WI) and Intelligent Agent Technologies (IAT) vol. 2. IEEE pp. 102–109. doi: 10.1109/WI-IAT.2014.86.

45. Alexandridis, G., Korovesis, K., Varlamis, I., Tsantilas, P., and Caridakis, G. (2022). Emotion detection on greek social media using bidirectional encoder representations from transformers. In 25th Pan-Hellenic Conference on Informatics. PCI 2021. New York, NY, USA: Association for Computing Machinery. ISBN 9781450395557 pp. 28–32. doi: 10.1145/3503823.3503829.

46. Chatzakou, D., Vakali, A., and Kafetsios, K. (2017). Detecting variation of emotions in online activities. Expert Systems with Applications *89*, 318–332. doi: https://doi.org/10.1016/j.eswa.2017.07.044.

47. Kafetsios, K., and Nezlek, J.B. (2012). Emotion and support perceptions in everyday social interaction: Testing the "less is more" hypothesis in two cultures. Journal of Social and Personal Relationships *29*, 165–184. doi: https://doi.org/10.1177/0265407511420194.

48. Antonakaki, D., Spiliotopoulos, D., V. Samaras, C., Pratikakis, P., Ioannidis, S., and Fragopoulou, P. (2017). Social media analysis during political turbulence. PloS one *12*, e0186836. doi: https://doi.org/10.1371/journal.pone.0186836.

49. Antonakaki, D., Spiliotopoulos, D., Samaras, C.V., Ioannidis, S., and Fragopoulou, P. (2016). Investigating the complete corpus of referendum and elections tweets. In 2016 IEEE/ACM International Conference on Advances in Social Networks Analysis and Mining (ASONAM). IEEE pp. 100–105. doi: 10.1109/ASONAM.2016.7752220.

50. Liu, B. (2020). Sentiment analysis: Mining opinions, sentiments, and emotions. Cambridge university press.

51. Schouten, K., and Frasincar, F. (2015). Survey on aspect-level sentiment analysis. IEEE Transactions on Knowledge and Data Engineering *28*, 813–830. doi: 10.1109/TKDE.2015.2485209.

52. Küçük, D., and Can, F. (2020). Stance detection: A survey. ACM Computing Surveys (CSUR) *53*, 1–37. doi: https://doi.org/10.1145/3369026.

53. Mohammad, S., Kiritchenko, S., Sobhani, P., Zhu, X., and Cherry, C. (2016). Semeval-2016 task 6: Detecting stance in tweets. In Proceedings of the 10th international workshop on semantic evaluation (SemEval-2016). pp. 31–41. doi: 10.18653/v1/S16-1003.

54. Sobhani, P. Stance detection and analysis in social media. Ph.D. thesis Universite d'Ottawa/University of Ottawa (2017).

55. Cabrio, E., and Villata, S. (2018). Five years of argument mining: A data-driven analysis. In IJCAI vol. 18. pp. 5427–5433. doi: https://doi.org/10.24963/ijcai.2018/766.

56. Waseem, Z., Davidson, T., Warmsley, D., and Weber, I. (2017). Understanding abuse: A typology of abusive language detection subtasks. Preprint at arXiv https://doi.org/10.48550/arXiv.1705.09899.

57. Díaz, M., Amironesei, R., Weidinger, L., and Gabriel, I. (2022). Accounting for offensive speech as a practice of resistance. In Proceedings of the Sixth Workshop on Online Abuse and Harms (WOAH). pp. 192–202.
